# Migration is associated with baseline severity and progress over time in autism spectrum disorder: Evidence from a French prospective longitudinal study

**DOI:** 10.1371/journal.pone.0272693

**Published:** 2022-10-06

**Authors:** Carlotta Bettencourt, Nicole Garret-Gloanec, Hugues Pellerin, Morgane Péré, Maria Squillante, Fabienne Roos-Weil, Léa Ferrand, Anne-Sophie Pernel, Gisèle Apter, David Cohen

**Affiliations:** 1 Groupe Hospitalo-Universitaire Pitié-Salpêtrière, Institut des pathologies du Développement de l’Enfant et de l’AdoLescent (IDEAL), APHP. SU, Paris, France; 2 Centre National de la Recherche Scientifique, Unité Mixte de Recherche 7222, Institut des Systèmes Intelligents et Robotiques, Sorbonne Université, Paris, France; 3 Centre Nantais de Parentalité, 1 rue Marmontel, Centre Hospitalo-Universitaire, Nantes, France; 4 Direction de la recherche, Plateforme de Méthodologie et Biostatistique, Centre Hospitalo-Universitaire de Nantes, Nantes, France; 5 49 rue de Siam, 29200 Brest, France; 6 Centre Médico-Psychologique, Paris, France; 7 Direction de la Recherche—Département promotion, Centre Hospitalo-Universitaire de Nantes, Nantes, France; 8 CESAME, Centre Roger Misès, Angers, France; 9 Groupe Hospitalier du Havre, Université de Rouen Normandie, Le Havre, France; PLOS ONE, UNITED KINGDOM

## Abstract

**Background:**

The prevalence of autism-spectrum disorder (ASD) has been shown to be higher in migrant families, but it is also a challenge for health care professionals to offer adequate services to families that face multiple challenges. In the context of the EPIGRAM study (a French prospective, multisite, longitudinal observational study implementing integrative care practices (ICPs) for children with ASD), we aimed to assess the impact of migration on children with ASD.

**Method and findings:**

89 children with ASD aged 3 to 6 years old (92% males) were recruited and followed up for 12 months. The children were clinically assessed using several instruments. At baseline, children had severe autism on average on the Children Autism Rating Scale (CARS, mean = 44; SD = 6.51) and moderate autism on the PsychoEducational profile-3-R (PEP-3-R) maladaptive behavior category (mean = 30; SD = 29.89). Thirty percent of the families had a low socio-economic status, and 56% were first-generation immigrants. For all clinical variables, children of immigrant parents had more severe autism and developmental delays at baseline. A linear mixed model established an improvement in all clinical characteristics over the 12 months of the study. This trend may be attributed to ICPs or any naturally occurring event during that period. Families shared this positive view over time. However, the improvements were slower for two clinical dimensions of the PEP-3-R in children from migrant families. For the inappropriate behavior category, the time effect diminished by an average of 0.83 percentile/month for children whose parents were migrants vs. children whose parents were non-migrants. Similarly, for verbal behavior characteristics, the time effect diminished by an average of 1.32 percentile/month for children whose parents were migrants vs. children whose parents were non-migrants.

**Conclusion:**

Despite an overall positive improvement, we found that migration is associated baseline severity and progress over time in children with ASD. There is an urgent need to target the migrant population with specific research and understand the avenues that carry such higher severity.

**Clinical trial registration:**

Study registration on clinicaltrials.gov under the number NCT02154828.

## Introduction

In the DSM-5, autism spectrum disorder (ASD) is defined as a neurodevelopmental disorder that manifests in early childhood. It is characterized by persistent deficits in social interaction and communication across multiple contexts associated with restricted, repetitive patterns of behaviors and interests or hypo/hyper-reactivity to sensory stimuli [1]. ASD morbidity is significant and can impact social, occupational and other important areas of functioning.

Many studies have established a higher prevalence of ASD among migrant populations [2]. However, the way in which migration affects ASD remains inconclusive due to insufficient research. Likewise, in France, immigrant families are more likely to report ASD than native families: children of migrant backgrounds appear to be at an increased risk of autism [3] and often show more severe autism symptomatology and comorbid intellectual disorder (ID) [4]. Despite the need to receive appropriate interventions in time [5], immigrant parents of children with ASD often fail to receive timely care due to several barriers in the health care system [6]. Thus, members of minority groups do not tend to receive evidence-based interventions at appropriate ages, which points to a significant gap in equity in relation to the cross-ethnic and cross-racial treatment of ASD [7]. Additionally, in France, the higher prevalence of ASD among French immigrants is associated with higher levels of stress, adversity and trauma experienced in the home country [8].

To understand the impact that migration can have on child development and ASD specifically, one needs to take into account immigration trends in France. In 2018, 6.5 million immigrants were living in France, which is equivalent to 9.7% of the population [9]. One of the main reasons individuals migrate to France is to find better employment and socioeconomic opportunities [8]. Immigrant populations in France come from across the globe, but a majority trace their origins to African nations in North and Sub-Saharan Africa: in 2018, 13% of the immigrants were born in Algeria, 11.9% were born in Morocco, 9.2% were born in Portugal, and 4.4% were born in Tunisia [9]. Immigrants in France generally have poorer living conditions if they do not have the social capital to access services such as health care and education. France offers universal health care coverage for its citizens, regardless of age, wealth, and social status. However, immigrant families often face barriers that limit their access to health care [10]. (1) The difference in cultural background affects both understanding of the health care system and patient-caregiver relations [11, 12]. (2) Residency status complicates access to health care [13]. For example, migrant families of children with ASD report less satisfaction with the health care system in France [10]. (3) Linguistic and educational barriers are also significant for migrant parents. The minority and low-income status of families with ASD individuals leads to a situation in which migrant, impoverished and minority children with ASD suffer from structural discrimination in their access to health care [14]. Various clinical frameworks have been put in place in the French health care system to bridge the gap between immigrant patients and nonimmigrant doctors, facilitating communication between the two parties. These frameworks are designed to accommodate immigrant families of all proficiency levels, including in terms of language. They also take into consideration the cultural influences of individuals and the impacts of these cultural effects on reporting [11]. (4) Finally, the socioeconomic factors that co-occur with ASD and migration can affect access to care [2]. In France, the largest epidemiological studies also pointed out that a higher prevalence of ASD was noted among unemployed adults, with no higher education, and single-parent families.

Children of immigrant parents are likely to receive their autism diagnoses later in life and are less likely to receive appropriate care and support for their condition. This means that members of minority groups do not tend to receive evidence-based interventions at appropriate ages and that parents have less access to relevant services for their children, which here represents a significant gap in equity in relation to the cross-ethnic and cross-racial treatment of autism and ASD [15]. Researchers and practitioners must develop more intercultural competency in providing care to ASD migrant children and their families [16]. This work is indicative of the need for further research on the efficacy of autism treatment within these demographic groups.

Early interventions that have been shown to be effective in randomized controlled trials (RCTs) are based on educational, behavioral and developmental approaches. The main interventions are early intensive behavioral intervention (EIBI) [17], the Early Start Denver Model (ESDM) [18], the Exchange and Development Therapy (EDT) [19] and Joint Attention Symbolic Play Engagement and Regulation (JASPER) [20]. Care for children with ASD should also include parent-mediated interventions (e.g., The Preschool Autism Communication Trial—PACT [21]). These have been initially included in the Treatment and Education of Autistic and Communication Related Handicapped Children (TEACCH) program, which has shown minor effects in meta-analyses [22]. Furthermore, educational interventions and specific therapies (e.g., speech therapy, occupational therapy, psychotherapy) can also be found in ASD care [23]. In this respect, parents play a critical role in relation to autism treatment. With the addition of a one-and-a-half hour per week parental coaching intervention, toddlers with ASD were found to develop higher levels of verbal and nonverbal demonstrations of cognition and to demonstrate less irritability and fewer outbursts in relation to common triggers [24]. In turn, the modification of the ASD child’s environment to include elements bringing about autonomous interest and motivation is also in line with improving verbal and nonverbal cognition. This produces a context in which targeted environmental modification, acting as an adjunct to other forms of intervention, must be viewed as a critical element of successful integrative interventions [25]. Naturalistic developmental behavioral interventions (NDBIs) could be the most promising according to a recent therapeutic review taking into account study quality indicators [26]. However, despite the growing body of evidence from RCTs, which best protects against bias, clinicians are facing several issues in everyday practice to recommend the aforementioned treatments. First, there is insufficient evidence to determine which specific interventions are the most effective treatments for children with ASD, given the heterogeneity of the disorder [27]. Secondly, the accessibility is highly variable across countries. For example, in Europe, a large survey reported considerable variation in the use of early interventions. Nine percent of children received no intervention at all, and the rate was above 10% in the Netherlands, Denmark, Ireland, the United Kingdom, the Czech Republic, and Macedonia. The most frequently reported well-defined interventions were speech and language therapy (64%), parent training (38.2%), occupational therapy (34.8%), behavioral intervention (32.3%), and developmental intervention (22.9%). Interventions were often combined, and more than 40% of the parents also reported less defined therapeutic interventions [28].

From a broader view, the treatment that can be offered to children with ASD depends on both macro- and micro-variables. Macro-variables include the healthcare system, the financial support available for low-income families, and the school system’s openness to accommodating children with special educational needs. Micro-variables include the socio-economic background, migrant status, time of diagnosis, and severity of any comorbid intellectual disability (ID) [28, 29]. Additionally, most of the common ASD comorbidities, notably epilepsy, sleep disorders and problematic gastrointestinal and immune functioning, must also be addressed because these comorbidities can generate problematic patterns of irritability and drain the cognitive abilities of individual children with ASD [30].

Currently, in France, the healthcare system is completely free to access, including for migrant families that have no insurance or political status. Overall, 94.1% of children with ASD receive an intervention [28], and 88% of them attend school. More severe adaptive and cognitive deficits are associated with less or no schooling [17]. In addition, school-age children with ASD and moderate to severe ID usually do not receive educational programming services in mainstream classrooms or part-time private practice (as is the case in the US) but receive institutional care in specialized outpatient health care organizations (e.g., day-care hospitals). Services are given according to children’s needs and include individual and group therapeutic approaches (e.g., speech therapy, sensorimotor group therapy, and play therapy). In addition to the individualization of treatment, the place where the treatment occurs can also support more vulnerable families and children. These therapeutic approaches are often under the umbrella of integrative care practices (ICPs), in which a combination of interventions is the rule rather than a single therapeutic package.

EPIGRAM is a French prospective, multisite, longitudinal observational study implementing integrative care practices (ICPs) for young children with ASD. The longitudinal and observational nature of EPIGRAM has the potential to determine differences in development between migrant and nonmigrant children. Given the potential health disparities in relation to autism treatment across these two populations, the insights generated from this type of study can lay the groundwork for best practices and future research on the topic. In the current manuscript, we aim to describe baseline characteristics according to parental migration status; to examine symptom trajectories at 12-month follow-up (e.g., cognition, language, and specific and nonspecific ASD symptoms); and to assess whether migrant children showed significant differences at baseline and follow-up. Finally, we aimed to examine the same questions through parents’ own perceptions of the development of their children during the 12 months of ICPs. We hypothesized that (1) a large proportion of the children and families referred to day-care hospitals and ICPs would have a low SES and be migrants; (2) children with ASD from migrant families would have a more severe clinical presentation at baseline; and (3) children with ASD from migrant families would show a similar improvement at the 12-month follow-up to that of children from non-migrant families.

## Methods

### Study design

This study follows a prospective observational design premised on the inclusion of more than 80 children between three and six years of age diagnosed with severe ASD who were admitted to day-care hospitals to receive free integrative treatment (see the therapeutic intervention below). The ICPs had to be delivered in day-care units employing the integrative approach described in the manual. All participants had to receive between 2 and 4 half-days per week, equaling 8 to 16 hours weekly of services, and parental consent was required.

It is critical to note that the study being conducted here, on the basis of observational data, is provisional, exploratory and prospective in nature. It does not make use of a control group and is undertaken in a natural non-randomized design. The assessments of the Psycho-educational Profile Third Edition (PEP-3, French version) [31] were chosen as the primary variable (see below). All the PEP-3 assessments at M0 and M12 were filmed and assigned randomly at the end of the process, without references for the dates of administration (M0 or M12), to two psychologists working independently, who reviewed them. These professionals were not involved in the research and had training and experience in the administration of the PEP. Their blinded assessments served as external comparators for the measures carried out by the investigating centres to verify their objectiveness. The sensitivity analysis on the verbal/pre-verbal cognition and affective expression scores measured by the two independent psychologists showed that they evolved in the same way and as significantly as the scores allocated in the centres (see S1 File).

### Ethical approval

The study was allowed by the French Directorate General for Care Provision (*Direction Générale de l’Offre de Soins*) and sponsored by the National Research Program on Healthcare Performance (*Programme de Recherche sur la Performance du Système de Soins*), registered under the number PREPS1300205N. It was also registered on clinicaltrials.gov under the number NCT02154828. The study was approved by the local ethics committee (*Groupe Nantais d’Ethique dans le Domaine de la Santé*) on March 24^th^, 2014. Potential participants received verbal information about the study adapted to their level of comprehension, and their legal representatives were provided verbal and written information about the study. All parents gave written informed consent prior to inclusion.

### Participants’ recruitment

**Fig 1:**
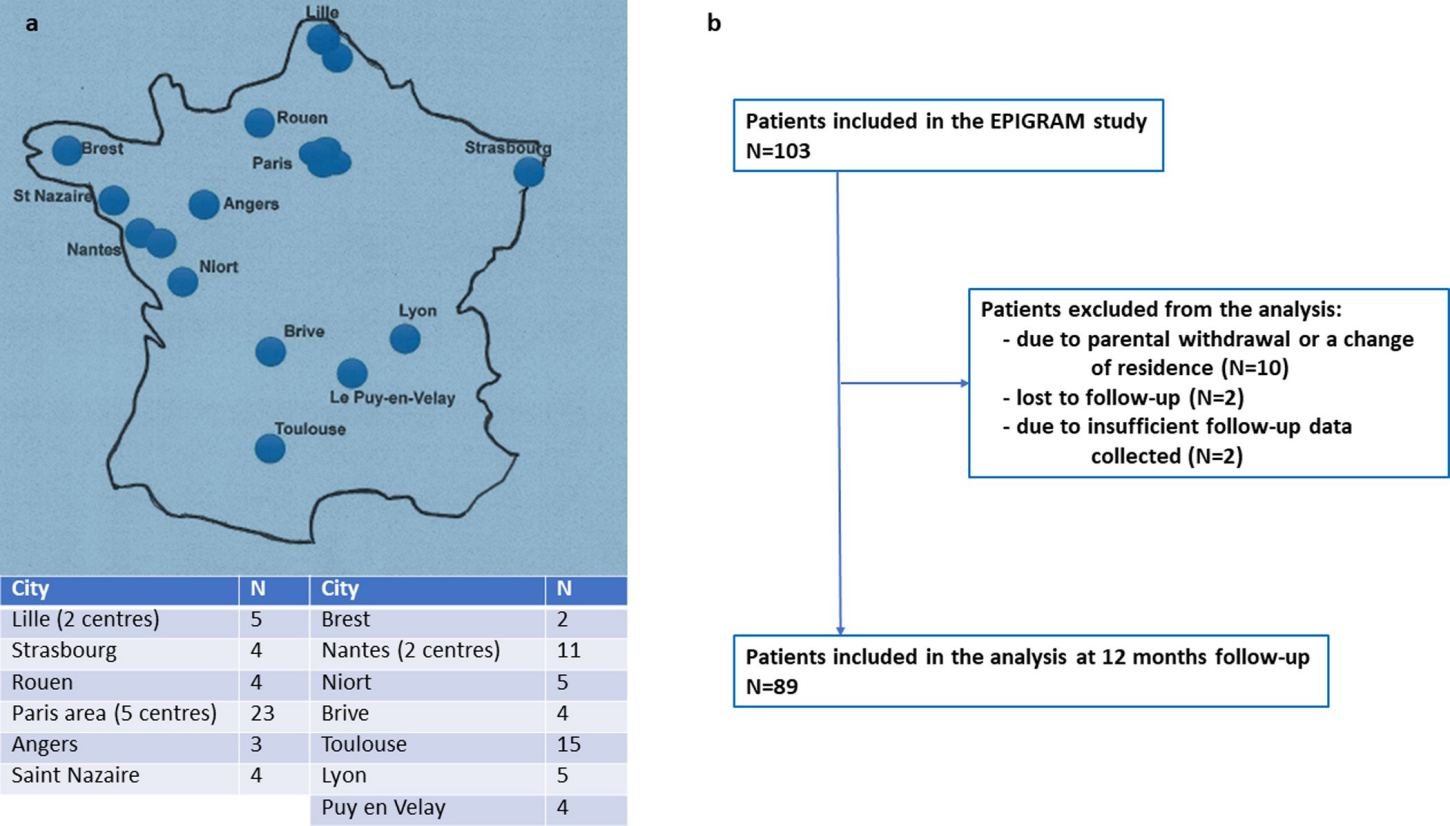
EPIGRAM study: Site location in France (1a) and diagram flow (1b).

Eighty-nine children with ASD, aged 3 to 6 years old (92% males), were recruited from 19 day-care hospitals across France (Fig 1). Participants were followed up from the first month (T0) to the twelfth month (T12) of their involvement in the study. Children were recruited from September 2014 and December 2016 in the order of their arrival to avoid biases. The study completion date was March 2018. Participants were required to not have had any type of integrative treatment. Participants were also recruited based on specific inclusion and exclusion criteria. *The inclusion criteria* include children between 3 and 6 years of age with diagnoses of F84.0 and F84.1 according to CIM-10. *The exclusion criteria* involved the presence of non-stabilized comorbidities such as epilepsy and severe somatic or sensorial impairment. Of note, there were no exclusion criteria pertaining to SES, migration, absence of insurance, or the political status of the families. The only possible biases experienced by the researchers involved the question on the stabilization of epilepsy, which was evaluated by the coordinating researchers, to determine whether the child would be included.

### Integrative care practices

#### Design of the ICP manual

ICPs are the standard of care/routinely used in many day-care hospitals in France [32]. The Integrative Practices Manual is intended to be a practical and easy-to-use manual for caregivers, doctors, psychiatrists, psychologists, nurses, educators and social workers working with children with ASD in day care and part- or full-time institutions. The manual was released in 2012 and was the starting point for EPIGRAM research. It is the result of both updated scientific data and the experience of field practitioners crossed with validated tools. In the introduction of the manual, it recalls the definition of integrative practices, their necessary coherence and liaison between professionals and partners, as well as the essential role given to the child and their family within the treatment.

The main objective of the manual is to enable caregivers to build a therapeutic, educational and pedagogical project by proceeding in stages prior to decision making and implementation. To help identify and clarify the diagnostic and therapeutic approach, the book uses areas of child development, which crosses with the impact of symptomatology and understanding of dynamics of functioning in light of theoretical and clinical sources. It enables the construction of a coordinated and coherent intervention program in conjunction with the parents and professionals involved with the child. A presentation in synthetic tables of the interventions by field as well as the essential elements of the clinical syntheses complete the book and facilitate its daily use [32].

#### Design of ICP intervention

The ICP highlights by domain: (i) a common knowledge body in child psychiatry arising from the literature. (ii) The child’s profile is objectivized and personalized in every domain through clinical observations, exchanges with the family, and other professional and standardized assessments. Accordingly, the choices can be reasonably determined and argued to determine the most suitable interventions. The approach, without being superimposable, imitates the curriculum used to assess young children in Early Start Denver Model-type programs. (iii) Therapeutic interventions have both framework and content relating to the chosen objectives.

The group structure provided by each workshop emphasized the need for social interaction and the significance of the quality of the caregiving relationship. Each group setting involved 4 to 5 participants and the duration of each session lasted between 30 and 45 minutes, depending on the child’s attention. Additionally, individual care (i.e.: psychomotricity, speech therapy, psychotherapy and sometimes individual school assistance) is available when intensive needs arise in-group settings and the child is accessible to individual rehabilitation.

The bottom-line remains to focus attention on the child’s manifestation, noting such manifestations and then understanding them to assist the child in acquiring better tools for him-self and world perception. The domain approach of analysis is multidisciplinary and utilizes clinical observation, parents’ knowledge of the child, and the corresponding functional assessments.

Overall, ICP defines the therapeutic approaches, the framework in time and space: the different workshops applied to child’s care, the individual therapies when needed, educational approaches, schooling, the continuous support of the parents, and the constant communication between the professionals and family. Examples of the interventions associated with each package or domain are shown in S1 to S9 Tables (see S1 File).

#### Domain descriptions

*The Educational (Proximal Autonomy) Domain* is a package that represents a constant demand from families. Education promotes the autonomy of the child and affords him or her a more social life. The workshop targets (i) deficits of proximal and social autonomy; (ii) frequent dysfunction in imitations that remain very selective and fragmentary and can disappear as soon as the pattern changes; and (iii) sensory peculiarities that influence the child’s autonomy. Therapeutic principles include simplifying tasks and verbal instructions; structuring space and time; building a rich toolbox of tools based on different approaches; and articulating them in interventions that are as adapted as possible to each child and carried out by practitioners who have a trusting relationship with him or her, including the parents. Depending on the child’s age and the problem involved, the ABA, ESDM, or TEACCH approach can also be used. The activities performed in this workshop relate to areas of autonomy such as nutrition, dressing/undressing and personal hygiene.

*The Sensorimotor Domain* emphasizes the association between tonic regulation and early sensory skills, body representation, and the course and action of the so-called ’corporal axis’ [33]. Sensory integration dysfunctions can affect the pathways that lead from sensation to perception. Given action-perception coupling, they also contribute to achieving a clear representation of oneself and the world. Usually, this cannot occur without an adequate affective environment. Based on individual assessment, therapeutic principles include limiting sensory stimulation that provokes irritability behaviors; establishing a framework in time and space, allowing the child to find his or her bearings and not to be surprised; keeping the framework accessible and understandable by the child; using the interest in using visual strategies, as children with ASD have better skills in this domain; and promoting motor installation (e.g., lower back support) and a smooth caregiver relationship as they facilitate interaction (e.g., being aware of the child’s attention, availability, and patterns).

*The Socialization Domain* equally manages a fundamental issue in ASD treatment. Children with autism rarely make contact and may become anxious when approached. Such children lack social interaction, do not adequately express their feelings and have a limited ability to "read" another person’s emotional state. The socialization domain targets social interaction, including a lack of initiation and reciprocity, inadequate responses, the search for immutability, autistic withdrawal, and resistance to change. Therapeutic principles include ensuring that the child is part of a temporal and emotional story; using role-playing and role-plays to help the child acquire social skills; and improving understanding of emotions and intentions through different forms of support and mediation.

*The Communication Domain* excludes the socialization dimension and emphasizes the quantitative and qualitative dimensions of verbal and nonverbal linguistic characteristics. Accordingly, the most appropriate means of communication is used in the child’s therapeutic relationship through observations and speech therapy check-ups. Speech therapy is applied where necessary. The workshop focuses on communication and varies according to the targeted aspects (e.g., syntax and speech turns), the child’s interests and the representative capacities.

*The Emotion*, *Anxiety*, *and Behavior Domain* is a dimension that individuals with ASD have claimed to represent a core experience of their dysfunction [34]. It represents the internal state from which the behavior expression in relationship with others emanates. Anxiety and emotional experiences are internal states within the child, whereas behaviors are external manifestations of them. Behaviors can be a consequence of multiple factors, which must be explored to understand the associated function and meaning for the child [35]. Hypotheses are formulated on the representations of the child’s experience and tested empirically. They include an exploration of physical pain and somatic dysfunction to distinguish the origins of such manifestations [36]. The main aim of this domain is to create a secure environment for ICPs.

*The Pedagogic (Cognition) Domain* is essential since cognitive development and knowledge acquisition are integral components of ICPs. The domain targets the development of categorization, cognitive skills, associative learning, and metaphorization. Executive functions (attention, working memory, mental flexibility, and planning) are explored because they are frequently less efficient and can be trained [37]. The domain also includes the implementation of a pedagogy adapted according to cognitive, neuropsychological, sensory, psycho-affective specificities. Inclusive schooling belongs to ICPs, and close collaboration between the care service and school is required.

*The family resources workshop* starts at the diagnostic stage during assessment and observation. Parents and children participate in various stages, and a therapeutic alliance is slowly established across the shared knowledge and connections between professionals. Families question the child’s functioning and participate in finding solutions [23]. The shared collaborations include all areas affected by the child’s disorders ranging from induced psychological suffering to social rights.

*The somatic and pharmacological assessment* supports the aforementioned domains. The knowledge of the child developed in the first phase is vital for the maintenance of well-being and the prevention and identification of somatic difficulties in the child. Accordingly, treatments adapted to certain associated disorders (e.g., seizures) are the basis of multiprofessional consultations, and prescriptions are based on benefit/risk balance.

*The Field of Intra-and Extrainstitutional Liaison* is a domain, although not necessarily a dimension, that is fundamental to the general and harmonious organization of the various treatment proposals to be included in the specific care projects. Every child meets several professionals, and continuity is maintained by a reference professional. Meetings are organized to bring together stakeholders, and according to the location, parents share information on the child’s traits for adjustment [38]. The complex issues of children under care involve several educational, health, medicosocial, associative, and financial institutions.

### Outcome measures (Table 1)

**Table 1:** Brief overview of outcome measures.

Measurement tools	Type of assessment	Timing	Domains measured	Assessment areas
ECA-R	Direct	Baseline M3, M6, M9, M12	Intensity of ASD behaviors	Relational deficiency (13 items about the inability to adapt to others in social relationships) and modulatory insufficiency (emotional and motor regulation).
PEP-3-R	Direct and by proxy	Baseline and M12	Communication, motor skills, and maladaptive behaviors	Cognitive verbal/preverbal (CVP), expressive language (EL), receptive language (RL)
				Fine motor skills (FM), gross motor skills (GM), oculomotor imitation (OMI),
				Affective expression (AE), social reciprocity (SR), characteristic motor behaviors (CMB) and characteristic verbal behaviors (CVB).
CARS	Direct	Baseline and M12	ASD severity	Social relationships, imitation, emotional responses, adaptation to changes, use of the body, objects, visual, auditory and taste responses, smell, touch, fears and anxiety, verbal and nonverbal communication, activity level, and intellectual level.
ELO	Direct	Baseline and M12	Oral language	Vocabulary, phonology and morphosyntax testing.
Family questionnaire	By proxy	Baseline and M12	30 baseline questions regarding parental concerns, and children’s social and communication development and capacity for imaginary play	M12: Same + 14 questions regarding therapeutic care, the therapeutic alliance, a global evaluation of the service, and appreciation regarding the development of the child based on initial concerns.

One of the primary outcome measures were the Psychoeducational Profile, Third Edition (PEP-3) cognitive verbal/preverbal and affective expression scores. The PEP-3 is an individualized assessment for children with suspected ASD between 2 and 7 years old. First, the test aims to measure developmental strengths, weaknesses, and learning styles to inform education programming. Second, it facilitates the autism diagnostic process by gathering information from caregivers and by providing opportunities to observe social, communication, and play behaviors. Last, it also provides developmental ages and other scores for diagnostic criteria, educational planning, and assessing changes in behavior and development over time [39]. The test is administered directly following specific guidelines, and the trained assessor derives the results from the observations made during the two parts of the test. The first one involves the performance subtests, which comprise 10 items: cognitive verbal/preverbal (CVP), expressive language (EL), receptive language (RL), fine motor skills (FM), gross motor skills (GM), oculomotor imitation (OMI), affective expression (AE), social reciprocity (SR), characteristic motor behaviors (CMB) and characteristic verbal behaviors (CVB). The second part is an assessment made by the parents or the child’s referents. The first six subtests measure developmental abilities, and the last four measure maladaptive behaviors. These ten subtests are combined into three categories: communication (CVP, EL, RL), motor skills (FM, GM, OMI), and maladaptive behaviors (AE, SR, CMB, CVB). The results are obtained in the form of raw scores, and the first six items of the communication and motor skills categories are converted into developmental ages calculated on a sample of children whose development is neurotypical; the ten items of the three categories are calculated in rank percentiles on a sample of children with ASD and transformed into developmental/adaptive levels for all. The main criteria of the results from the PEP-3 were carried out in percentile rank to integrate the “natural” evolution of the child over the year of inclusion and because it gives scores as a function of children with ASD.

The secondary outcome measures included the *Echelle des Comportements Autistiques* (Autistic Behavior Rating Scale—ECA-R), the Khomsi *Evaluation du Langage Oral* (Oral language assessment—ELO), the Childhood Autism Rating Scale (CARS), and a family questionnaire. All tests were measured at baseline and T12, with the exception of ECA-R, which was also measured at M3, M6 and M9. The ELO is a non-specific tool for ASD and is used quite commonly in child psychiatry from Pre-K to 5^th^ grade. A central place is given to understanding the heterogeneity of language development, which can be grasped with this tool. Three axes are explored: vocabulary, phonology and morpho-syntax. This instrument allowed us to have a more focused analysis of the development of the child’s oral language.

The CARS is a behavioral rating scale used for assessing the presence and severity of symptoms of ASDs. The test is made up of 14 assessment areas: social relationships, imitation, emotional responses, adaptation to changes, use of the body, objects, visual, auditory and taste responses, smell, touch, fears and anxiety, verbal and non-verbal communication, activity level, and intellectual level. A 15th scale measures the examiner’s overall impression of the severity of the autism. Each subscale is rated from 1 to 4, with 4 being severely abnormal. The total points vary between 15 and 60, and the cut-off score is 30. From 30 to 36.5, we are in the register of mild-moderate autism, and from 37 and up, we are in the register of severe autism.

The ECA-R allows exploring the intensity of ASD behaviors in different domains through the use of a 29-item scale. The frequency of these behaviors is rated from 0 (never) to 4 (always), which allows a total score to be produced. It is useful for obtaining an analysis of the evolution of the profile evaluated. It is possible to follow the evolution of criteria gathered under two scores: relational deficiency (13 items), which explains the inability of the child to be in an adapted relationship with others, and modulatory insufficiency (3 items), which testifies to the weak capacity for emotional regulation and motor behavior. We chose it to reflect the development of the child during the stages of the research. It is a tool used by child psychiatry caregivers that, like the CARS, brings together professionals around an instrument objectifying the changes according to the different dimensions and allowing an adjustment of the care plan.

Finally, the family questionnaire was elaborated based on the Tavistock Clinic and Portman NHS Trust (London), which allows taking into consideration parental observations concerning their perception of their child’s ASD symptoms and manifestations of the evolution, and input regarding the therapeutic alliance. The questionnaire contains 30 questions before inclusion and 44 at the end of the intervention [40]. The questionnaire is divided into sections: the first, at T0, investigates the principal parental concerns of their child; the other three, which are identical at T0 and T12, investigate social and communication development as well as the capacity for imaginary play. Regarding the questions, the answers are ranked from “not at all” (0) to “yes, usually” (5). The final questionnaire makes use of the same sections while it also incorporates information regarding the therapeutic care, the therapeutic alliance with the different professionals, and a global evaluation of the service, and the levels of appreciation regarding the evolution of the child based on the initial concerns. The evaluations make use of a scale from 0 to 4, from “aggravation” to “satisfying evolution”. The professional questionnaire is identical to the parents’ questionnaire and was only adapted linguistically.

### Statistics

Analyses were performed using R software 4.0.2 (https://www.r-project.org/) by two-tailed tests with a level of significance fixed at 5%. We first described the population study at baseline. The quantitative variable distributions were summarized using the mean and standard deviation. Alternatively, we used the median and Q1-Q3 quartiles, depending on the shape of the distribution. The qualitative variable distributions were summarized using the number and percentage of occurrences. Then, the total sample was divided into two groups based on the migration status. We compared those two groups across socio-demographics and clinical characteristics at baseline. Depending on the validity of the assumptions, the quantitative variables were tested either using a Welch’s t-test or a Wilcoxon rank sum test. Similarly, qualitative variables were tested either using a chi-squared test without continuity correction or a Fisher’s exact test.

Then, we performed longitudinal analyses. Each outcome variable was studied separately. To assess the progress, the evolutions of the primary and secondary variables were estimated using linear mixed effect modelling (lme4 and lmerTest packages). The model formula was “Score ~ Migration + Time + (1|Center/Subject)” with migration set as a between-subject variable, time set as a within-subject variable and subject and center set as random intercepts, with subject nested within the center. The model assumptions were checked visually using regression plots. One model was run per variable score, and we reported the time effect for each score. Finally, to assess the difference in progress between the migrant and non-migrant groups, we used linear mixed effect modelling, adding the eventual statistical interaction between the group status and time (migration*time). The model formula was set as “Score ~ Migration + Time + Migration*Time + (1|Center/Subject).” The model assumptions were checked visually using regression plots, and one model was run per score. For each outcome variable and model, the estimated time effect, migration*time interaction effect, the corresponding profile confidence interval (95% CI) and the p-value were reported.

Finally, we performed a power analysis with G. Power 3.1.9.2 to find the minimal effect size, which was able to detect with enough power and a limited type I error. The results show that for a linear multiple regression, N = 89 subjects is sufficient to detect a f^2^of 0.09 (small-moderate effect size) with 80% power and 5% type I error. Analyses included repeated measurements of participants, so this should be viewed as a worst-case scenario. The effective sample size is expected to be larger, and as a consequence, the minimal effect size we can detect is expected to be less than 0.09.

## Results

### Overall description of the sample

In total, 89 children (92% of males, mean age = 4 (standard deviation (SD) = 0.8) years) were included in the analysis from an initial sample of 103 included patients, presenting on average a severe type of autism based on CARS (mean = 44; SD = 6.51) and moderate autism based on inappropriate behaviors from PEP-3 (mean = 30; SD = 29.89). Fourteen patients were not included because of early drop-out: (N = 10) due to parental withdrawal or a change of residence; (N = 2) lost to follow-up; and (N = 2) due to insufficient follow-up data collected (Fig 1). All participants were diagnosed before the age of 3 years and 6 months, and most of them suffered from severe language delays. Thirty percent of the families involved in this study have a low socio-economic status (SES). SES difficulties were identified when one of the parents did not have salary income and the family received some form of financial aid. Fifty six% are first-generation migrants, and 77% of the children have special needs assistance during school. As expected, the proportion of children from immigrant families was much higher than that measured in the most recent epidemiologic French data on autism (see Discussion below). Table 2 summarizes the socio-demographic and clinical characteristics of the participants. The rather balanced distribution of migrants (N = 50, 56.2%) vs. non-migrants (N = 39, 43.8%) allowed further analyses.

**Table 2:** Characteristics of the children included in the EPIGRAM study at baseline (N = 89).

	All (N = 89)	Nonmigrant 39 (43.8%)	Immigrant 50 (56.2%)	Test	p-value
***Socio-demographic Variables***					
Gender Male: n(%)	82 (92%)	35(89.7%)	47 (94%)	F	0.695
Age: median [q1-q3]	4[3–5]	4[3–4]	4 [3.25–5]	W	0.076
Separation from extended family* (yes): n(%)	40 (45%)	6 (15.4%)	34 (68%)	Chi2	<0.001
Serious illness of a parent (yes): n(%)	21 (24%)	10 (25.6%)	11 (22%)	Chi2	0.688
Socioeconomic difficulties (yes): n(%)	27 (30%)	7 (17.9%)	20 (40%)	Chi2	0.025
Separated parents (yes): n(%)	21 (24%)	9 (23.1%)	2 (24%)	Chi2	0.919
***Clinical characteristics***					
Typical (F84-0)/ Atypical Autism (F84-1)	65 (73%)/ 24 (27%)	26(66.7%) / 13(33.3%)	39(78%) / 11(22%)	Chi2	0.232
Absence of language (yes): n(%)	40 (45%)	12(30.8%)	28(56%)	Chi2	0.018
Eating disturbances (yes): n(%)	69 (78%)	30(76.9%)	39(78%)	Chi2	0.904
Sleeping disturbances (yes): n(%)	52 (58%)	25(64.1%)	27(54%)	Chi2	0.337
***Child’s history***					
Acute illness (yes): n(%)	23 (26)	12(30.8%)	11(22%)	Chi2	0.348
Pregnancy difficulties (yes): n(%)	29 (33)	14(35.9%)	15(30%)	Chi2	0.556
Childbirth difficulties: median [q1-q3]	0[0–1]	0[0–1]	0[0–1]	W	0.742
***School enrolment at inclusion***					
Schooling (yes): n(%)	78 (88%)	35(89.7%)	43(86%)	F	0.749
With a special needs’ aid (yes): n(%)	60 (67%)	25(64.1%)	35(70%)	Chi2	0.556
Special needs class(yes): n(%)	4 (5)	1 (2.9%)	3 (7%)	F	0.623
Delayed in comparison to the grade’s level	0[-0.5–0]	0[0–0]	0[-0.75–0]	W	0.896
***Clinical variables at baseline***					
CARS	43.7 (6.5)	41.17(5.58)	45.77(6.51)	t	0.001
PEP-3-R verbal/preverbal cognition: median [q1-q3]	36[14–64]	52[26–76]	24.5[9.75–45.5]	W	0.001
PEP-3-R expressive language: median [q1-q3]	31[12–52]	43[28–68]	15[9–33]	W	<0.001
PEP-3-R receptive language: median [q1-q3]	30[11–63]	53[15.5–80.5]	15[5.5–44.25]	W	0.001
PEP-3-R fine motor skills: median [q1-q3]	32[24–62]	57[31–75.5]	29[11.25–43]	W	<0.001
PEP-3 gross motor skills: median [q1-q3]	29[14–48]	46[26.5–67]	22[5–30]	W	<0.001
PEP-3-R oculo-motor imitation: median [q1-q3]	28[13–59]	50[22–74]	22[9.5–37.25]	W	0.001
PEP-3-R affective expression: median [q1-q3]	45[25–68]	58[35–81]	38[25–58]	W	0.014
PEP-3-R social reciprocity: median [q1-q3]	41[17–59]	59[17–79.5]	28[12–50]	W	0.005
PEP-3-R motor behaviors characteristics: median [q1-q3]	30[12–58]	58[23.5–76]	25.5[6.5–43.5]	W	0.002
PEP-3-R verbal behaviors characteristics: median [q1-q3]	15[4–41]	18[7.5–72]	8[4–29.75]	W	0.016
CAT communication: median [q1-q3]	23[11–55]	46[18–73]	14[6.25–31]	W	0.001
CAT motor skills: median [q1-q3]	29[15–57]	43[28–78]	17.5[8.5–32]	W	< 0.001
CAT inappropriate behaviors: median [q1-q3]	11[6–50]	41[9–77]	8[5.25–34.5]	W	0.002
ECA-R global: mean (SD)	62.45 (17.68)	59.05(15.67)	65.1(18.83)	t	0.102
ECA-R relationship impairment: median [q1-q3]	32[25–40]	30[21–35]	35[28.25–43]	W	0.007
ECA-R modulatory insufficient: median [q1-q3]	5[4–8]	6[3.5–8]	5[4–7]	W	0.389

*Separation from extended family means that the children’s parents are first generation migrants in France and their respective families remain in the homeland.

CARS: Child Autism Rating Scale; PEP-3-R: Psychoeducational Profile 3^rd^ edition Revised, CAT: Category; ECAR: Echelle Comportements Autistiques Révisée

### Sociodemographic and clinical characteristics according to migrant vs. non-migrant family status at baseline

In this section, we analysed the differences between children from migrant and non-migrant families at baseline with regard to socio-demographics, history, and clinical characteristics. The distribution, mean (SD) or median [quartile 1-quartile 3] of the relevant variables are shown in Table 2 with statistical comparisons. In terms of socio-demographics, we found no significant difference for age, gender distribution, schooling, or parental separation. However, migrant families had significantly more socioeconomic difficulties and were more often lacking relatives.

Regarding clinical characteristics, we found no difference in the CIM-10 diagnostic distribution, parental medical history, pregnancy or delivery history, previous eating, sleep or medical conditions. However, children with ASD from migrant families were more severe in most clinical variables exploring development, ASD symptomatology and atypical behaviors. We found a higher proportion of children from migrant families without language. The participants from migrant families had more severe autism (CARS: mean = 45.77 (SD = 6.51) vs. 41.17 (5.58), t-test, p = 0.001) and more severe cognitive impairment (PEP3-R verbal/preverbal cognition: median = 24.5 [q1-q3: 9.75–45.5] vs. 52 [q1-q3: 26–76], Wilcoxon rank sum test, p = 0.001). In addition, we observed significant differences across the three dimensions of the PEP-3 (motricity, communication and inappropriate behaviors) in children from migrant families, exhibiting more severe scores than children from non-migrant families (Table 2). Finally, on average, migrant children had 3.4 half days of care per week. In contrast, non-migrant children had on average 3.1 half days of intervention per week.

### Developmental outcomes at 12-month follow-up

**Fig 2:**
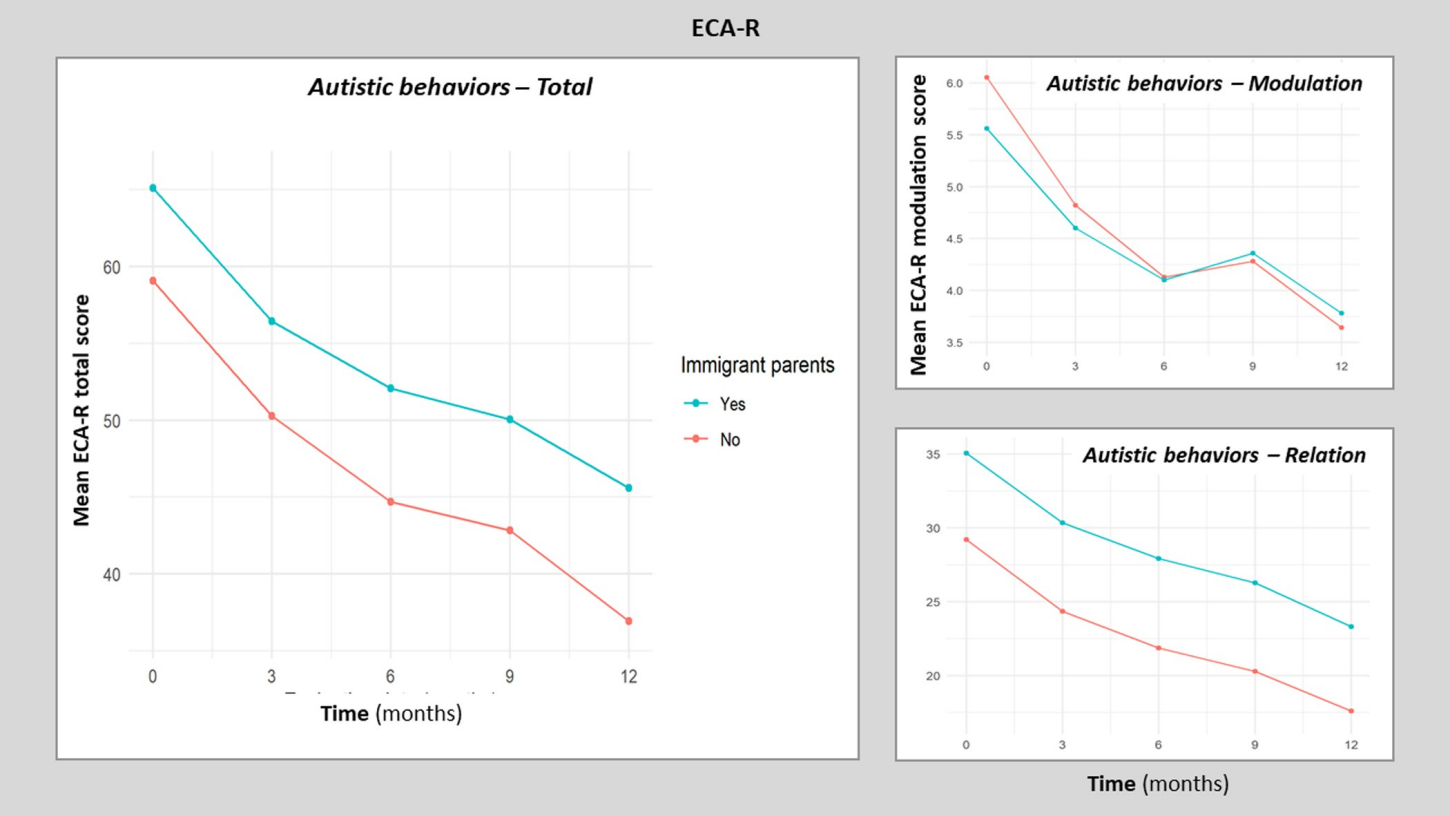
Evolution of ECA-R scores during the 12-month follow-up.

In this section, we explored the course of clinical characteristics over time for the whole sample using linear mixed models without (migration*time) interaction. The data are summarized in Table 3 (and S10 Table for ELO, see S1 File). We found that all clinical characteristics, including the two primary variable outcomes (PEP3-R verbal/preverbal cognition and PEP3-R affective expression), improved during the 12 months of the EPIGRAM study, showing the overall positive impact of the integrative intervention. We can observe that the ECA-R total score significantly decreased by an average of 1.61 points/month (95% CI [-1.79; -1.42], p<0.001). The ECA-R insufficiency mode and ECA-R social deficiency significantly decreased as well, which are positive therapeutic outcomes. Fig 2 details the course by trimester of the ECA-R scores. It shows that at baseline, the means are different for children with migrant parents and those with non-migrant parents. However, the decrease over time appears parallel in children with non-migrant parents and children with migrant parents.

**Table 3:** Evolution throughout the 12 months for children included in the EPIGRAM study at baseline (N = 89) (time effect model without interaction).

	Estimate	95% CI	Std. Error	Pr(>|t|)
PEP-3-R verbal/preverbal cognition	0.62	[0.28;0.95]	0.17	0.001
PEP-3-R affective expression	0.89	[0.51;1.28]	0.2	< 0.001
PEP-3-R expressive language	0.56	[0.24;0.87]	0.16	0.001
PEP-3-R receptive language	0.79	[0.49;1.1]	0.16	< 0.001
PEP-3-R fine motor skills	0.62	[0.25;0.99]	0.19	0.001
PEP-3-R gross motor skills	0.4	[0;0.81]	0.2	0.051
PEP-3-R oculo-motor imitation	0.61	[0.24;0.99]	0.19	0.002
PEP-3-R social reciprocity	1.19	[0.88;1.5]	0.16	< 0.001
PEP-3-R motor behaviors characteristics	0.83	[0.49;1.17]	0.17	< 0.001
PEP-3-R verbal behaviors characteristics	1.28	[0.83;1.73]	0.23	< 0.001
CAT communication	0.61	[0.38;0.83]	0.11	< 0.001
CAT motor skills	0.58	[0.29;0.88]	0.15	< 0.001
CAT inappropriate behaviors	1.1	[0.77;1.43]	0.17	< 0.001
CARS	-0.55	[-0.65;-0.45]	0.05	< 0.001
ECA-R global	-1.61	[-1.79;-1.42]	0.09	< 0.001
ECA-R modulatory insufficient	-0.15	[-0.18;-0.12]	0.02	< 0.001
ECA-R relationship impairment	-0.91	[-1.01;-0.81]	0.05	< 0.001

* CARS: Child Autism Rating Scale; PEP-3-R: Psychoeducational Profile 3^rd^ edition Revised, CAT: Category, ECAR: Echelle Comportements Autistiques Révisée

Regarding the PEP-3 motor dimension, we observed a general increase across the three variables, except for gross motor, which was nearly significant (p = 0.051). For instance, fine motor function increased by 0.62 percentiles per month (95% CI [0.25;0.99], p = 0.001). The communication dimension also showed a general increase in the three variables (cognitive verbal/preverbal, expressive language and receptive language). Notably, receptive language increased by 0.79 percentiles per month (95% CI [0.49;1.1], p<0.001). Last, we can also observe a general increase in the four variables from the maladaptive behavior dimension (affective expression, social reciprocity, characteristic motor behavior, and verbal behavior characteristics). Notably, PEP-3-R-CVB had the largest effect size, with a significant increase of 1.28 percentiles per month (95% CI [0.83;1.73], p<0.001).

**Fig 3:**
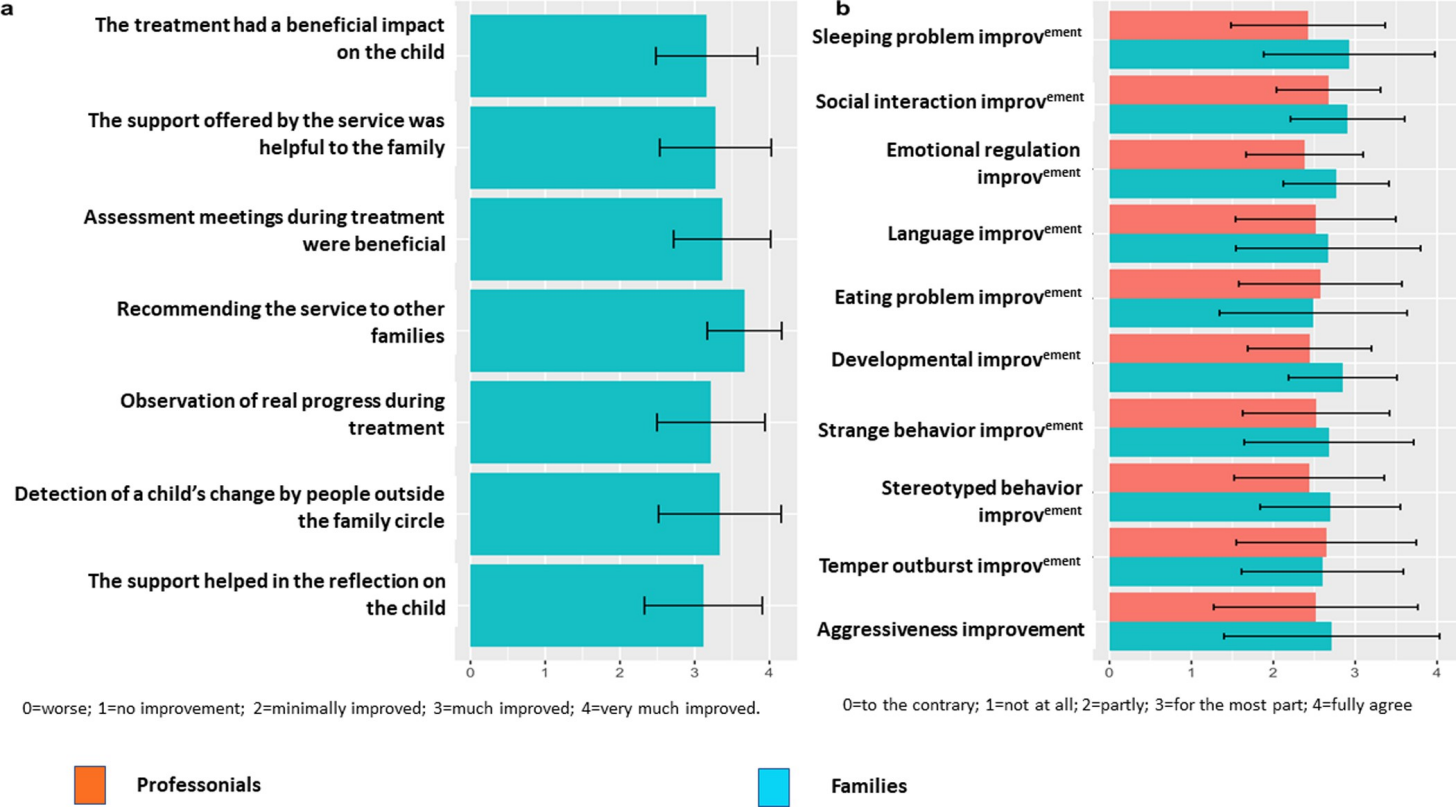
Families’ subjective views of their children’s improvement (3a) and of the services provided (3b).

Fig 3 summarizes how families perceived their children’s changes over time (Fig 3A) and their view of the integrative treatment (Fig 3B). Interestingly, families and professionals had similar perceptions of improvement, regardless of the clinical dimension. Mostly, they found that the improvement ranged between minimally improved and much improved, showing the heterogeneity of children’s improvements over time. Additionally, families had a positive view of the integrative treatment and recommended it for other families (Fig 3B).

### Do children from migrant families develop differently?

In this section, we analyze the impact of migration on children’s progress using linear mixed effect models with (migrant*time) interaction. The results are summarized in Table 4. Overall, despite the most severe clinical scores at baseline in the migrant group, the most clinical characteristics did not show any significant interaction, meaning that the range of improvement was not significantly different between groups (see Fig 2 for ECAR scores over time). However, we found a significant interaction for two variables: verbal behavior characteristics (PEP-3-R-CVB) and the inappropriate behavior category (PEP-3-R-CATCI). The PEP-3-R-CATCI score increased by an average of 1.56 points per month in the nonmigrant group (time effect of the model with interaction; p<0.001). The effect of the interaction between migration and time was -0.83 percentile/month for children whose parents were migrants vs. for children whose parents were nonmigrants (95% CI [-1.47; -0.18], p = 0.014). This means that migrants progressed on average less with time than nonmigrants. Similarly, the PEP3-R-CVB score increased by an average of 2.03 points per month in the nonmigrant group (p<0.001). The effect of the interaction between migration and time was -1.32 percentile points/month for children whose parents were migrants vs. for children whose parents were nonmigrants (95% CI [-2.19;-0.46], p = 0.003). This means that migrants progressed on average less with time than nonmigrants.

**Table 4:** Evolution throughout the 12 months for all children included in the EPIGRAM study according to their immigrant status (time*migration effect model with interaction).

	Estimate	95% CI	Std. Error	Pr(>|t|)
PEP-3-R verbal/preverbal cognition	0.01	[-0.66;0.69]	0.35	0.967
PEP-3-R affective expression	-0.45	[-1.22;0.31]	0.39	0.25
PEP-3-R expressive language	-0.12	[-0.75;0.52]	0.32	0.714
PEP-3-R receptive language	0.14	[-0.48;0.76]	0.31	0.658
PEP-3-R fine motor skills	0.65	[-0.08;1.38]	0.37	0.084
PEP-3-R gross motor skills	0.22	[-0.59;1.03]	0.41	0.602
PEP-3-R oculo-motor imitation	0.3	[-0.46;1.05]	0.39	0.441
PEP-3-R social reciprocity	-0.05	[-0.68;0.58]	0.32	0.871
PEP-3-R characteristic motor behaviors	-0.06	[-0.74;0.62]	0.35	0.861
PEP-3-R characteristic verbal behaviors	-1.32	[-2.19;-0.46]	0.44	0.003
CAT communication	-0.35	[-0.8;0.1]	0.23	0.133
CAT motor skills	0.33	[-0.26;0.93]	0.3	0.279
CAT inappropriate behaviors	-0.83	[-1.47;-0.18]	0.33	0.014
CARS	-0.11	[-0.32;0.09]	0.1	0.276
ECA-R global	0.21	[-0.16;0.59]	0.19	0.27
ECA-R modulatory insufficient	0.05	[-0.01;0.12]	0.03	0.116
ECA-R relationship impairment	-0.01	[-0.21;0.19]	0.1	0.927

**Fig 4:**
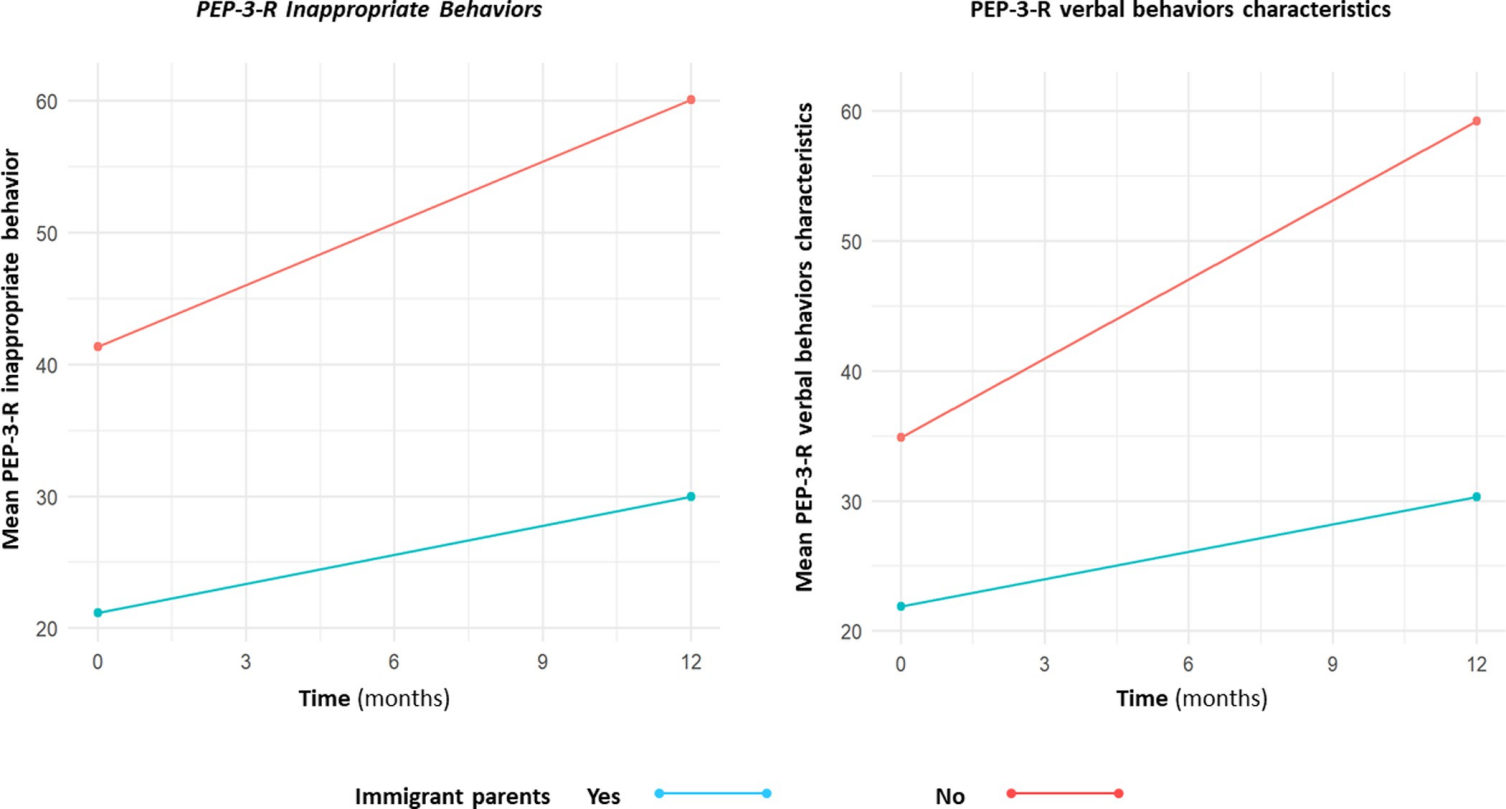
Evolution of inappropriate PEP-3-R behaviors and PEP-3-R verbal behaviors characteristic of a negative interaction between course over time and family migration status during the 12-month follow-up.

The impact of migration on the PEP-3-R-CVB and PEP-3-R-CATCI scores showed a negative trend over time (Fig 4). As a consequence, there is a significant difference in the time effect on average between the two groups. Therefore, it is conclusive that the migration effects are adverse for children with migrant parents under the verbal behavior characteristics and inappropriate behavior dimensions. Furthermore, we observed that PEP-3-R-CVB and PEP-3-R-CATCI, two domains that characterize ASD symptomology, were significantly more present in the migrant group, as if they had more autistic elements than the nonmigrant group, despite experiencing the same intervention and early diagnosis.

## Discussion

Over the past decades, different treatments, such as behavioral, developmental, and cognitive-behavioral interventions, have been developed to help children with ASD [35]. Here, we examined the impact of an integrative approach on children between 3–6 years of age with ASD from migrant and non-migrant families. The study used a non-randomized multi-site longitudinal observational approach and compared the differences between nonmigrant children with ASD and children with ASD from first-generation immigrant parents and lower social-economic backgrounds, within the French healthcare system. We monitored 89 out of the initial 103 children who were included in the study. This means that only 86% of the initial sample participated in the study. This attrition rate is low, which shows that the families were positively involved in the study. As expected, 30% of the children included in the study were from a low social-economic background, while 56% were migrants. The sociodemographic and clinical characteristics of the sample were in line with the most recent French epidemiological study carried out by Delobel-Ayoub et al. [4], whose results showed that the prevalence of ASD was higher in regions with high-level deprivation, a large number of unemployed adults, people with no higher education, migrants and single parents. However, Delobel-Ayoub et al. found a prevalence of migrants of 43% in all ASD (36% in children with ASD and without ID; 44% in children with ASD and ID). The 56% prevalence of migrants found in our sample is higher and supports the idea that ICPs in the French healthcare context address children of migrants with a low socio-economic background [40].

Comprehensive treatment for autism is complex, expensive and often offered in private settings. Therefore, migrant families with a low SES might not have access to such care for their children because of their budgetary constraints (e.g., [41, 42]). Moreover, care centers for children with ASD might not be available within their locality. In this case, a lack of transportation is a problem based on the distance and costs involved. The financial challenges are further compounded by fragmented services within the care system of children with ASD. In most cases, the care system has varied specialists in different areas, leading to serious access challenges for people who have no money and limited transport systems [43]. Social issues are also part of a larger perspective to examine the impediments to care for children with ASD among the migrant population. Stigma and discrimination are common elements in the community that culminate in shame and guilt. Therefore, the parents of children with ASD might feel embarrassed of their situation, prompting the withdrawal of the child from the public eye [6]. A lack of social support also means that parents might not have the right structure to deal with their children with ASD. If the community is not well-structured to embrace such cases of disabilities, access to care might be a challenge [6]. Based on the nature of migrant communities, their beliefs might be different from those of the health service providers within the host countries. Parents might not understand their roles in the care process if their value system is different from that of service providers. They might also be advised to implement home therapies despite not having the desired levels of cooperation [43]. In the French healthcare system, treatment is accessible and free for everyone, despite migrant status or SES, since the Healthcare Universal Protection that started in 1999 [44]. Children from such backgrounds can access treatment and attend regular or special needs classrooms. In a prospective follow-up study performed by [45], participants were recruited from 15 centres that focused on diagnosing and following up with children with ASD. The results showed that 88% of the participants attended schools, while those with severe adaptive and cognitive deficits were unlikely to attend. This means that despite full free access to care, parents with more severe children have more difficulties accessing school inclusion despite unanimous recommendations [23].

The results of the current study show that the children of migrants progress less quickly in terms of the PEP-3-R characteristic verbal behaviors and inappropriate behavior dimensions although they received more hours of intervention. Migrant children also show more severe ASD symptomatology and therefore the intensity of the intervention in terms of the number of hours is higher than the non-migrant group. It is likely that this higher severity is secondary to a mixture of contributors, including (i) a higher risk per se of more severe ASD and/or ID in children from immigrant families (e.g., [4]); (ii) more frequent socio-economic limitations (e.g., [41]; and (iii) cultural factors, as they are in a new environment with a different culture, making it hard for them to communicate [6] and use the local healthcare system [43]. Cultural perceptions may also play a role in the severity of autism in children. For example, Pondé and Rousseau [46] noted that immigrant parents recognize the symptoms of ASD early but perceive them as developmental delay or communication problems. Regarding lesser progress in terms of the verbal and preverbal behavior and inappropriate behaviors’ dimensions, we also believe it is a consequence of a mixture of contributors including: (i) a higher rate of ID [4]; (ii) a higher rate of social deprivation by a lack of access to school and exposure to language [17]; (iii) lower rates of early intervention [6]. The association between immigrant families and inappropriate behaviors seems to be life-long, as it has also been found in adolescents and young adults with autism showing challenging behaviors (e.g., 62% in [47]).

Regarding the excellent attrition rate, we briefly propose some speculative remarks regarding the French healthcare system. The participants in this study were mostly from families with a lower SES who were first-generation migrants. Migrant parents of children with autism belonging to a low SES have the disadvantage of navigating through the complexities of autism treatment in their host country. Autism treatment requires the assistance of different specialists in varied areas, and this could be inconveniencing and cumbersome for parents within a low SES and a migrant status [43]. However, the integrative approach described here combined with free access to care, even for immigrant families with no administrative status, provides relief to these parents. The ICP centers have an impeccable interconnectedness of different specialists that allow parents to seek the best advice and help for their children and therefore access comprehensive treatment within a single location. Parents also become active recipients of the information regarding the treatment journey of their children. Since the input of parents is valued, they are able to navigate the complexities together with the specialists without feeling overwhelmed.

### Limitations

Ultimately, considering that this study is a non-randomized and exploratory observational study, it has evident limitations in relation to potential generalizations and extrapolations. Despite the lack of blind assessments, we conducted a sensitivity analysis using PEP-3-R video-recorded clinical sessions blindly rated by independent experts. We found that linear mixed models based on blind assessment evolved in the same way as those based on clinical assessment for the two primary outcome variables: PEP-3-R-CVP and PEP-3-R-AE scores (see S1 File). We also found that parents’ and clinicians’ subjective views at 12 months were similar (Fig 3). The PEP scale was developed by the proponents of the TEACH program and may not be ideal in the context of other interventions, such as ICPs, or with migrant population. Our sample (N = 89) size was sufficient for our analysis but still limited to detecting discrete relevant effects. Due to the concentration of immigrant families in large cities in most Northern developed countries, there was an association between study cites and immigrant status. To ensure that the results presented on migration are not due to confounding variables, we performed sensitivity analyses while taking into account the following variables: socio-economic difficulties, age, autism severity, intensity of intervention, and center. We found that the linear mixed models applied in secondary analyses produced the same results (see S1 File). Finally, we could not control whether all centers could offer services in native languages and if this barrier affected our results. Nevertheless, given the dearth of research on ICPs despite their very common use [28] and the potential disparities in care faced by low-income and immigrant families, this research project contributes to laying the groundwork for a longer-term research agenda meant to explore this link in a detailed manner.

## Conclusion

We found that ICPs implemented in day units improved, both in terms of clinical variables and parents’ subjective views, children with severe and complex ASD between three and six years old. Children with ASD differ depending on their migrant or non-migrant background. Given the lack of randomization or control group, this trend may be attributed to ICPs or any naturally occurring event during that period. In most cases, the children with ASD from migrant populations show a higher severity at baseline on the autism spectrum and on the developmental dimension. They also have problems in communication and inappropriate behaviors. Therefore, they require much time and care to achieve the desired levels of communication and social behavior. The greatest advantage is that the ICPs in daycare units have been structured well to deal with such cases regardless of the challenges faced. Furthermore, the children attain adequate care without the parents bearing any financial burden. Overall, children with migrant parents exhibit more severe ASD characteristics at baseline and at the end of the intervention but do show an overall positive progression in a similar manner across time in comparison to the non-migrant group. However, migration is associated not only the baseline severity but also progress over time of inappropriate behaviors in children with ASD. We believe that there is an urgent need to target the migrant population with specific research and understand the avenues that carry such higher severity.

## Supporting information

S1 File (PDF)

S2 File (PDF)

S3 File (DOCX)

S1 Checklist CONSORT 2010 checklist of information to include when reporting a randomised trial*. (DOC)

## Abbreviations

ASD: autism spectrum disorder
ICPs: integrative care practices
PEP-3-R: psychoeducational profile-3-R
CARS: children autism rating scale
