# Supplementary material for: Migration is associated with baseline severity and progress over time in autism spectrum disorder: Evidence from a French prospective longitudinal study
Source: PLoS One. 2022 Oct 6;17(10):e0272693. doi: 10.1371/journal.pone.0272693 (PMC9536617; doi:10.1371/journal.pone.0272693)
Supplement: S1 File — (PDF) [file pone.0272693.s001.pdf]

Protocole AUTISME

« Clinical evaluation of integrative practices in infant and child care units for children with typical or atypical autism »

Investigator Co-ordinator or person who directs and supervises the conduct of the research: Dr Nicole Garret-Gloanec
Hospital Practitioner, Head of Department,
Department of Child-Psychiatry II, Saint Jacques Hospital - CHU de Nantes
Rue de Saint-Jacques, 44093 Nantes cedex 1, FranceTel : 02 28 08 84 90
Mail : nicole.garret@wanadoo.fr

Méthodologist:
Jean-Benoit HARDOUIN
EA 4275 « Biostatistics, Pharmacoepidemiology and Subjective Health Measures»
Université de Nantes, Faculté de Médecine, bureau 221
1, rue Gaston Veil – BP 53508, 44035 Nantes cedex 1
Tel : 02 40 41 28 29
Fax : 02 40 41 29 96
Mail : jean-benoit.hardouin@univ-nantes.fr

Instituion responsible for the research:
CHU de Nantes
Contact : Anne Omnès
Département promotion, Direction de la Recherche
5, allée de l'île Gloriette
44 093 Nantes cedex 01 (FRANCE)
Contact : Tel : 02 53 48 28 35
Fax : 02 53 48 28 36

Partner (s) :
Jean-Michel THURIN, Psychiatre, Inserm U669

Tel : 02 53 48 28 35
Fax : 02 53 48 28 36

Fédération Française de Psychiatrie

Hôpital Sainte Anne – Paris
Tel : **01 48 04 73 41 –** Fax : **01 48 04 73 15**

SUMMARY

**Title of the study**	Clinical evaluation of integrative practices in infant and child care units for children with typical or atypical autism
**Keywords**	Integrative practices, clinical assessment, case studies, autism, child psychiatry, units of care
**Head of research**	**CHU DE NANTES**
**Investigator coordinator**	Doctor Nicole Garret-Gloanec
**Scientific responsible person**	Professor Olivier Bonnot
**Investigators co - coordonnators**	Docteurs Fabienne Roos Weil et Maria Squillante
**Number of centers planned**	National study: 17 centers planned
**Type of study**	Non-interventional research
**Planning of the study**	❖ Total duration: 40 months<br>❖ Recruitment period: 36 months<br>❖ Length of follow-up per patient: 12 months
**Study design**	❖ Multicentric<br>❖ Observational<br>❖ In a natural situation<br>❖ Not Controlled<br>❖ Open<br>❖ Foresight
**Objectives of the study**	Primary Objective: The evaluation of care practices, based on the evolution over one year, of children aged 3 to 6 years, with a diagnosis of typical or atypical autism (F 84-0 and F 84-1), treated in a care unit practicing integrative approaches (Centre Accueil Thérapeutique à Temps Partel CATTP, Day hospitals).<br><br>Secondary objectives:<br>- To show the effectiveness of integrative practices by evaluating the overall evolution of the children assessed according to validated and selected clinical criteria and evaluation tools.<br><br>- Evaluation by the families :<br>- the child's condition and its evolution<br>- the quality of the relationship established by the team with the family.<br><br>- Standardize the use of evaluation scales.
**Projected number of cases**	80 patients

**Schedule of the different visits and examinations**	- M0 visit: collection of parental consent, inclusion, assessment of developmental dimensions using standardized tools, structured clinical observation, family questionnaire n°1, professional questionnaire n°1.<br>- Supervision by a research assistant of the assessments made at M0 and M12.<br>- Visits M3, M6, M9: autistic behavior assessment scale and possible contextual events.<br>- Visit M12: end-of-study visit, evaluation of developmental dimensions using standardized tools, structured clinical observation, family questionnaire n°2, professional questionnaire n°2.
**Main selection criteria, inclusion, non-inclusion and exclusion criteria**	General Inclusion Criteria :<br>- Children 3 to 6 years of age with a diagnosis of F84.0 and F84.1 according to ICD-10 criteria. The diagnosis must have been made according to the criteria established by the Diagnostic Guidelines (FFP/HAS 2005).<br>- In care units meeting defined criteria for integrative practices.<br>- Children receiving a volume of intervention hours between two and four half-days per week.<br>- Collection of parental consent.<br><br>Criteria for non-inclusion: presence of co-morbidity such as epilepsy, severe organic, somatic and sensory impairment.
**Main outcome**	Developmental assessment in the domain of verbal and preverbal cognition and maladaptive behaviors in the domain of affective expressions of children between time M0 (month of inclusion) and M12 (12th month of follow-up) according to the PEP 3 scale.
**Secondary outcome**	- Global evolution of the child assessed by area of development (language and communication, sensorimotor, social interactions, behaviours, cognitive domain, anxieties and emotions) using the ECA-R, CARS, and speech and language therapy (ELO) and psychomotor (Brunet-Lézine) scales at M0 and M12.<br><br>- Questionnaire-assessed family perspectives at M0 and M12: adapted from a tool developed by Tavistock Clinic and Portman NHS Trust (London).
**Other evaluations**	NA
**Statistical analysis**	Each score from each scale used and the data collected will all be described by the mean and standard deviation for continuous variables and by frequencies for qualitative data.<br>In order to measure the effectiveness of the integrative practices, the differences between the scores measured at baseline and at the M12 visit will be described by 95% confidence intervals obtained using a linear model with a random effect on the center (in order to adjust the results on the center). For the ACE-R scale with repeated measurements more than 2 times, the evolution will be additionally modeled by linear models with a random effect, allowing for the repeatability of the data.
**Submission to the group of Nantes of Ethics in Health Care (CNEDS)**	Planned submission to the GNEDS

LIST OF ABBREVIATIONS

AEFCP	Association for the Evaluation and Training of Psychiatrists
APIJB	Association of Infant and Child Psychiatry of Brittany
ARC	Clinical Research Associate
BLR-F	Brunet-Lezine Revised Early Childhood Psychomotor Development Scale
CARS	Childood Autism Rating Scale
CATTP	Center d'Action Thérapeutique à Temps Partel
CNIL	Commission Nationale de l'Informatique et des Libertés
CCTIRS	Advisory Committee on Information Processing in Health Research
CRF	Case Report Form (observation booklet)
CIM 11	International Classification of Diseases 11th edition
DSM 5	Diagnostic and Statistical Manual of Mental Disorders 5th edition
ECA-R	Autism Behavior Assessment Scale
ELO	Oral Language Evaluation
FFP-CNPP	French Federation of Psychiatry - National Council of Psychiatry
HAS	High Authority for Health
HJ	Day Hospital
PEP 3	Psycho Educational Profile Revised
TEC	Clinical Study Technician
TED	Pervasive Developmental Disorders
GNEDS	Nantais Group for Ethics in Health Care
RNI	Non-Interventional Research

TABLE OF CONTENTS

SUMMARY……………………………………………………………………………………………3
LIST OF ABBREVIATIONS………………………………………………………………….……6
TABLE OF CONTENTS……………………………………………………………………….…..7
INTRODUCTION…………………………………………………………………………………..8
1. RATIONALE FOR STUDY…………………………………………………………………….9
1.1. POSITIONING OF THE RESEARCH……………………………………………………..9
1.2. ORIGINALITY AND STRENGTHS……………………………………………………...11
1.3. BENEFITS………………………………………………………………………………...11
1.4. BIBLIOGRAPHY…………………………………………………………………………12
2. OBJECTIVES AND CRITERIA FOR JUDGING……………………………………...15
2.1. MAIN OBJECTIVE AND EVALUATION CRITERIA…………………………………..15
2.2. SECONDARY OBJECTIVES AND EVALUATION CRITERIA……………………….15
3. STUDIED POPULATION……………………………………………………………………..17
3.1. DESCRIPTION OF THE POPULATION …………………………………………...17
3.2. INCLUSION CRITERIA ………………………………………………………………….17
3.3. CRITERIA FOR NON-INCLUSION……………………………………………………...17
4. CONDUCT OF THE STUDY ………………………………………………………………..18
4.1. GENERAL RESEARCH METHODOLOGY……………………………………………..18
4.2. STUDY AND ANALYSIS TECHNIQUES ……………………………………………...18
4.3. TIMING OF THE STUDY…………………………………………………………………25
4.4 CRITERIA FOR EARLY TERMINATION OF AN INDIVIDUAL'S PARTICIPATION IN RESEARCH…………………………………………………………………………………….. 26
5. DATA MANAGEMENT AND STATISTICS…………………………………………… 27
5.1. COLLECTION AND PROCESSING OF DATA FROM THE STUDY…………………….. 27
5.2. STATISTICS……………………………………………………………………………… 28
6. SAFETY / UNDESIRABLE EFFECT…………………………………………………… 30
7. ADMINISTRATIVE AND REGULATORY ASPECTS……………………………………… 31
7.1. RIGHT OF ACCESS TO SOURCE DATA AND DOCUMENTS………………………….. 31
7.2. COMPUTERIZED DATA AND SUBMISSION TO THE CNIL…………………………….. 31
7.3. AMENDMENTS TO PROTOCOL…………………………………………………………. 31
7.4. RULES RELATING TO PUBLICATION ………………………………………………...31
8. ETHICAL CONSIDERATIONS…………………………………………………………… 32
8.1. PATIENT INFORMATION AND CONSENT……………………………………………….. 32
8.2. GROUPE NANTAIS D'ÉTHIQUE DANS LE DOMAINE DE LA SANTE (GNEDS)……. 32

INTRODUCTION

The research project is part of the evaluation of mental health therapies. It takes place in infant-juvenile units that have developed "integrative devices", by taking into account recent advances in knowledge and offering care, education and a pedagogical approach. The evolution of autistic children benefiting from these integrative devices is studied, in order to determine the validity of the cited interventions. The methodology is based on case studies in natural situations. The clinicians involved form a network, among themselves and with the researchers, within the framework of a multicentric study.

1. RATIONALE FOR THE STUDY

1.1. RESEARCH POSITIONING

Rationale for the study
Pervasive Developmental Disorders (PDDs) are a group of diverse clinical situations identified in eight categories in the ICD10 (International Classification of Diseases). Epidemiological studies in Fombonne in 2009 (1) found a prevalence of Pervasive Developmental Disorders (PDD) of 6 to 7 per thousand, and 2 per thousand for infantile autism, whereas according to the 2002 Inserm expertise (2). The prevalence of PDD is 27.3 per 10,000 and that of autism 9 per 10,000, these differences in prevalence are partly related to clinical criteria applied more widely.

Pervasive developmental disorders that are named in the new DSM V classification, and are likely to be named in the upcoming ICD 11, "Autism Spectrum Disorders", have the same diagnostic criteria. The increase in their prevalence could be related to the extension of the concept of spectrum, an evolution of diagnostic criteria, a better knowledge of the disorder by professionals and the development of early detection. Consequently, this pathology constitutes a real public health problem that mobilizes professionals, families and public authorities. It requires a dynamic of research and study both on the etiological level and on the understanding of the underlying mechanisms, and on the level of management under its dual therapeutic and educational aspects.

The management of autism in child psychiatry has greatly evolved in recent years, both in its theoretical references and in the care modalities that result from it. The need for children with pervasive developmental disorders to benefit from care that is as intensive and as early as possible is clearly recognized and consensual (3-4-5-6).

Beyond the positions that may have been dogmatic on this subject, the plurality of factors at play in the advent of autistic pathology leads to the implementation of multidimensional responses that do not prejudge the share of genetic and environmental elements (7-8-9). The psychodynamic theoretical corpus of child psychiatry and the experience of care, based on the analysis of the relationship, on the narration and analysis of anxieties, has made it possible to support institutional organizations and their therapeutic mediations (10-11); knowledge and methods of care have been enriched by the contributions of other works. Data on social cognition, empathy, theory of mind, imitation (12), and neurophysiological equipment (13) have thus been integrated into treatment methods. The specificities of autistic pathology, such as the particular modes of sensory functioning, of sharing representations and emotions for example, are taken into account in the approach to young patients. The description of the care units, in all their diversity, testifies to the evolution of their systems and the updating of knowledge.

Child and youth care units have developed coordinated multidisciplinary or integrative care systems.
This approach is defined by a set of coordinated interventions offered to a child in his or her globality and singularity, in relation to his or her parents and the usual living environment. It is based on complementary perspectives: psychopathological, physical and physiological and associates a plurality of interventions (therapeutic, educational, pedagogical) and a coordinated plurality of interveners. These systems have been questioned in recent years, in particular because they had not been sufficiently explained or evaluated. The recommendations of good practice of the HAS in 2012 (14) insist on the importance of

developing clinical research with the aim of knowing the long-term effects of the proposed interventions.
The 2013 autism plan reminds us of the importance of pursuing research on the development of evidence-based care, including the evaluation of therapeutic treatments. Action sheet 27 "Promoting clinical research on autism" defines the main lines of research, some of which emphasize the importance of early intervention.

The evaluation of mental health therapies is a highly complex problem (15-16-17) since they are not essentially based on drug treatments but combine educational, psychotherapeutic and pedagogical approaches (18-19).

It requires a methodology that takes into account, in a natural situation, the clinical diversity of cases and their specificities, the clinical processes involved, their effects and the variables underlying them (20-21-22-23). In the field of autism, it is practically impossible to define effective treatments in a global way because of the particular diversity of patients despite similar diagnostic criteria (24). In recent years, two studies carried out on the subject of therapeutics in the field of autism have contributed to the implementation of this research:
- the work of the Association de Psychiatrie Infanto-Juvenile de Bretagne (APIJB) in association with the Association pour l'Evaluation et la Formation des Psychiatres (AEFCP): evaluation of the care pathway of autistic children in infant-juvenile units - 2011
- the research network on psychotherapeutic practices (Unité Inserm Thurin, Falissard) (25-26): intensive case studies, associating to the longitudinal study of changes that of the mediators who underlie them and the moderators who partly condition them. They show that these methods can lead to very interesting results (27); some of the tools of the research network on psychotherapeutic practices and the reflection of its facilitators have contributed to the implementation of this research.

Care in the form of integrative management, as practiced in child psychiatry, has not been the subject of clinical research. Their coordinated, multidisciplinary and early character will be evaluated, for the first time, in their clinical effects from several child psychiatry services spread throughout France.

This research will also allow a better knowledge of integrative clinical practices, which will meet the expectations of professionals and users, of the various public health partners, of the supervisory authorities and of the HAS.

It will also allow the reinforcement of the **training** of the participating teams through exchanges between peers, and through the acquisition of knowledge contributing to interdisciplinary continuous professional development. Training in evaluation tools will be a prerequisite to the teams' involvement in the research. The teams will form a network to encourage exchanges and clinical observations and the homogenization of practices.

The Fédération Française de Psychiatrie-Conseil National de Psychiatrie (CNPP), with its colleges of child psychiatry and research, is a partner in this study.

1.2. ORIGINALITY AND STRENGTHS

To our knowledge, this is the first time that a multicenter research is proposed to evaluate integrative practices in child psychiatry for autism.
In the methodology, we have not introduced a control group since the main objective is the evaluation of care practices.

In this first research, the practices will be evaluated in their entirety in order to determine whether they lead to an improvement in the child's condition. Based on the results of this research, the question will then arise of comparing two practices with a control group in order to show the effectiveness of one practice in relation to the other, which seems too early with our current knowledge.
Integrative practices, implemented since the 1980s, have never been evaluated on a large scale. The objective of this project is therefore to evaluate these practices in their entirety to see if they lead to an improvement in the affected child over 1 year. This practice is globally that of public child psychiatry. Taking a control group of patients followed by these services by delivering, for example, a part (or none) of the treatment targeted on one area and with a single technique, whereas the children in the control group would be covered by a global treatment, raises an ethical question. Moreover, other studies justify that the profiles are so singular that having a control group does not make much sense. On the other hand, the main evaluation will be controlled by two neutral professionals.

Many services have committed to participate in this research, not all of them could be retained, and others would still like to do so, thus testifying to the willingness of public service professionals to evaluate their practices and their impact on the children's progress.

Parents' perception of their child's progress and the quality of the service provided are essential to the objectives of this study.
The parents' subjective assessment of this progress will be compared to that of the caregivers using the same criteria.

This research responds to the indications of the HAS recommendations (March 2012) and the 3rd autism plan (2013-2017).
The partnership with the FFP-CNPP opens a collaboration with the Direction Générale de la Santé (DGS) for the constitution of a network and its animation in order to facilitate its multicentric realization.

1.3. BENEFITS

1.3.1. Individual benefits

The children who participate in the research will receive therapy under careful and repeated evaluation.

1.3.2. Collectif benefits

This is a multicenter, prospective, open-label observational study. It is applied in the usual environment in care units offering an integrative system.

This research responds to the expectations expressed by civil society through the 2013 autism plan on the nature and efficiency of care in child psychiatry. By making them more readable, by insisting on the participation of parents, it will contribute to the improvement of trust and collaboration between professionals and users.

The professionals participating in the research will deepen their knowledge in the clinical field and in the evaluation of patients with typical or atypical autism through the shared use of evaluation scales.
The therapeutic modalities offered to autistic children will be better adjusted and therefore optimized.

1.4. BIBLIOGRAPHY

1. Fombonne E.- 2009 (18)- Epidemology of pervasive developmental disorders- Pediatric.Res 2009 ; 65 (6) 591-8
2. Expertise collective Inserm. Troubles mentaux : dépistage et prévention chez l’enfant et l’adolescent. Paris : Inserm, 2002
3. Bursztejn C. Est-il possible de dépister l'autisme au cours de la première année ? ENFANCE 2009 ; 61(1) : 55-66.
4. Delion P, Beucher A, Bullinger A, Carel A, Charlery M, Golse B, et al. Les bébés à risque autistique. Ramonville Saint-Agne : Erès ; 2002.
5. Fédération Française de Psychiatrie, Haute Autorité de Santé-Recommandations pour la pratique professionnelle du diagnostic de l’autisme- Paris, Saint Denis la Plaine- FFP ; HAS- 2005.
6. Fernell E, Hevall A, Westerlund J, Carlsson LH, Eriksson M, Olsson M.B, Holm A, Norrelgen F, Kjellmer L, Gillberg C, Early intervention in 208 Swedish preschoolers with autism spectrum disorder. A prospective naturalistic study, Research in Developmental Disabilities, 2011, 32, 2092-2101.
7. 72e journées nationales de la SFPEADA-Psychopathologie et Neurosciences-5et 6 juin 2009- Lille
8. Bargiacchi A, Zilbovicius M. Imagerie cérébrale et autisme. ANAE 2008 ; 20(100) : 291-7.
9. Golse B L’autisme entre neurosciences et pychanalyse-enfance et Psy n°46-2010- dossier : l’autisme aujourd’hui ed Erès
10. Schmit G, Bouvet M, Hincky M.-O. Secteur de psychiatrie infantojuvénile. EMC - Pédopsychiatrie 2008:1-20 [Article 37-211-A-05].
11. Hochmann J. Soin institutionnel aux enfants et aux adolescents souffrant de troubles graves et précoces du développement (autismes et psychoses de l'enfance). EMC - Pédopsychiatrie 2009:1-9 [Article 37-210-A-10].
12. Nadel J.Imitation et cognition sociale dans l'autisme.NEURO PSY NEWS 2007 ; 6(3) : 124-7.
13. Barthelemy C, Huc Chabrolles M, Tripi G, Gomot M, Martineau J, Bonnet Brilhault F. Les compléments neurophysiologiques du diagnostic. ENFANCE 2009 ; 61(1) : 89-92. AUTISME
14. HAS-ANESM, Autisme et autres troubles envahissants du développement : interventions éducatives et thérapeutiques coordonnées chez l’enfant et l’adolescent mars 2012.

15. Bréchot C, Danion JM, *et al*. Méthodologie de l'évaluation en psychiatrie et en santé mentale. Colloque Inserm 2007. *Pour la recherche* 2007 ; 54 (3) : 1-12.

16. Kovess V, *et al*. Évaluation de la qualité en psychiatrie. Paris, Economica 1994, 318 p.

17. Kovess V, Lopez A, Pénochet JC, Reynaud M. Psychiatrie des années 2000, Organisations, Évaluations, Accréditation. Médecine-Sciences Flammarion, 1999, 306p.

18. Thurin JM. L'évaluation des psychothérapies, où en sommes-nous ? in Fischman G L'évaluation des psychothérapies et de la psychanalyse. Paris Masson 2009, p 101-116.

19. Thurin JM. Évaluation des effets des psychothérapies. *EMC (Elsevier Masson SAS, Paris), Psychiatrie*, 37-802-A-10, 2009.

20. Lambert MJ. Bergin & Garfield's Handbook of psychotherapy and Behavior Change. Sixth Edition. NY, J Wiley & Sons, 2013

21. Kraemer HC, Stice E, Kazdin AE, Offord DR, Kupfer DJ. 2001. How do risk factors work together? Mediators, moderators, independent, overlapping, and proxy-risk factors. Am. J. Psychiatry 158:848–56.

22. Thurin JM, Briffault X. Distinction, limites et complémentarité des recherches d'efficacité potentielle et d'efficacité réelle : nouvelles perspectives pour la recherche en psychothérapie. *L'Encéphale*, 2006 ; 32 : 402-12.

23. APA Presidential Task Force on Evidence-Based Practice. (2006). Evidence-based practice in psychology. *American Psychologist*, 61, 271–285.

24. Mesibov GB & Shea V. Evidence-Based practices and autism. *Autism* 2011 15 : 114-133.

25. Thurin JM, Falissard B, Danion JM. Réseau de recherches fondées sur les pratiques psychothérapiques. Pour la recherche 2008 ; 56

26. Thurin JM, Thurin M & Midgley N: Does participation in research lead to changes in attitudes among clinicians? Report on a survey of those involved in a French practice research network, *Counselling and Psychotherapy Research: Linking research with practice* 2012 ; 12:3, 187-193

27. Haag, Amenta, Messica, Thurin M et JM, Vassallo P, Lanteri A, Pasero L. Résultats préliminaires de 20 études intensives de cas du Pôle italien du RRFPP. *Pour la recherche* 2012 ; 73 :1-16.

2. OBJECTIVES AND CRITERIA OF JUDGMENT

2.1. OBJECTIVE AND PRIMARY CRITERIA OF EVALUATION

2.1.1. Principal objective

Evaluation of care practices, based on the evolution over one year of children aged 3 to 6 years, presenting a diagnosis of typical or atypical autism (F 84-0 and F 84-1), cared for in a care unit practicing integrative approaches (Centre Accueil Thérapeutique à Temps Part-Time CATTP, Day Hospital).

2.1.2. Principal evaluation criteria

Developmental assessment in the domain of verbal and preverbal cognition and maladaptive behaviors in the domain of affective expressions of children between time M0 (month of inclusion) and M12 (12th month of follow-up) according to the PEP 3 scale.

2.2. SECONDARY OBJECTIVES AND EVALUATION CRITERIA

2.2.1. Secondary objective(s)

- Demonstrate the effectiveness of integrative practices by evaluating the overall progress of children according to clinical criteria and validated and selected evaluation tools.

- Evaluation by the families :
- the child's condition and its evolution
- the quality of the relationship established by the team with the family.

- To contribute to the homogenization of care practices.

2.2.2. Secondary evaluation criteria

- Global evolution of the child assessed by developmental domain (language and communication, sensory-motor, social interactions, behaviors, cognitive domain, anxieties and emotions) with the help of scales, ECA-R, CARS and speech therapy (ELO) and psychomotor (Brunet-Lézine) assessments at M0 and M12

Domain evaluated	**Tools**
Language et Communication	ELO de Khomsi speech therapy assessment
Sensori-motor	Brunet-Lezine Psychomotor assessment
Social interactions	ECA-R 29 items
Autistic behaviors	ECA-R
Cognitive, global	PEP3 131 developmental items and 43 behavioral
Anxiety, emotions and global evaluation	CARS 15 items (or subscales)

The structured clinical observation or case study includes the child's anamnesis, the history of his or her care, elements of the child's and family's life context, the different areas of his or her development appreciated in the relationship with the caregiver and the proposed care project. This element could be part of a qualitative study, the analysis of which could be carried out by a psychologist from the human sciences.

- Families' point of view assessed by a questionnaire at M0 and M12: adaptation of a tool developed by Tavistock Clinic and Portman NHS Trust (London).
It consists of a pre-treatment questionnaire with 30 questions, a questionnaire at the end of the study period with 44 questions, an assessment of the child's condition "how would you describe your child's development? " with 10 items rated from 0 to 4.

3. STUDIED POPULATION

3.1. POPULATION DESCRIPTION

Eighty children, aged 3 to 6 years, will be recruited for this research, within a year of entering a day hospital or part-time therapeutic center.
Prior to this admission, the diagnostic process (including the somatic assessment) and the establishment of the therapeutic relationship will be carried out, which will require a series of procedures over a period of 3 to 6 months.
In these children, the diagnosis of typical or atypical autism is made prior to entry into research.
The relatively limited number of children (80) entering this research corresponds to the requirement of delimiting a relatively homogeneous population defined by the categories chosen from the broader autistic spectrum.
Recruitment of the population will be done by health care teams selected because they use integrative methods and have been trained in the tools of the research.

3.2. INCLUSION CRITERIA

General Inclusion Criteria :
- Children 3 to 6 years of age with a diagnosis of F84.0 and F84.1 according to ICD-10 criteria. The diagnosis must have been made according to the criteria established by the Diagnostic Guidelines (FFP/HAS 2005).
- Children cared for in care units meeting defined criteria for integrative practices.
- Children receiving a volume of intervention hours between two and four half-days per week.
- Written parental consent.

3.3. NON-INCLUSION CRITERIA

Presence of co-morbidity such as epilepsy, severe organic, somatic and sensory impairment.

4. STUDY PROCESS

4.1. GENERAL RESEARCH METHODOLOGY

It is a prospective multicentric study, in natural situation, involving teams from the sectors of child and youth psychiatry of the national territory that share the approach defined in the argumentation of the study.
The research has the following characteristics :

- Study on therapeutic approaches,
- National multi-center study,
- Uncontrolled study,
- Non-randomized natural situation study,
- Open study,
- Prospective study.

Our methodology is based on the evaluation of the child in the different areas of his or her development according to validated and recommended tools. Assessments take place at the beginning and end of the study and at intermediate times. They notably allow us to evaluate the adjustment of integrative practices to the particularities and needs of the child, the evolution of the child being an element of the evaluation of the effectiveness of the practices.
The families' point of view, provided by a questionnaire at the beginning and end of the study, contributes to the data collection. The same questionnaire will be submitted to the child's referring professionals, thus allowing a comparison of the subjective representations of the child that each one forms during the care process.
Children will be included after a diagnosis validated according to the recommendations of the FFP and the HAS of 2005 and after informing and obtaining the written consent of the parents.
The first evaluation of the child by domains will be carried out at M0 using validated scales. The profile of the child thus defined will be the basis for the elaboration of the care project. It also includes a questionnaire to families, professionals and an inter-judicial evaluation. The same evaluation will be carried out at M12.
Intermediate assessments at M3, M6, M9 will be based on a behavioral scale.
The doctors in charge of the investigating centers will participate in meetings with the aim of appropriating the methodology, acquiring a sufficient level of training, transmitting them and submitting to the group thus formed the questions that arise as the project progresses. A meeting every four months would be necessary with the participation of the FFP-CNPP.

4.2. STUDY AND ANALYSIS TECHNIQUES

4.2.1. Detailed description of evaluation parameters

Evaluation tools

1- Revised Psycho-Educational Profile (PEP 3) by Schopler (1994)

The PEP is a tool that allows the cognitive and behavioral profile of the autistic child to be defined in order to adapt educational proposals as closely as possible to the child's particularities.
It evaluates current and emerging abilities in seven areas: imitation, perception, gross motor skills, oculomanual coordination, cognitive performance, and verbal cognition. It evaluates behaviour in the following areas: relationships and affect, play and interest in materials, sensory responses and language.
It is suitable for children under 7 years of age.
It allows to visualize strengths and weaknesses and emerging ones. It includes 113 developmental and 43 behavioral items. The different tests are based on play and teaching materials and are presented to the child during structured play sequences. The examiner observes, assesses and records the child's reactions. The scores obtained are distributed in seven developmental and four behavioural areas. PEP 3 has 3 types of ratings: success, failure and emergence. For behavior, the ratings are appropriate, mild and severe.
It is on the basis of this instrument that the child's development between M0 and M12 will be evaluated. The psychologist of the study will carry out this evaluation as well as two other independent psychologists. The results that will be taken into account at M0 and M12 will be those of the independent psychologists.

2- ACR-R: Assessment of Autistic Behaviour (revised version) by G. Lelord and C. Barthélémy

This scale has been validated in the child psychiatry department of Tours. It includes 29 items rated from 0 to 4 according to their frequency of appearance. It allows the autistic child to explore different areas of his/her behaviour: social withdrawal, verbal and non-verbal communication disorders, adaptation to environmental situations, tonicity disorders, disturbed motor skills, emotional reactions, disorders of major instinctive functions, disorders of attention, perceptions and intellectual functions. It indicates, for the different behaviours observed, the spontaneous variables over time and the improvements induced by therapy and rehabilitation.
From the regular scoring of the scale, symptomatic profiles are obtained for a child. This profile can be followed over several months. It is possible to analyze the evolution of this profile, symptom by symptom, taking into account the different factors that may have influenced this evolution.
The quantified data collected from the scale can also be used as clinical variables to investigate possible relationships with other variables. It is thus possible to follow the evolution of "relational impairment" and "modulating insufficiency" on the basis of specific summary scores.

3- Childhood Autism Rating Scale Childood Autism Rating Scale by Schoopler et al (1980) (CARS)

It is a scale of evaluation and intensity. It is composed of 14 sub-scales or areas of evaluation: social relationships, imitation, emotional responses, adaptation to changes, use of the body, objects, visual and auditory responses, taste, smell, touch, fears and anxiety, verbal and non-verbal communication, activity level, intellectual level. A 15th scale measures the examiner's overall impression of the severity of autism. Each subscale is described in the manual along with the main elements to be retained. Each item is rated from 1 to 4, with 4 being severely abnormal. The total score ranges from 15 to 60, with a cut-off score of 30. From 30 to 36.5 we are in the moderate autism register, from 37 we are in the severe autism register.
This scale has been the subject of numerous validation studies, and can be used from 24 months onwards, during play and daily living sessions. It can be filled in by more than one observer, which makes it possible to check the inter-judge agreement.
It is an interesting and formative tool for clinical observation in health care services.

4- Speech and language assessment

The speech-language pathology assessment should bring together all the information concerning the way the child communicates in order to establish a profile of his or her communication means and functions. It is a question of highlighting the difficulties but also the emergences and skills of the child.
This speech and language assessment is complex, in particular because:
- the few specific tools available, which obliges each speech therapist to evaluate the child's skills and deficits mainly through personal observation.
- the very nature of the pathology (attentional, behavioural and language disorders) which makes it difficult to assess comprehension.

It is important to assess the prerequisites for the communication function by :
- The gaze
- Gestural and verbal imitation, the game of make-believe
- Joint attention
- Facial expressions, mimics and praxies.
- Symbolic gestures, proto-imperative and proto-declarative pointing and social gestures.
- The vocalizations

Oral Language Evaluation - ELO

This test was developed in the 80s and regularly revised by A. Khomsi, professor of psychology and linguistics.
This battery of six tests is designed to describe and evaluate, in a fine detail, various components of oral proficiency.
It is composed of the following tests:
Vocabulary, Phonology, Comprehension and Linguistic Production, both in reception and production.
A detailed clinical analysis of these areas allows us to establish individual profiles and to identify the component that needs to be addressed, either directly or indirectly.
- Vocabulary includes two tests: Lexicon in Reception (LexR) (20 plates). Lexicon in Production (LexP) (series of 50 images) (series of 10 images for the youngest).
- Comprehension includes two sub-tests according to the age of the child.
- Phonology with a Word Repetition test (RepM).
- Linguistic production. Two tests are used to explore the child's morphosyntactic skills: Word Repetition for kindergarten children. Statement production in which the child completes a sentence. This test highlights linguistic dysfunctions involving the

processing of morphology or syntax, as well as pragmatic dysfunctions in which the child does not take into account the proposed linguistic and pragmatic context.

Profiles constructed from the overall scores allow the identification of the particular characteristics of each child's functioning. They are supplemented by profiles specific to each test, constructed from various calculated sub-scores that allow for a more refined analysis of the cases. Most of the tests are organized in such a way that a stopping criterion makes it possible not to use all the items. In this case, the items not used can be used during or at the end of the treatment.

For each child, his or her starting profile will be retained and re-evaluated at time M12.

5- Le Brunet - Lézine: evaluation of the child's psychomotor development

The Brunet-Lezine is a test developed in the 1950s and revised several times, most recently in 2001. It is commonly used in child and youth psychiatry services by psychomotor therapists.
It consists of a set of tests ordered by age and a series of questions for parents. These tests are used to calculate a developmental quotient.
This tool is adapted to children from 0 to 5 years old. The analysis of the results allows the calculation of Developmental Quotients (DQ) ages. Four are partial and concern the four domains evaluated (named below), the last one is global.
The four areas of development are :
- Postural control and motor skills (P-rated): tests of locomotion and postural control of the child in the dorsal, ventral, sitting and standing positions;
- oculomotor coordination and adaptive behaviour in relation to objects (graded C): study of grasping, the child's behaviour in relation to objects, and imitation of gestures;
- language (noted L): study of language in its functions of comprehension and expression;
- social relations (graded S): study of social relations including adaptation to different social and daily situations, self-awareness and awareness of others.

For each age, there are 10 tests. However, the number of tests in each of the 4 areas of development varies from one age to another, reflecting the typical behaviours of each age. Assessment is in terms of passing or failing the test.
This test lasts approximately 40 minutes. It consists of 17 age levels between 1 and 30 months. The score obtained is used to establish a Development Quotient (DQ).
Although this test is not specific to autistic pathology, it takes into account criteria that are sufficiently sensitive for this type of pathology. It also gives a relatively relevant vision of the level of the child's developmental capacities.
6- Questionnaires Famille – Questionnaires Professionnels (Annexe 3, Tableau 1, 2, 3 et 4)

We felt it was important to collect the families' views through a questionnaire at the beginning and end of the study. To this end, we adapted a tool developed by the Tavistock Clinic and Portman NHS Trust (London).
This center for child and youth psychiatry and psychotherapy in the United Kingdom (London) has a unit specialising in the care of autistic children. The professionals in this unit developed two questionnaires to take into account parents' observations about :
- the perception of the child's symptoms,
- the evolution of the demonstrations,
- appreciation of the quality of the relationship between professionals and them.

It includes a questionnaire with 30 questions prior to taking charge.
The questionnaire at the end of the study period consists of 44 questions.

It includes a pre-home questionnaire with 30 questions, concerning the parents' main concerns about the main manifestations of the child who is the source of the request for care. It details the parents' point of view on the child's development mainly in the area of social and imaginary communication.
A questionnaire at the end of the study period with 44 questions records the intensity, duration and quality of treatment and the links between partners. It includes the child's evolution in the different areas as well as a general assessment of the child's condition based on 10 items rated from 0 to 4.
This questionnaire will be submitted to the parents at times M0 and M12.
The same type of questionnaire will also be submitted to professionals at the M0 and M12 levels in order to compare the child's points of view.

7- Inventory of external factors (Appendix 3, Table 5)

Significant events occurring during the year of the research, concerning the child or his or her family, are noted. The impact on the child's behaviour and development will be analyzed.

Within the framework of the secondary objectives of this research it is planned to contribute to the homogenization of care practices.

8- Contribution to the training of professionals, to the evaluation and harmonization of their practices (Appendix 3, Table 6)

This research contributes to the training of professionals operating in child and youth psychiatry services through a more refined analysis of the child's profile carried out during the various stages through clinical observation, the appropriation of evaluation tools and collaboration with parents.

An evaluation grid based on knowledge of the scales used is submitted to professionals at the beginning and end, as well as an assessment of their interest in therapeutic management.
This reinforcement of training contributes to the homogenization of professional practices in the field of autism, which is one of the secondary objectives of the study.

4.2.2. Description des techniques et analyses

1- Integrative approach

An integrative approach is defined by a set of coordinated interventions proposed to a child apprehended in its singularity and its globality, in relation with the parents. It promotes the maintenance of the child in his or her usual living environment, including this environment as a support for the therapeutic and educational project.

This approach is based on the linking of complementary perspectives: psychopathological (psycho-dynamic, developmental and cognitive), physical and physiological (with all aspects of the child's somatic equipment: sensory, neurological, biological, genetic...) leading to the development of a shared understanding between professionals and parents and to an individualized care project.

It associates :
- The plurality of interventions: therapeutic (constituted by workshops or therapeutic groups carried out by nurses and/or educators, psychotherapies...), re-educational,

educational, pedagogical (within the school and/or the teaching unit of the institutional place).
- The plurality of the interveners

Interventions take into account different domains or dimensions: sensorimotor - communication and language - emotion, anxiety and behaviors - cognitive/pedagogy - socialization (distal autonomy) - educational (proximal autonomy) - family resources - somatic and pharmacological follow-up...
The coordination of interventions and interveners is carried out both with a psychiatrist referent and another referent (educator, nurse) who are committed to the child and his family and who guarantee the continuity of the project (this conception of coordination differs from a coordination carried out by the MDPH, or by the parents of the child themselves, or on the basis of an unorganized exchange between various teams or professionals). At the intra-institutional level, it is based on clinical syntheses, real spaces for connection and elaboration of the clinical material resulting from the different actions and therapeutic mediations, and on extra-institutional articulations (meeting with the school institution...).
The interventions and the individualized care project are based on structured clinical observation.

1- Structured clinical observation report format (Appendix 3, Table 7)

In clinical practice in child and youth psychiatry, according to the integrative approach, all professionals pool their observations to arrive at a clinical synthesis. It reports on the overall evolution of the child, articulating a subjective and an objective perspective.

These interventions are organized into an individualized project that integrates the child's particularities and needs.

2- The Individualized Care Project (Appendix 3, Table 8)

The individualized care project is built from the elements collected during structured clinical observation, assessments in the different areas, observation of parents and other caregivers.
It defines the objectives of care and the means to achieve them. The term "individualized care project" emphasizes the dynamic and revisable aspects and its personalized adaptation to the child's profile and specific needs. The project integrates the three components: care, education and pedagogy, in accordance with the public health code and the recommendations of the HAS.
The project includes the interventions that the child receives with reference to the activity chart that explains the therapeutic, educational and pedagogical objectives of the different interventions proposed.
The place of each professional is defined, as well as their coordination, the modalities of articulation with the families and the other interveners. The regular review of the project is noted.

3- Description of therapeutic and educational interventions (Appendix 3, Table 9)

The Individualized Care Project includes both individual and small group interventions.
The therapeutic workshops and activities offered use a wide variety of materials. Generally speaking, they aim to develop new skills and experiences and they contribute

to the establishment of the relationship through the support of the caregivers. The common denominator is the attention paid to the child's manifestations, taking them into account, decoding them in order to help the child acquire tools to better perceive himself and the surrounding world.

Within the units, we can distinguish :
- Daily activities (welcome and departure, meals or snacks, dressing, transition times, outings, personal hygiene and sphincter education, etc). These activities contribute to the temporal and psychosocial identification of the child, to the reduction of anxiety associated with his or her relationship to the world and to others, and to his or her education. They provide clinical material on the child's functioning. They give rise to observations to be shared with families in order to find common strategies.
It should also be kept in mind that incidental learning takes place and that a single activity allows several dimensions to be worked on.

- Therapeutic workshops or groups or therapeutic mediations.
They allow to take into account the language of the body, of the affect, of the sensor-motor; they aim at the acquisition of representation capacities and social and cognitive skills. The workshops take place according to a precise framework: starting from an indication based on defined objectives; they take place in a regular time and space, for a determined and renewable duration; they are led by professionals who are referents of this workshop.

- Individual interventions.
These can be interventions by the nurse or the educator (on the child's care or living places), psychomotricity, speech therapy, psychotherapy and pedagogical intervention. Some interventions are carried out in the presence of one or more parents, with one or more professionals.

- The family's interviews with the child psychiatrist and/or the psychologist and/or another professional from the team represent key moments allowing the adjustment of the project, the sharing of representations and different levels of support.

- Articulations
A work of link is constantly sought between the professionals concerned by the child in the form of meetings, clinical meetings, etc.

For the needs of the study, and in a voluntarily systematized way, the mediations will be described by correlating them to the functional area(s) they allow to address with the child (see table, appendix).

4.3. STUDY CALENDAR

Actions	M0 (Inclusion visit)	M2	M3	M6	M9	M12
Diagnostic stage = Validated diagnosis	X					
Parents' information	X					

First evaluation (CARS, PEP-3, ECA-R, speech therapy, psychomotor, external factors inventory, family and professional questionnaires, inter-judge evaluation, Cr of structured clinical observation)	X					X
Individualized Care Project Chart	X					
Behavioral Change Indicators (BCA-R)			X	X	X	
Last evaluation = Study exit						X

4.4. CRITERIA FOR PREMATURE TERMINATION OF AN INDIVIDUAL'S PARTICIPATION IN RESEARCH

The criteria for premature termination of research are: children leaving the institution where the research is taking place, parents deciding to discontinue the child's inclusion in the research.

For the modalities and duration of follow-up for those who prematurely stopped the study, see the statistics section.

5. DATA MANAGEMENT AND STATISTICS

5.1. COLLECTION AND PROCESSING OF STUDY DATA

5.1.1. Data collection

An observation notebook will be created per patient. All information required by the protocol must be provided in the CRF. It must include the data needed to confirm compliance with the protocol and all data needed for statistical analyses and to identify major deviations from the protocol.
The person responsible for completing the CRFs in each center will be the child psychiatrist, the principal investigator.
Once completed, the pages of the paper CRFs will be mailed out as they are completed, and will be centralized in Nantes for data entry into the electronic "Clinsight" database by the Clinical Trial Technician (CTT).

Filling rules will be defined.
The data will have to be copied in a clear and readable way.
Missing data will be notified.
Incorrect data will be clearly crossed out and new data copied next to it with the date and initials of the corrector.

5.1.2. Codage des données

By signing this protocol, the principal investigator and all co-investigators agree to keep confidential the identities of the children (and parents) who participated in the study.

The transmission of an individual's data for research purposes will therefore only be possible subject to the application of a coding system; the presentation of research results must exclude any direct or indirect identification.

The patient code will consist of the child's initials (first letter of first name and first letter of surname), month and year of birth and the number of the center (from 1 to 17). This code will be the only information that will appear on the observation notebook (CRF) and will allow the CRF to be linked to the patient afterwards.
The person in charge of the research is also required to code the patient data on all documents in his/her possession (reports of imaging and biological examinations, etc.) that would be attached to the CRF.

5.1.3. Data processing

The collection of clinical data will be based on the implementation of a clinical database and the creation of data entry masks similar to the observation notebook in accordance with the protocol and regulations currently in force.
The structure of the database and the input screens will be approved by the person in charge of the research.
Data entry will be carried out in the Clinsight database, a software developed by the Data Manager of the Promotion Department of the Nantes University Hospital. This e-CRF will be accessible via a login and password from the website https://www.hugo-online.org/csonline.

5.2. STATISTICS

Name and contact details of the person in charge of the analysis: Jean-Benoit HARDOUIN, EA 4275 "Biostatistics, Pharmacoepidemiology and Subjective Health Measures", University of Nantes.

5.2.1. Description of the planned statistical methods, including the schedule of planned interim analyses

Each score from each scale used and the data collected will all be described by mean and standard deviation for continuous variables and by frequencies for qualitative data.

In order to measure the effectiveness of integrative practices, the differences between the scores measured at baseline and at the M12 visit will be described by 95% confidence intervals obtained using a linear model with a random effect on the center (to adjust for center outcomes). For the ECA-R scale resulting in measurements repeated more than 2 times, the evolution will also be modelled by linear models with a random effect, in order to take into account the repeatability of the data.

5.2.2. Statistical justification of the number of inclusions

Given the descriptive and innovative nature of the research, it was not possible to determine the number of subjects needed. The choice to include 80 children was therefore made based on the feasibility of the study, including as many children as possible.

5.2.3. Expected level of statistical significance

The expected degree of statistical significance is 5%.

5.2.4. Statistical criteria for stopping the search

Non applicable.

5.2.5. Method of accounting for missing, unused or invalid data

Missing data will not be imputed. Nevertheless, in case of numerous missing data (more than 10%), sensitivity analyses will be performed in order to apprehend the impact of the missing data on the results, by imputing the missing data by different scenarios (average of other patients, worst case, best case...).

5.2.6. Management of changes to the initial strategy analysis plan

The analysis strategy will not be modified, however, it will be possible to carry out additional analyses not initially planned.

5.2.7. Selection of persons to be included in the analyses

All children included in the study will be analyzed.

6. SAFETY / UNDESIRABLE EFFECT

The occurrence of an Adverse Effect related to the management of the patient during this protocol will be reported to the appropriate vigilance system (pharmacovigilance, biovigilance, hemovigilance, material vigilance, etc.).

7. ADMINISTRATIVE AND REGULATORY ASPECTS

7.1. ADMINISTRATIVE AND REGULATORY ASPECTS

The medical data of each patient will only be transmitted to the body of the person responsible for the research or any person duly authorized by that body under conditions guaranteeing their confidentiality.
Where appropriate, the body of the person responsible may request direct access to the medical file for verification of procedures and/or research data, without breaching confidentiality and within the limits permitted by laws and regulations.

7.2. COMPUTERIZED DATA AND SUBMISSION TO THE NCCL

The data collected during the study will be kept in a computer file in compliance with the French law "informatique et libertés" of January 6, 1978 amended in 2004.

The protocol will be submitted to the CCTIRS, and the computerized processing will be subject to a request for authorization from the CNIL.

7.3. AMENDMENTS TO THE PROTOCOL

The modified protocol will have to be the subject of a dated updated version.
The briefing note should be amended as necessary.

7.4. RULES RELATING TO PUBLICATION

A copy of the publication will be given to the CHU of Nantes, responsible for the research of the study, which will necessarily be cited. The authors will be determined in proportion to the number of patients included. The coordinating investigator will establish the list of authors.

Any publication will mention the financial support by the DGOS with the following mention: "This study is funded by the "Département général de l'offre de soins" (DGOS, French Ministry of Health)".

8. <u>ETHICAL CONSIDERATIONS</u>

8.1. PATIENT INFORMATION AND CONSENT

The investigator undertakes to inform the parents of the protocol in a clear and fair manner (information note in appendix 2). As the child's pathology and age do not allow a clear understanding of the protocol, only the parents will be informed of the study. In the best case, a very simplified oral information can be given to the child. The investigator will give the parents a copy of the information note with the consent request. This will specify the possibility for parents to refuse their child's participation in the research.

The written consent of at least one of the parents will be obtained before their child is included in the study.

The investigator will note in the patient's file that the patient's parents have been informed orally. The investigator will include a copy of the signed consent for their child to participate in the research in the chart and give the other copy to the parents.

8.2. ETHICAL GROUP FROM NANTES IN HEALTH CARE

The protocol and the information note and consent will be submitted for the opinion of the Nantes Health Ethics Group.
