# Supplementary material for: Migration is associated with baseline severity and progress over time in autism spectrum disorder: Evidence from a French prospective longitudinal study
Source: PLoS One. 2022 Oct 6;17(10):e0272693. doi: 10.1371/journal.pone.0272693 (PMC9536617; doi:10.1371/journal.pone.0272693)
Supplement: S2 File — (PDF) [file pone.0272693.s002.pdf]

Protocole AUTISME

«*Evaluation clinique des pratiques intégratives en unités de soins infanto-juvéniles pour des enfants présentant un autisme typique ou atypique* »

Investigateur Coordonnateur ou personne qui dirige et surveille la réalisation de la recherche :

Dr Nicole Garret-Gloanec
Praticien Hospitalier, Chef de Service,
Service de Pédo-Psychiatrie II, Hôpital Saint Jacques – CHU de Nantes
Rue de Saint-Jacques, 44093 Nantes cedex 1
Tel : 02 28 08 84 90
Mail : nicole.garret@wanadoo.fr

Méthodologiste :

Jean-Benoit HARDOUIN
EA 4275 « Biostatistique, Pharmacoépidémiologie et Mesures Subjectives en Santé »
Université de Nantes, Faculté de Médecine, bureau 221
1, rue Gaston Veil – BP 53508, 44035 Nantes cedex 1
Tel : 02 40 41 28 29
Fax : 02 40 41 29 96
Mail : jean-benoit.hardouin@univ-nantes.fr

Etablissement responsable de la recherche :

CHU de Nantes
Contact : Anne Omnès
Département promotion, Direction de la Recherche
5, allée de l'île Gloriette
44 093 Nantes cedex 01 (FRANCE)
Contact : Tel : 02 53 48 28 35
Fax : 02 53 48 28 36

Partenaire (s) :

Jean-Michel THURIN, Psychiatre, Inserm U669

Tel : 02 53 48 28 35
Fax : 02 53 48 28 36

Fédération Française de Psychiatrie
Hôpital Sainte Anne – Paris
Tel : **01 48 04 73 41 –** Fax : **01 48 04 73 15**

RESUME

**Titre de l'étude**	Evaluation clinique des pratiques intégratives en unités de soins infanto-juvéniles pour des enfants présentant un autisme typique ou atypique
**Mots clés**	Pratiques intégratives, évaluation clinique, étude de cas, autisme, psychiatrie de l'enfant, unités de soins
**Responsable de la recherche**	**CHU DE NANTES**
**Investigateur coordonnateur**	Docteur Nicole Garret-Gloanec
**Responsable scientifique**	Professeur Olivier Bonnot
**Investigateurs co -coordonnateurs**	Docteurs Fabienne Roos Weil et Maria Squillante
**Nombre de centres prévus**	Etude nationale : 17 centres prévus
**Type d'étude**	Recherche non Interventionnelle
**Planning de l'étude**	❖ Durée totale : 40 mois<br>❖ Période de recrutement : 36 mois<br>❖ Durée de suivi par patient : 12 mois
**Design de l'étude**	❖ Multicentrique<br>❖ Observationnelle<br>❖ En situation naturelle<br>❖ Non Contrôlée<br>❖ Ouverte<br>❖ Prospective
**Objectifs de l'étude**	Objectif principal : **L'évaluation des pratiques de soin**, à partir de l'évolution sur un an, d'enfants âgés de 3 à 6 ans, présentant un diagnostic d'autisme typique ou atypique (F 84-0 et F 84-1), soignés dans une unité de soins pratiquant les approches intégratives (Centre Accueil Thérapeutique à Temps Partiel CATTP, Hôpital de Jour).<br><br>Objectifs secondaires :<br>- Montrer l'efficacité des pratiques intégratives par l'évaluation de l'évolution globale des enfants appréciée selon des critères cliniques et des outils d'évaluation validés et sélectionnés.<br><br>- Evaluation par les familles :<br>- de l'état de l'enfant et son évolution<br>- de la qualité de la relation établie par l'équipe avec la famille.<br><br>- Homogénéiser l'utilisation des échelles d'évaluation.

**Nombre de cas prévisionnel**	80 patients
**Calendrier des différentes visites et des différents examens**	- Visite M0 : recueil du consentement des parents, inclusion, évaluation des dimensions du développement par des outils standardisés, observation clinique structurée, questionnaire famille n°1, questionnaire professionnel n°1.<br>- Supervision par un assistant de recherche des évaluations faites à M0 et M12.<br>- Visites M3, M6, M9 : échelle d'évaluation du comportement autistique et évènements éventuels du contexte.<br>- Visite M12 : visite de fin d'étude, évaluation des dimensions du développement par des outils standardisés, observation clinique structurée, questionnaire famille n°2, questionnaire professionnel n°2.
**Critères principaux de sélection, d'inclusion, de non-inclusion et d'exclusion**	**Critères d'inclusion généraux** :<br>- Enfants de 3 à 6 ans avec diagnostic F84.0 et F84.1 selon les critères de la CIM-10. Le diagnostic devra avoir été fait selon les critères établis par les recommandations sur le diagnostic (FFP/HAS 2005).<br>- Dans des unités de soins répondant aux critères définis de pratiques intégratives.<br>- Enfants recevant un volume d'heures d'intervention entre deux et quatre demi-journées par semaine.<br>- Recueil du consentement des parents.<br><br>**Critères de non inclusion :** présence de co-morbidité telle qu'épilepsie, atteinte organique grave, somatique et sensorielle.
**Critère de jugement principal**	Evaluation développementale dans le domaine de la cognition verbale et préverbale et des comportements inadaptés dans le domaine des expressions affectives des enfants entre le temps M0 (mois d'inclusion) et M12 (12e mois de suivi) selon l'échelle PEP 3.
**Critère(s) de jugement secondaire(s)**	- Evolution globale de l'enfant appréciée par domaine de développement (langage et communication, sensori-moteur, interactions sociales, comportements, domaine cognitif, angoisses et émotions) à l'aide des échelles ECA-R, CARS et des bilans orthophonique (ELO) et psychomoteur (Brunet-Lézine) à M0 et M12.<br><br>- Point de vue des familles apprécié par questionnaire à M0 et à M12 : adaptation d'un outil mis au point point par Tavistock Clinic and Portman NHS Trust (London).
**Autres évaluations**	NA
**Analyse statistiques**	Chaque score issu de chaque échelle utilisée et les données recueillies seront toutes décrites par la moyenne et l'écart-type pour les variables continues et par les fréquences pour les données qualitatives.<br>Afin de mesurer l'efficacité des pratiques intégratives, les écarts entre les scores mesurés à la baseline et à la visite à M12 seront décrits par des intervalles de confiances à 95% obtenu à l'aide d'un modèle linéaire avec un effet aléatoire sur le centre (afin d'ajuster les résultats sur le centre). Pour l'échelle ECA-R donnant lieu à des mesures répétées plus de 2 fois, l'évolution sera en outre modélisée

	par des modèles linéaires à effet aléatoire, permettant de tenir compte de la répétabilité des données.
**Soumission au Groupe Nantais d'Ethique dans le Domaine de la Santé (GNEDS)**	Soumission au GNEDS prévue

LISTE DES ABREVIATIONS

AEFCP	Association pour l'Evaluation et la Formation des Psychiatres
APIJB	Association de Psychiatrie Infanto-Juvénile de Bretagne
ARC	Attaché de Recherche Clinique
BLR-F	Brunet-Lezine Révisé Echelle de développement psychomoteur de la première enfance
CARS	Childood Autism Rating Scale
CATTP	Centre d'Action Thérapeutique à Temps Partiel
CNIL	Commission Nationale de l'Informatique et des Libertés
CCTIRS	Comité Consultatif sur le Traitement de l'Information en Matière de Recherche dans le Domaine de la Santé
CRF	Case Report Form (cahier d'observation)
CIM 11	Classification Internationale des Maladies 11ème édition
DSM 5	Diagnostic and Statistical Manual of Mental Disorders 5ème édition
ECA-R	Echelle d'Evaluation des Comportements Autistiques
ELO	Evaluation du Langage Oral
FFP-CNPP	Fédération Française de Psychiatrie – Conseil National de Psychiatrie
HAS	Haute Autorité de Santé
HJ	Hôpital de Jour
PEP 3	Psycho Educational Profile Révisé
TEC	Technicien d'Etude Clinique
TED	Troubles Envahissants du Développement
GNEDS	Groupe Nantais d'Ethique dans le Domaine de la Santé
RNI	Recherche Non Interventionnelle

TABLE DES MATIÈRES

RESUME 3
LISTE DES ABREVIATIONS 6
TABLE DES MATIERES 7
INTRODUCTION 8
1. JUSTIFICATION DE L'ETUDE 9
1.1. POSITIONNEMENT DE LA RECHERCHE 9
1.2. ORIGINALITE ET POINTS FORTS 11
1.3. BENEFICES 11
1.4. BIBLIOGRAPHIE 12
2. OBJECTIFS ET CRITERES DE JUGEMENT 15
2.1. OBJECTIF ET CRITERE D'EVALUATION PRINCIPAL 15
2.2. OBJECTIFS ET CRITERES D'EVALUATION SECONDAIRES 15
3. POPULATION ETUDIEE 17
3.1. DESCRIPTION DE LA POPULATION 17
3.2. CRITERES D'INCLUSION 17
3.3. CRITERES DE NON INCLUSION 17
4. DEROULEMENT DE L'ETUDE 18
4.1. METHODOLOGIE GENERALE DE LA RECHERCHE 18
4.2. TECHNIQUES D'ETUDES ET D'ANALYSES 18
4.3. CALENDRIER DE L'ETUDE 25
4.4. CRITERES D'ARRET PREMATURE DE LA PARTICIPATION D'UNE PERSONNE A LA RECHERCHE 26
5. DATA MANAGEMENT ET STATISTIQUES 27
5.1. RECUEIL ET TRAITEMENT DES DONNEES DE L'ETUDE 27
5.2. STATISTIQUES 28
6. SECURITE / EFFET INDESIRABLE 30
7. ASPECTS ADMINISTRATIFS ET REGLEMENTAIRES 31
7.1. DROIT D'ACCES AUX DONNEES ET DOCUMENTS SOURCE 31
7.2. DONNEES INFORMATISEES ET SOUMISSION A LA CNIL 31
7.3. AMENDEMENTS AU PROTOCOLE 31
7.4. REGLES RELATIVES A LA PUBLICATION 31
8. CONSIDERATIONS ETHIQUES 32
8.1. INFORMATION DU PATIENT ET CONSENTEMENT 32
8.2. GROUPE NANTAIS D'ETHIQUE DANS LE DOMAINE DE LA SANTE (GNEDS) 32
LISTE DES ANNEXES 33

INTRODUCTION

Le projet de recherche s'inscrit dans le cadre des démarches d'évaluation des thérapeutiques en santé mentale. Elle a pour cadre les unités infanto-juvéniles qui ont mis au point des « dispositifs intégratifs », tenant compte des avancées récentes des connaissances et offrant soins, éducation et approche pédagogique. L'évolution des enfants autistes bénéficiant de ces dispositifs intégratifs est étudiée, afin de déterminer la validité des dispositifs cités. La méthodologie s'appuie sur des études de cas en situation naturelle. Les cliniciens engagés se constituent en réseau, entre eux et avec les chercheurs, dans le cadre d'une étude multicentrique.

1. JUSTIFICATION DE L'ETUDE

1.1. POSITIONNEMENT DE LA RECHERCHE

Justification de l'étude
Les troubles envahissants du développement regroupent des situations cliniques diverses identifiées en huit catégories dans la CIM10 (Classification internationale des maladies). Les études épidémiologiques de Fombonne en 2009 (1) retiennent une prévalence des Troubles envahissants du développement (TED) de 6 à 7 pour mille, et de 2 pour mille pour l'autisme infantile alors que d'après l'expertise Inserm de 2002 (2). La prévalence des TED est de 27,3 pour 10000 et celle de l'autisme de 9 pour 10000, ces différences de prévalence sont en partie liées à des critères cliniques appliqués plus largement.

Les troubles envahissants du développement qui sont nommés dans la nouvelle classification DSM V, et le seront surement dans la CIM 11 à venir, « Troubles du Spectre Autistique », ont les mêmes critères diagnostics. L'augmentation de leur prévalence pourrait être liée à l'extension du concept de spectre, à une évolution des critères diagnostiques, à une meilleure connaissance des troubles de la part des professionnels et au développement du dépistage précoce. Par conséquent cette pathologie constitue un réel problème de santé publique qui mobilise les professionnels, les familles et les pouvoirs publics. Elle nécessite une dynamique de recherche et d'étude tant sur le plan étiologique et sur la compréhension des mécanismes sous-jacents que sur le plan de la prise en charge sous son double aspect thérapeutique et éducatif.

La prise en charge de l'autisme en pédopsychiatrie a beaucoup évolué ces dernières années dans ses références théoriques comme dans les modalités de soins qui en découlent. La nécessité que les enfants, présentant des troubles envahissants du développement, bénéficient de soins aussi intensifs et aussi précoces que possible y est clairement reconnue et consensuelle (3-4-5-6).

Au-delà des positions qui ont pu être dogmatiques sur ce sujet, la pluralité des facteurs en jeu dans l'avènement de la pathologie autistique amène à mettre en place des réponses multidimensionnelles qui ne préjugent pas de la part d'éléments génétiques et environnementaux (7-8-9). Le corpus théorique psychodynamique de la psychiatrie infanto-juvénile et l'expérience des soins, en s'appuyant sur l'analyse de la relation, sur la mise en récit et l'analyse des angoisses, a permis d'étayer les organisations institutionnelles et leurs médiations thérapeutiques (10-11) ; les connaissances et les méthodes de soins se sont enrichies des apports d'autres travaux. Les données des cognitions sociales, de l'empathie, de la théorie de l'esprit, de l'imitation (12), de l'équipement neurophysiologique (13) sont ainsi intégrées dans les modalités de traitement. Les spécificités de la pathologie autistique, telles que les modes particuliers de fonctionnement sensoriel, du partage des représentations et des émotions par exemple, sont prises en compte dans l'abord des jeunes patients. La description des unités de soins, dans toute leur diversité, témoigne de l'évolution de leurs dispositifs et de l'actualisation des connaissances.

Les unités de soins infanto-juvéniles ont mis au point des dispositifs multidisciplinaires coordonnés ou **dispositifs dits de soins intégratifs.**
Cette approche se définit par un ensemble d'interventions coordonnées proposées à un enfant appréhendé dans sa globalité et sa singularité, en relation avec ses parents et le milieu habituel de vie. Elle s'appuie sur des perspectives complémentaires : psychopathologiques, physiques et physiologiques et associe une pluralité d'interventions

(thérapeutiques, éducatives, pédagogiques) et une pluralité coordonnée d'intervenants. Ces dispositifs ont été interrogés ces dernières années, en particulier parce qu'ils n'avaient été ni suffisamment explicités ni évalués. Les recommandations de bonne pratique de la HAS en 2012 (14) insistent sur l'importance de développer la recherche clinique dans le but de connaître à long terme les effets des interventions proposées.
Le plan autisme 2013, rappelle l'importance de la poursuite des recherches sur le développement des prises en charge fondées sur l'évidence scientifique dont l'évaluation des traitements thérapeutiques. La fiche action 27 « favoriser la recherche clinique sur l'autisme » en définit les axes dont certains soulignent l'importance des interventions précoces.

L'évaluation des thérapeutiques en santé mentale est un problème d'une grande complexité (15-16-17) puisqu'elles ne reposent pas essentiellement sur des traitements médicamenteux mais qu'elles allient des approches éducatives, psychothérapeutiques et pédagogiques (18-19).

Elle nécessite une méthodologie qui prenne en compte, en situation naturelle, la diversité clinique des cas et leurs spécificités, les processus cliniques en jeu, leurs effets et les variables qui les sous-tendent (20-21-22-23). Dans le domaine de l'autisme, il est pratiquement impossible de définir des traitements efficaces de façon globale du fait de la diversité particulière des patients malgré des critères diagnostiques proches (24). Ces dernières années, deux études réalisées sur le sujet des thérapeutiques dans le domaine de l'autisme ont contribué à la mise en place de cette recherche :
- le travail de l'Association de Psychiatrie Infanto-Juvénile de Bretagne (APIJB) en association avec l'Association pour l'Evaluation et la Formation des Psychiatres (AEFCP) : évaluation du parcours de soins des enfants autistes en unités infanto-juvéniles- 2011
- le réseau de recherches sur les pratiques psychothérapiques (Unité Inserm Thurin, Falissard) (25-26) : études intensives de cas, associant à l'étude longitudinale des changements celle des médiateurs qui les sous-tendent et des modérateurs qui les conditionnent en partie. Elles montrent que ces méthodes peuvent aboutir à des résultats très intéressants (27) ; certains des outils du réseau de recherche sur les pratiques psychothérapiques et la réflexion de ses animateurs ont contribué à la mise en place de cette recherche.

Les soins sous forme de prises en charge intégratives, telles qu'elles sont exercées en pédopsychiatrie, n'ont pas fait l'objet de recherche clinique. Leur caractère coordonné, multidisciplinaire et précoce sera évalué, pour la première fois, dans leurs effets cliniques à partir de plusieurs services de psychiatrie infanto-juvénile répartis sur l'ensemble du territoire français.

Cette recherche permettra en outre une meilleure **connaissance des pratiques cliniques intégratives**, ce qui répondra à l'attente des professionnels et des usagers, des divers partenaires de santé publique, des tutelles ainsi que de l'HAS.

Elle permettra également le renforcement de la **formation** des équipes participantes par les échanges entre pairs, et par l'acquisition de connaissances contribuant au développement professionnel continu interdisciplinaire. Une formation aux outils d'évaluation sera préalable à l'engagement des équipes dans la recherche. Celles-ci se constitueront en **réseau** pour favoriser les échanges et les observations cliniques et l'homogénéisation des pratiques.

La Fédération Française de Psychiatrie-Conseil National de Psychiatrie (CNPP), avec ses collèges de pédopsychiatrie et de recherche, est partenaire de cette étude.

1.2. ORIGINALITE ET POINTS FORTS

A notre connaissance, c'est la première fois qu'une recherche multicentrique est proposée pour évaluer les pratiques intégratives en psychiatrie infanto-juvénile pour l'autisme.
Dans la méthodologie, nous n'avons pas introduit de groupe contrôle puisque l'objectif principal est l'évaluation des pratiques de soin.

Dans cette première recherche, les pratiques seront évaluées dans leur globalité afin de déterminer si elles entraînent une amélioration de l'état de l'enfant. A partir des résultats de cette recherche la question se posera alors de la comparaison entre deux pratiques avec un groupe contrôle afin de montrer l'efficacité d'une pratique par rapport à l'autre ce qui parait trop précoce avec nos connaissances actuelles.
Les pratiques intégratives, mises en place depuis les années 80 n'ont à ce jour jamais été évaluées à grande échelle. L'objectif de ce projet est donc d'évaluer ces pratiques dans leur globalité pour voir si elles entrainent une amélioration de l'enfant atteint sur 1 an. Cette pratique est globalement celle de la pédopsychiatrie publique. Prendre un groupe témoin de patients suivis par ces services en délivrant par exemple une partie (ou aucune) de la prise en charge ciblée sur un domaine et avec une seule technique alors que les enfants du groupe témoin relèverait d'une prise en charge globale pose une question éthique. Par ailleurs, d'autres études justifient que les profils sont tellement singuliers qu'avoir un groupe témoin n'a pas beaucoup de sens. En revanche l'évaluation principale sera contrôlée par deux professionnels neutres.

De nombreux services se sont engagés pour participer à cette recherche, tous n'ont pas pu être retenus, d'autres encore le souhaiteraient, témoignant ainsi de la volonté des professionnels de service public à évaluer leurs pratiques et leur impact sur l'évolution des enfants.

La perception parentale des progrès de leur enfant et de la qualité du service rendu sont essentiels dans les objectifs de cette étude.
L'appréciation subjective des parents de cette évolution sera comparée à celle des soignants avec les mêmes critères.

Cette recherche répond aux indications des recommandations de la HAS (mars 2012) et au 3éme plan autisme (2013-2017).
Le partenariat avec la FFP-CNPP ouvre sur une collaboration avec la Direction Générale de la Santé (DGS) pour la constitution d'un réseau et son animation afin de faciliter sa réalisation multicentrique.

1.3. BÉNÉFICES

1.3.1. Bénéfice individuel

Les enfants qui participeront à la recherche bénéficieront de thérapeutiques encadrées par des évaluations soigneuses et répétées.

1.3.2. Bénéfice collectif

Il s'agit d'une étude observationnelle multicentrique, prospective et ouverte. Elle s'applique en milieu habituel dans les unités de soins proposant un dispositif intégratif.

Cette recherche répond aux attentes exprimées par la société civile à travers le plan autisme 2013 sur la nature et l'efficience des soins en psychiatrie infanto-juvénile. En les rendant plus lisibles, en insistant sur la participation des parents, elle contribuera à l'amélioration de la confiance et de la collaboration entre professionnels et usagers.

Les professionnels qui participeront à la recherche approfondiront leurs connaissances dans le domaine clinique et dans celui de l'évaluation des patients présentant un autisme typique ou atypique par l'utilisation partagée d'échelles d'évaluation.
Les modalités thérapeutiques offertes aux enfants autistes pourront être mieux ajustées, donc optimisées.

1.4. BIBLIOGRAPHIE

1. Fombonne E.- 2009 (18)- Epidemology of pervasive developmental disorders- Pediatric.Res 2009 ; 65 (6) 591-8

2. Expertise collective Inserm. Troubles mentaux : dépistage et prévention chez l'enfant et l'adolescent. Paris : Inserm, 2002

3. Bursztejn C. Est-il possible de dépister l'autisme au cours de la première année ? ENFANCE 2009 ; 61(1) : 55-66.

4. Delion P, Beucher A, Bullinger A, Carel A, Charlery M, Golse B, et al. Les bébés à risque autistique. Ramonville Saint-Agne : Erès ; 2002.

5. Fédération Française de Psychiatrie, Haute Autorité de Santé-Recommandations pour la pratique professionnelle du diagnostic de l'autisme- Paris, Saint Denis la Plaine-FFP ; HAS- 2005.

6. Fernell E, Hevall A, Westerlund J, Carlsson LH, Eriksson M, Olsson M.B, Holm A, Norrelgen F, Kjellmer L, Gillberg C, Early intervention in 208 Swedish preschoolers with autism spectrum disorder. A prospective naturalistic study, Research in Developmental Disabilities, 2011, 32, 2092-2101.

7. 72e journées nationales de la SFPEADA-Psychopathologie et Neurosciences-5et 6 juin 2009- Lille

8. Bargiacchi A, Zilbovicius M. Imagerie cérébrale et autisme. ANAE 2008 ; 20(100) : 291-7.

9. Golse B L'autisme entre neurosciences et pychanalyse-enfance et Psy n°46-2010- dossier : l'autisme aujourd'hui ed Erès

10. Schmit G, Bouvet M, Hincky M.-O. Secteur de psychiatrie infantojuvénile. EMC - Pédopsychiatrie 2008:1-20 [Article 37-211-A-05].

11. Hochmann J. Soin institutionnel aux enfants et aux adolescents souffrant de troubles graves et précoces du développement (autismes et psychoses de l'enfance). EMC - Pédopsychiatrie 2009:1-9 [Article 37-210-A-10].

12. Nadel J.Imitation et cognition sociale dans l'autisme.NEURO PSY NEWS 2007 ; 6(3) : 124-7.

13. Barthelemy C, Huc Chabrolles M, Tripi G, Gomot M, Martineau J, Bonnet Brilhault F. Les compléments neurophysiologiques du diagnostic. ENFANCE 2009 ; 61(1) : 89-92. AUTISME

14. HAS-ANESM, Autisme et autres troubles envahissants du développement : interventions éducatives et thérapeutiques coordonnées chez l'enfant et l'adolescent mars 2012.

15. Bréchot C, Danion JM, *et al*. Méthodologie de l'évaluation en psychiatrie et en santé mentale. Colloque Inserm 2007. *Pour la recherche* 2007 ; 54 (3) : 1-12.

16. Kovess V, *et al*. Évaluation de la qualité en psychiatrie. Paris, Economica 1994, 318 p.

17. Kovess V, Lopez A, Pénochet JC, Reynaud M. Psychiatrie des années 2000, Organisations, Évaluations, Accréditation. Médecine-Sciences Flammarion, 1999, 306p.

18. Thurin JM. L'évaluation des psychothérapies, où en sommes-nous ? in Fischman G L'évaluation des psychothérapies et de la psychanalyse. Paris Masson 2009, p 101-116.

19. Thurin JM. Évaluation des effets des psychothérapies. *EMC (Elsevier Masson SAS, Paris), Psychiatrie*, 37-802-A-10, 2009.

20. Lambert MJ. Bergin & Garfield's Handbook of psychotherapy and Behavior Change. Sixth Edition. NY, J Wiley & Sons, 2013

21. Kraemer HC, Stice E, Kazdin AE, Offord DR, Kupfer DJ. 2001. How do risk factors work together? Mediators, moderators, independent, overlapping, and proxy-risk factors. Am. J. Psychiatry 158:848–56.

22. Thurin JM, Briffault X. Distinction, limites et complémentarité des recherches d'efficacité potentielle et d'efficacité réelle : nouvelles perspectives pour la recherche en psychothérapie. *L'Encéphale*, 2006 ; 32 : 402-12.

23. APA Presidential Task Force on Evidence-Based Practice. (2006). Evidence-based practice in psychology. *American Psychologist*, 61, 271–285.

24. Mesibov GB & Shea V. Evidence-Based practices and autism. *Autism* 2011 15 : 114-133.

25. Thurin JM, Falissard B, Danion JM. Réseau de recherches fondées sur les pratiques psychothérapiques. Pour la recherche 2008 ; 56

26. Thurin JM, Thurin M & Midgley N: Does participation in research lead to changes in attitudes among clinicians? Report on a survey of those involved in a French practice research network, *Counselling and Psychotherapy Research: Linking research with practice* 2012 ; 12:3, 187-193

27. Haag, Amenta, Messica, Thurin M et JM, Vassallo P, Lanteri A, Pasero L. Résultats préliminaires de 20 études intensives de cas du Pôle italien du RRFPP. *Pour la recherche* 2012 ; 73 :1-16.

2. OBJECTIFS ET CRITERES DE JUGEMENT

2.1. OBJECTIF ET CRITÈRE D'ÉVALUATION PRINCIPAL

2.1.1. Objectif principal

L'évaluation des pratiques de soin, à partir de l'évolution sur un an, d'enfants âgés de 3 à 6 ans, présentant un diagnostic d'autisme typique ou atypique (F 84-0 et F 84-1), soignés dans une unité de soins pratiquant les approches intégratives (Centre Accueil Thérapeutique à Temps Partiel CATTP, Hôpital de Jour).

2.1.2. Critère d'évaluation principal

Evaluation développementale dans le domaine de la cognition verbale et préverbale et des comportements inadaptés dans le domaine des expressions affectives des enfants entre le temps M0 (mois d'inclusion) et M12 (12e mois de suivi) selon l'échelle PEP 3.

2.2. OBJECTIFS ET CRITÈRES D'ÉVALUATION SECONDAIRES

2.2.1. Objectif(s) secondaire(s)

- Montrer l'efficacité des pratiques intégratives par l'évaluation de l'évolution globale des enfants appréciée selon des critères cliniques et des outils d'évaluation validés et sélectionnés.

- Evaluation par les familles :
    - de l'état de l'enfant et son évolution
    - de la qualité de la relation établie par l'équipe avec la famille.

- Contribuer à homogénéiser les pratiques de soins.

2.2.2. Critère(s) d'évaluation secondaire(s)

- Evolution globale de l'enfant appréciée par domaine de développement (langage et communication, sensori-moteur, interactions sociales, comportements, domaine cognitif, angoisses et émotions) à l'aide des échelles, ECA-R, CARS et des bilans orthophonique (ELO) et psychomoteur (Brunet-Lézine) à M0 et M12.

Domaine évalué	**Outils**
Langage et Communication	Bilan orthophonique ELO de Khomsi
Sensori moteur	Bilan psychomoteur de Brunet-Lezine
Interactions sociales	ECA-R 29 items
Comportements autistiques	ECA-R
Cognitif, globale	PEP3 131 items de développement et 43 de Comportement.
Angoisse, émotions et évaluation globale	CARS 15 items (ou sous échelles)

L'observation clinique structurée ou étude de cas comporte l'anamnèse de l'enfant, l'historique de sa prise en charge, les éléments du contexte de vie de l'enfant et de sa famille, les différents domaines de son développement appréciés dans la relation au soignant et le projet de soins proposé. Cet élément pourra faire partie d'une étude qualitative dont l'analyse pourra être réalisée par un psychologue des sciences humaines.

- Point de vue des familles apprécié par un questionnaire à M0 et à M12 : adaptation d'un outil mis au point par Tavistock Clinic and Portman NHS Trust (London).
Il comporte un questionnaire préalable à la prise en charge avec 30 questions, un questionnaire à la fin de la période de l'étude avec 44 questions, une appréciation de l'état de l'enfant « comment qualifierez-vous l'évolution de votre enfant ? » avec 10 items cotés de 0 à 4.

3. POPULATION ÉTUDIÉE

3.1. DESCRIPTION DE LA POPULATION

Quatre-vingt enfants âgés de 3 à 6 ans, seront recrutés pour cette recherche, dans l'année qui suit leur entrée en Hôpital de Jour ou Centre d'accueil thérapeutique à temps partiel.
Au préalable à cette entrée, s'effectuent la démarche diagnostique (dont le bilan somatique) et l'établissement de la relation thérapeutique qui demandent un ensemble d'actes qui s'étale sur un temps entre 3 et 6 mois.
Chez ces enfants le diagnostic d'autisme typique ou atypique est porté antérieurement à l'entrée dans la recherche.
Le nombre relativement limité d'enfants (80) entrant dans cette recherche correspond à l'exigence de délimitation d'une population relativement homogène définie par les catégories choisies dans le spectre autistique plus large.
Le recrutement de la population se fera par des équipes soignantes sélectionnées car utilisant les méthodes intégratives et ayant été formées aux outils de la recherche.

3.2. CRITÈRES D'INCLUSION

Critères d'inclusion généraux :
- Enfants de 3 à 6 ans avec diagnostic F84.0 et F84.1 selon les critères de la CIM-10. Le diagnostic devra avoir été fait selon les critères établis par les recommandations sur le diagnostic (FFP/HAS 2005).
- Enfants pris en charge dans des unités de soins répondant aux critères définis de pratiques intégratives.
- Enfants recevant un volume d'heures d'intervention entre deux et quatre demi-journées par semaine.
- Recueil écrit du consentement des parents.

3.3. CRITÈRES DE NON INCLUSION

Présence de co-morbidité telle qu'épilepsie, atteinte organique grave, somatique et sensorielle.

4. DÉROULEMENT DE L'ÉTUDE

4.1. MÉTHODOLOGIE GÉNÉRALE DE LA RECHERCHE

Il s'agit d'une étude prospective multicentrique, en situation naturelle, impliquant des équipes des secteurs de psychiatrie infanto-juvénile du territoire national qui partagent l'approche définie dans l'argumentaire de l'étude.
La recherche présente les caractéristiques suivantes :

- Etude portant sur les thérapeutiques
- Etude multicentrique nationale,
- Etude non contrôlée
- Etude en situation **naturelle** non randomisée,
- Etude **ouverte,**
- Etude **prospective,**

Notre méthodologie se fonde sur l'évaluation de l'enfant dans les différents domaines de son développement selon des outils validés et recommandés. Les évaluations ont lieu en début et fin d'étude et à des temps intermédiaires. Elles permettent notamment d'évaluer l'ajustement des pratiques intégratives aux particularités et aux besoins de l'enfant, l'évolution de celui-ci étant un élément de l'évaluation de l'efficacité des pratiques.
Le point de vue des familles, apporté par un questionnaire du début et fin d'étude, contribue au recueil des données. Le même questionnaire sera soumis aux professionnels référents de l'enfant permettant ainsi une comparaison des représentations subjectives de l'enfant que les uns et les autres se forgent au cours de la prise en charge.
Les enfants seront inclus après diagnostic validé selon les recommandations de la FFP et la HAS de 2005 et après information et consentement écrit des parents. La première évaluation de l'enfant par domaines sera réalisée à M0 à partir d'échelles validées, le profil de l'enfant ainsi défini sera la base pour l'élaboration du projet de soin. Elle comporte également un questionnaire aux familles, aux professionnels et une évaluation interjuge. La même évaluation sera réalisée à M12.
Des évaluations intermédiaires à M3, M6, M9 s'appuieront sur une échelle de comportement.
Les médecins responsables des centres investigateurs participeront à des réunions dans le but de s'approprier la méthodologie, d'acquérir un niveau de formation suffisant, de les transmettre et de soumettre au groupe ainsi constitué les questions qui se présentent au fur et à mesure de l'avancée du projet. Une réunion tous les quatre mois serait nécessaire avec la participation de la FFP-CNPP.

4.2. TECHNIQUES D'ÉTUDES ET D'ANALYSES

4.2.1. Description détaillée des paramètres d'évaluation

Les instruments d'évaluation

1- Profil psycho-éducatif révisé (PEP 3) de Schopler (1994)

Le PEP est un outil qui permet de définir le profil cognitif et comportemental de l'enfant autiste dans le but d'adapter les propositions éducatives au plus près des particularités de l'enfant.
Il permet d'évaluer les capacités actuelles et en émergences dans sept domaines : imitation, perception, motricité globale, coordination oculomanuelle, performance cognitive, cognition verbale. Il évalue le comportement dans les domaines suivants : relations et affect, jeu et intérêt pour le matériel, réponses sensorielles et langage.
Il est adapté à des enfants ayant un niveau au-dessous de 7 ans.
Il permet de visualiser les points forts et les points faibles et ceux en émergence. Il comporte 113 items de développement et 43 de comportement. Les différentes épreuves s'appuient sur du matériel de jeu et du matériel pédagogique, sont présentés à l'enfant au cours des séquences de jeu structuré. L'examinateur observe évalue et enregistre les réactions de l'enfant, les cotations obtenues sont reparties dans sept domaines de développement et quatre de comportement. Le PEP 3 comporte 3 types de cotation : réussite, échec, émergence. En ce qui concerne le comportement la cotation indique : approprié, léger et sévère.
C'est à partir de cet instrument que sera évaluée l'évolution de l'enfant entre M0 et M12. Le psychologue de l'étude réalisera cette évaluation ainsi que deux autres psychologues indépendants. Les résultats qui seront pris en compte à M0 et M12 seront ceux des psychologues indépendants.

2- ECA-R : Évaluation des comportements autistiques (version révisée) de G. Lelord et C. Barthélémy

Cette échelle a été validée dans le service de pédopsychiatrie de Tours. Elle comporte 29 items cotés de 0 à 4 en fonction de leur fréquence d'apparition. Elle permet d'explorer l'enfant autiste dans différents domaines de son comportement : retrait social, troubles de la communication verbale et non verbale, adaptation aux situations environnementales, troubles du tonus, motricité perturbée, réactions affectives, troubles des grandes fonctions instinctives, troubles de l'attention, des perceptions et des fonctions intellectuelles. Elle indique, pour les différents comportements observés, les variables spontanées au cours du temps et les améliorations induites par les thérapeutiques et les rééducations.
A partir des cotations régulières de l'échelle, des profils symptomatiques sont obtenus pour un enfant. Ce profil peut être suivi sur plusieurs mois. Il est possible d'analyser l'évolution de ce profil, symptôme par symptôme, en tenant compte des différents facteurs qui ont éventuellement influencé cette évolution.
Les données quantifiées recueillies à partir de l'échelle sont par ailleurs utilisables en tant que variables cliniques pour la recherche d'éventuelles relations avec d'autres variables. Il est ainsi possible de suivre l'évolution de la "déficience relationnelle et de "l'insuffisance modulatrice" à partir de scores récapitulatifs spécifiques.

3- Echelle d'évaluation de l'autisme infantile Childood Autism Rating Scale de Schoopler et al (1980) (CARS)

Il s'agit d'une échelle d'évaluation et d'intensité.

Elle est composé de 14 sous échelles ou domaines d'évaluation : les relations sociales, l'imitation, les réponses émotionnelles, l'adaptation aux changements, l'utilisation du corps, des objets, les réponses visuelles, auditives, goût, odorat, toucher, peurs et anxiété, communication verbale et non verbale, niveau d'activités, niveau intellectuel. Une 15ème échelle mesure l'impression générale de l'examinateur concernant la gravité de l'autisme. Chaque sous-échelle est décrite dans le manuel avec les principaux éléments à retenir. Chaque item est coté de 1 à 4, 4 étant le sévèrement anormal. Le total des points varie entre 15 et 60, la note seuil est 30. De 30 à 36,5 on est dans le registre d'un autisme modéré, à partir de 37 on est dans le registre de l'autisme sévère.
Cette échelle a fait l'objet de nombreuses études de validation, elle peut être utilisée à partir de 24 mois, au cours des séances de jeu et de vie quotidienne. Elle peut être remplie par plus d'un observateur ce qui permet de vérifier l'accord inter juge.
C'est un outil intéressant et formateur à l'observation clinique dans les services de soins.

4- Evaluation orthophonique

L'évaluation orthophonique doit permettre de regrouper toutes les informations concernant la manière dont l'enfant communique afin d'établir un profil de ses moyens et de ses fonctions de communication. Il s'agit de mettre en lumière les difficultés mais aussi les émergences et les compétences de l'enfant.
Cette évaluation orthophonique est complexe, notamment en raison:
- du peu d'outils spécifiques existants, qui oblige chaque orthophoniste à évaluer compétences et déficits de l'enfant essentiellement par l'observation personnelle.
- de la nature même de la pathologie (troubles attentionnels, comportementaux et absence de langage) qui rend difficile l'évaluation de la compréhension.

Il est important d'évaluer les prérequis à la fonction de communication par :
- Le regard
- L'imitation gestuelle et verbale, le jeu de faire-semblant
- L'attention conjointe
- Les expressions faciales, mimiques et praxies.
- Les gestes symboliques, les pointage proto-impératif et proto-déclaratif et gestes sociaux.
- Les vocalisations

Evaluation du langage oral - ELO

Ce test a été élaboré dans les années 80 et régulièrement révisé par A. Khomsi, professeur de psychologie et de linguistique.
Cette batterie de six épreuves est destinée à décrire et évaluer, de façon fine, diverses composantes de la compétence orale.
Elle est composée des épreuves suivantes : Vocabulaire, Phonologie, Compréhension et Production Linguistique, aussi bien en réception qu'en production.
Une analyse clinique fine de ces domaines permet d'établir des profils individuels et d'identifier la composante sur laquelle il y a lieu d'intervenir, directement ou indirectement.
- Vocabulaire comporte deux épreuves : Lexique en Réception (LexR) (20 planches). Lexique en Production (LexP) (série de 50 images) (série de 10 images pour les plus jeunes).
- Compréhension comporte deux sous-épreuves selon l'âge de l'enfant.
- Phonologie avec une épreuve de Répétition de Mots (RepM).
- Production linguistique. Deux épreuves sont utilisées pour explorer les compétences morphosyntaxiques de l'enfant : Répétition d'Enoncés pour les enfants de maternelle.

Production d'Enoncés dans laquelle l'enfant complète une phrase. Cette épreuve met en évidence les dysfonctionnements linguistiques impliquant le traitement de la morphologie ou de la syntaxe, ainsi que les dysfonctionnements pragmatiques dans lesquels l'enfant ne tient pas compte du contexte linguistique et pragmatique proposé.

Des profils construits à partir des notes globales, permettent d'identifier les caractéristiques particulières du fonctionnement de chaque enfant. Ils sont complétés par des profils spécifiques à chaque épreuve, construits à partir de diverses sous-notes calculées qui permettent d'affiner l'analyse des cas. La plupart des épreuves sont organisées de telle sorte qu'un critère d'arrêt permet de ne pas utiliser l'ensemble des items. Dans ce cas, les items non utilisés peuvent l'être en cours ou en fin de prise en charge.

Pour chaque enfant, son profil de départ sera retenu et réévalué au temps M12.

5- Le Brunet – Lézine : évaluation du développement psychomoteur de l'enfant

Le Brunet-Lezine est un test élaboré dans les années 50 révisé à plusieurs reprises dont la dernière en 2001. Il est utilisé couramment dans les services de psychiatrie infanto-juvénile par les psychomotriciens.
Il comprend un ensemble d'épreuves ordonnées par âge et une série de questions destinées aux parents. Ces épreuves permettent le calcul d'un quotient de développement.
Cet outil est adapté aux enfants de 0 à 5 ans. L'analyse des résultats permet le calcul d'âges de Quotients de Développement (QD). Quatre sont partiels et concernent les quatre domaines évalués (nommés ci-dessous), le dernier est global.
Les quatre secteurs de développement sont :
- le contrôle postural et la motricité (noté P) : épreuves de locomotion et de contrôle postural de l'enfant en position dorsale, ventrale, assise et debout ;
- la coordination oculomotrice et les conduites d'adaptation par rapport aux objets (noté C) : étude de la préhension, du comportement de l'enfant face aux objets, de l'imitation de gestes ;
- le langage (noté L) : étude du langage dans ses fonctions de compréhension et d'expression ;
- les relations sociales (noté S) : étude des relations sociales englobant l'adaptation à différentes situations sociales et quotidiennes, la prise de conscience de soi et celle d'autrui.
Pour chaque âge, il y a 10 épreuves. Mais le nombre d'épreuves dans chacun des 4 secteurs du développement varie d'un âge à l'autre, ce qui reflète les comportements typiques de chaque âge. L'évaluation s'effectue en termes de réussite ou d'échec à l'épreuve.
Ce test dure environ 40 minutes. Il se compose de 17 niveaux d'âge entre 1 et 30 mois. Le score obtenu permet d'établir un Quotient de Développement (QD).
Bien que ce test ne soit pas spécifique de la pathologie autistique, il prend en compte des critères suffisamment sensibles pour ce type de pathologie. Il donne également une vision relativement pertinente du niveau des capacités développementales de l'enfant.

6- Questionnaires Famille – Questionnaires Professionnels (Annexe 3, Tableau 1, 2, 3 et 4)

Il nous est apparu important de recueillir le point de vue des familles par un questionnaire au début et à la fin de l'étude. Dans ce but, nous avons adapté un outil mis au point par la Tavistock Clinic and Portman NHS Trust (London).

Ce centre de psychiatrie et psychothérapie infanto-juvénile du Royaume-Uni (Londres) comporte une unité spécialisée dans la prise en charge des enfants autistes. Les professionnels de ce service ont élaboré deux questionnaires pour prendre en compte les observations des parents au sujet de :

- la perception des symptômes de l'enfant,
- l'évolution des manifestations,
- l'appréciation de la qualité de la relation des professionnels avec eux.

Il comporte un questionnaire préalable à la prise en charge avec 30 questions.
Le questionnaire à la fin de la période de l'étude est composé de 44 questions.

Il comporte un questionnaire préalable à la prise en charge avec 30 questions, concernant les principales inquiétudes des parents au sujet des principales manifestations de l'enfant à l'origine de la demande de prise en charge. Il détaille le point de vue des parents sur le développement de l'enfant principalement dans le domaine de la communication sociale et imaginaire.
Un questionnaire à la fin de la période de l'étude avec 44 questions fait le relevé de l'intensité, de la durée et la qualité du traitement et des liens entre partenaires. Il reprend l'évolution de l'enfant dans les différents domaines ainsi qu'une appréciation générale de l'état de l'enfant faite à partir de 10 items cotés de 0 à 4.
Ce questionnaire sera soumis aux parents aux temps M0 et M12.
Ce même type de questionnaire sera également soumis aux professionnels aux temps M0 et M12 afin de réaliser une comparaison des points de vue sur l'enfant.

7- Inventaire des facteurs externes (Annexe 3, Tableau 5)

Les évènements significatifs survenant durant l'année de la recherche, concernant l'enfant ou sa famille, sont notés. L'impact sur le comportement et l'évolution de l'enfant seront analysés.

Dans le cadre des objectifs secondaires de cette recherche il est prévu de contribuer à homogénéiser les pratiques de soins.

8- Contribution à la formation des professionnels, à l'évaluation et à l'harmonisation de leurs pratiques (Annexe 3, Tableau 6)

Cette recherche contribue à la formation des professionnels opérant dans les services de psychiatrie infanto-juvénile grâce à une analyse plus fine du profil de l'enfant réalisé au cours des différentes étapes par l'observation clinique, l'appropriation d'outils d'évaluation et la collaboration avec les parents.

Une grille d'évaluation sur la connaissance des échelles utilisées est soumise aux professionnels au début et à la fin ainsi qu'une appréciation de leur intérêt dans la prise en charge thérapeutique.
Ce renforcement de la formation contribue à l'homogénéisation des pratiques professionnelles dans le domaine de l'autisme qui est un des objectifs secondaires de l'étude.

4.2.2. Description des techniques et analyses

1- Approche intégrative

Une approche intégrative se définit par un ensemble d'interventions coordonnées proposées à un enfant appréhendé dans sa singularité et sa globalité, en relation avec les parents. Elle promeut le maintien de l'enfant dans son milieu habituel de vie, incluant celui-ci comme support du projet thérapeutique et éducatif.

Cette approche s'appuie sur une mise en relation de perspectives complémentaires : psychopathologique (psycho-dynamique, développementale et cognitive), physique et physiologique (avec tous les aspects de l'équipement somatique de l'enfant : sensoriel, neurologique, biologique, génétique…) conduisant à l'élaboration d'une compréhension partagée entre les professionnels et les parents et à un projet de soin individualisé.

Elle associe :
- La pluralité des interventions : thérapeutiques (constituées par des ateliers ou des groupes thérapeutiques réalisés par des infirmiers et/ou des éducateurs, des psychothérapies…), rééducatives, éducatives, pédagogiques (au sein de l'école et/ou de l'unité d'enseignement du lieu institutionnel).
- La pluralité des intervenants

Les interventions prennent en compte différents domaines ou dimensions : sensori-moteur - communication et langage - émotion, angoisse et comportements - cognitif/pédagogie - socialisation (autonomie distale) - éducatif (autonomie proximale) - ressources familiales - suivis somatique et pharmacologique…
La coordination des interventions et des intervenants s'exerce à la fois avec un référent psychiatre et un référent autre (éducateur, infirmier) qui sont engagés auprès de l'enfant et de sa famille et son garants de la continuité du projet (cette conception de la coordination se distingue d'une coordination exercée par la MDPH, ou par les parents de l'enfant eux-même, ou encore sur le mode d'un échange non organisé entre diverses équipes ou professionnels). Elle s'appuie au niveau intra institutionnel sur des synthèses cliniques, véritables espaces de connexion et d'élaboration du matériel clinique issu des différentes actions et médiations thérapeutiques, et sur des articulations extra institutionnelles (réunion avec l'institution scolaire…).
Les interventions et le projet de soin individualisé s'appuient sur l'observation clinique structurée.

1- Trame du compte-rendu de l'observation clinique structurée (Annexe 3, Tableau 7)

Dans la pratique clinique en psychiatrie infanto-juvénile, selon l'approche intégrative, l'ensemble des professionnels met en commun ses observations pour arriver à une synthèse clinique. Elle rend compte de l'évolution globale de l'enfant articulant une perspective subjective et une perspective objective.

Ces interventions s'organisent en un projet individualisé qui intègre les particularités et les besoins de l'enfant.

2- Le projet de soin individualisé (Annexe 3, Tableau 8)

Le projet de soin individualisé se construit à partir des éléments recueillis au cours de l'observation clinique structurée, des évaluations dans les différents domaines, de l'observation des parents et des autres intervenants.
Il définit les objectifs de la prise en charge et les moyens pour le réaliser. Le terme de projet de soin individualisé insiste sur les aspects dynamiques et révisables et sur l'adéquation personnalisée de celui-ci au profil et aux besoins spécifiques de cet enfant. Le projet intègre

les trois volets : le soin, l'éducatif et le pédagogique en conformité au code de la santé publique et aux recommandations de l'HAS.
Le projet comporte les interventions que l'enfant reçoit en référence au tableau des activités qui explicite les objectifs thérapeutiques, éducatifs et pédagogiques des différentes interventions proposées.
La place de chaque professionnel est définie, ainsi que leur coordination, les modalités d'articulation avec les familles et les autres intervenants. La révision régulière du projet est notée.

3- Descriptif des interventions thérapeutiques et éducatives (Annexe 3, Tableau 9)

Le projet de soins individualisé comprend des interventions individuelles et des interventions en petits groupes.

Les ateliers thérapeutiques et les activités proposés utilisent des supports très diversifiés. De manière générale, ils visent le développement de compétences nouvelles et d'expériences inédites et ils contribuent à l'installation de la relation par l'étayage des soignants. Le dénominateur commun est l'attention portée aux manifestations de l'enfant, leur prise en compte, leur décodage dans le but d'aider l'enfant à acquérir des outils pour mieux se percevoir lui-même et le monde environnant.

On distingue au sein des unités :

- Les activités du quotidien (l'accueil et le départ, le repas ou la collation, l'habillage, les temps de transition, les sorties, l'hygiène corporelle et l'éducation sphinctérienne, etc). Ces activités contribuent au repérage temporel et psychosocial de l'enfant, à la réduction de l'angoisse associée à son rapport au monde et aux autres, et à son éducation. Elles apportent un matériel clinique sur le fonctionnement de l'enfant. Elles donnent lieu à des observations à partager avec les familles dans le but de trouver des stratégies communes.
On doit aussi garder présent à l'esprit que des apprentissages incidents s'opèrent et qu'une seule activité permet de travailler plusieurs dimensions.

- Les ateliers ou groupes thérapeutiques ou médiations thérapeutiques.
Ils permettent de prendre en compte le langage du corps, de l'affect, du sensori-moteur ; ils visent à l'acquisition des capacités de représentation et des compétences sociales et cognitives. Les ateliers se déroulent suivant un cadre précis : à partir d'une indication fondée sur des objectifs définis ; ils ont lieu dans un temps et un espace réguliers, pour une durée déterminée et reconductible ; ils sont animés par des professionnels référents de cet atelier.

- Les interventions individuelles.
Il peut s'agir d'interventions de l'infirmier ou de l'éducateur (sur les lieux de soin ou de vie de l'enfant), de psychomotricité, d'orthophonie, de psychothérapie et d'intervention pédagogique. Certaines interventions se réalisent en présence d'un ou des parents, avec un ou plusieurs professionnels.

- Les entretiens de la famille avec le pédopsychiatre et/ou le psychologue et/ou un autre professionnel de l'équipe représentent des moments clés permettant l'ajustement du projet, le partage des représentations et différents niveaux d'accompagnement.

- Les articulations
Un travail de lien est constamment recherché entre les professionnels concernés par l'enfant sous forme de rencontres, réunions cliniques, etc.

Pour les besoins de l'étude, et de manière volontairement systématisée, les médiations seront décrites en les corrélant au(x) domaine(s) fonctionnel(s) qu'elles permettent d'aborder avec l'enfant (cf tableau, annexe).

4.3. CALENDRIER DE L'ÉTUDE

Actions	M0 (Visite d'inclusion)	M2	M3	M6	M9	M12
Etape diagnostique = Diagnostique validé	X					
Information des parents	X					
Première évaluation (échelles CARS, PEP-3, ECA-R, bilans orthophonique, psychomoteur, inventaire des facteurs externes, questionnaires famille et professionnels, évaluation inter-juge, Cr de l'observation clinique structurée)	X					X
Tableau du projet de soins individualisé	X					
Indicateurs de changement de comportement (ECA-R)			X	X	X	
Dernière évaluation = Sortie d'étude						X

4.4. CRITÈRES D'ARRÊT PRÉMATURÉ DE LA PARTICIPATION D'UNE PERSONNE À LA RECHERCHE

Les critères d'arrêt prématuré à la recherche sont les suivants : enfants quittant l'institution où se déroule la recherche, parents décidant d'interrompre l'inclusion de l'enfant dans la recherche.

Pour les modalités et la durée du suivi des personnes ayant arrêté prématurément l'étude, se reporter à la section statistique.

5. DATA MANAGEMENT ET STATISTIQUES

5.1. RECUEIL ET TRAITEMENT DES DONNÉES DE L'ÉTUDE

5.1.1. Recueil des données

Un cahier d'observation (CRF) papier sera créé par patient. Toutes les informations requises par le protocole doivent être fournies dans le CRF. Il doit comprendre les données nécessaires pour confirmer le respect du protocole et toutes les données nécessaires aux analyses statistiques, et déceler les écarts majeurs au protocole.
La personne responsable du remplissage des CRF dans chaque centre sera le pédopsychiatre, investigateur principal.
Une fois complétés, les pages des CRF papiers seront expédiés au fur et à mesure par la poste, et seront centralisées à Nantes pour la saisie des données dans la base de données électronique « Clinsight» par le technicien d'essai clinique (TEC).

Des règles de remplissage seront définies.
Les données devront être copiées de façon nette et lisible.
Les données manquantes seront notifiées.
Les données erronées seront clairement barrées et les nouvelles données copiées à coté avec la date et les initiales du correcteur.

5.1.2. Codage des données

En signant ce protocole l'investigateur principal et l'ensemble des co-investigateurs s'engagent à maintenir confidentielles les identités des enfants (et des parents) qui ont participé à l'étude.

La transmission des données d'une personne à des fins de recherche ne sera dès lors possible que sous réserve de l'apposition d'un système de codage ; la présentation des résultats de la recherche doit exclure toute identification directe ou indirecte.

Le code patient se composera des initiales de l'enfant (première lettre du prénom et première lettre du nom), de son mois et année de naissance et du numéro du centre (de 1 à 17). Ce code sera la seule information qui figurera sur le cahier d'observation (CRF) et qui permettra de rattacher à posteriori le CRF au patient.
Le responsable de la recherche est également tenu de coder les données patients sur tous les documents qu'il pourrait avoir en sa possession (compte-rendus d'examens d'imagerie, de biologie, …) qui seraient joints au CRF.

5.1.3. Traitement des données

La collecte des données cliniques reposera sur la mise en place d'une base de données clinique et la création de masques de saisie à l'image du cahier d'observation en conformité avec le protocole et les réglementations actuellement en vigueur.

La structure de la base de données et des écrans de saisie sera approuvée par le responsable de la recherche.
La saisie des données sera réalisée dans la base de données Clinsight, logiciel développé par le data Manager du Département promotion du CHU de Nantes. Cet e-CRF sera accessible par un login et un mot de passe depuis le site internet https://www.hugo-online.org/csonline.

5.2. STATISTIQUES

Nom et coordonnées du responsable de l'analyse : Jean-Benoit HARDOUIN, EA 4275 « Biostatistique, Pharmacoépidémiologie et Mesures subjectives en santé », Université de Nantes.

5.2.1. Description des méthodes statistiques prévues, y compris du calendrier des analyses intermédiaires prévues

Chaque score issu de chaque échelle utilisée et les données recueillies seront toutes décrites par la moyenne et l'écart-type pour les variables continues et par les fréquences pour les données qualitatives.

Afin de mesurer l'efficacité des pratiques intégratives, les écarts entre les scores mesurés à la baseline et à la visite à M12 seront décrits par des intervalles de confiances à 95% obtenu à l'aide d'un modèle linéaire avec un effet aléatoire sur le centre (afin d'ajuster les résultats sur le centre). Pour l'échelle ECA-R donnant lieu à des mesures répétées plus de 2 fois, l'évolution sera en outre modélisée par des modèles linéaires à effet aléatoire, permettant de tenir compte de la répétabilité des données.

5.2.2. Justification statistique du nombre d'inclusions

Etant donné le caractère descriptif et innovant de la recherche, il a été impossible de déterminer un nombre de sujets nécessaires. Le choix d'inclure 80 enfants a donc été fait à partir de la faisabilité de l'étude, en incluant le plus grand nombre possible d'enfants.

5.2.3. Degré de signification statistique prévu

Le degré de signification statistique prévu est de 5%.

5.2.4. Critères statistiques d'arrêt de la recherche

Non applicable

5.2.5. Méthode de prise en compte des données manquantes, inutilisées ou non valides

Les données manquantes ne seront pas imputées. Néanmoins, en cas de données manquantes nombreuses (plus de 10%), des analyses de sensibilités seront réalisées afin d'appréhender l'impact des données manquantes sur les résultats, en imputant les données manquantes par différents scenarii (moyenne des autres patients, pire cas, meilleur cas…).

5.2.6. Gestion des modifications apportées au plan d'analyse de la stratégie initiale

La stratégie d'analyse ne sera pas modifiée, néanmoins, il sera possible de réaliser des analyses complémentaires non prévues initialement.

5.2.7. Choix des personnes à inclure dans les analyses

Tous les enfants inclus dans l'étude seront analysés.

6. SÉCURITÉ / EFFET INDESIRABLE

La survenue d'un Effet Indésirable lié à la prise en charge du patient au cours du présent protocole donnera lieu à une déclaration dans le système de vigilance adéquat (pharmacovigilance, biovigilance, hémovigilance, matériovigilance, etc…).

7. ASPECTS ADMINISTRATIFS ET RÉGLEMENTAIRES

7.1. DROIT D'ACCÈS AUX DONNÉES ET DOCUMENTS SOURCE

Les données médicales de chaque patient ne seront transmises qu'à l'organisme de rattachement de la personne responsable de la recherche ou toute personne dûment habilitée par celui-ci dans les conditions garantissant leur confidentialité.
Le cas échéant, l'organisme de rattachement de la personne responsable pourra demander un accès direct au dossier médical pour vérification des procédures et/ou des données de la recherche, sans violer la confidentialité et dans les limites autorisées par les lois et régulations.

7.2. DONNÉES INFORMATISÉES ET SOUMISSION À LA CNIL

Les données recueillies au cours de l'étude seront conservées dans un fichier informatique respectant la loi « informatique et libertés » du 6 janvier 1978 modifiée en 2004.

Le protocole sera soumis à l'avis du CCTIRS, et le traitement informatisé fera l'objet d'une demande d'autorisation auprès de la CNIL.

7.3. AMENDEMENTS AU PROTOCOLE

Le protocole modifié devra faire l'objet d'une version actualisée datée.
La note d'information devra faire l'objet de modification si nécessaire.

7.4. RÈGLES RELATIVES À LA PUBLICATION

Une copie de la publication sera remise au CHU de Nantes, responsable de la recherche de l'étude, qui sera nécessairement cité. Les auteurs seront déterminés au prorata du nombre de patients inclus. L'investigateur coordonnateur établit la liste des auteurs.

Toute publication mentionnera le soutien financier par la DGOS par la mention suivante : « This study is funded by the "Département général de l'offre de soins" (DGOS, French Ministry of Health) »

8. CONSIDÉRATIONS ÉTHIQUES

8.1. INFORMATION DU PATIENT ET CONSENTEMENT

L'investigateur s'engage à informer les parents de façon claire et juste du protocole (note d'information en annexe 2). La pathologie de l'enfant et son âge ne permettant pas une compréhension claire du protocole, seuls les parents seront informés de l'étude. Dans le meilleur des cas, une information orale très simplifiée pourra être délivrée à l'enfant. L'investigateur remettra aux parents un exemplaire de la note d'information avec la demande de consentement. Celle-ci précisera la possibilité pour les parents de refuser la participation de leur enfant à la recherche.

Le consentement écrit d'au moins un des deux parents sera recueilli avant l'inclusion de leur enfant dans l'étude.

L'investigateur notera dans le dossier du patient que les parents de celui-ci ont bien été informés oralement. Il joindra un exemplaire du consentement signé pour que leur enfant participe à la recherche dans le dossier et donnera l'autre aux parents.

8.2. GROUPE NANTAIS D'ETHIQUE DANS LE DOMAINE DE LA SANTE (GNEDS)

Le protocole et la note d'information et consentement seront soumis à l'avis du groupe nantais d'Ethique dans le Domaine de la Santé.

LISTE DES ANNEXES

- Annexe 1 : Listing investigateur
- Annexe 2 : Consentement parent
- Annexe 3 : Tableaux et questionnaires utilisés pour les pratiques intégratives
- Annexe 4 : Détail de la grille budgétaire et devis

ANNEXE 1 : LISTING DES INVESTIGATEURS

Civilité	Nom	Prénom	Ville	Pays	Hôpital	Courriel	Tél.	Spécialité	RPPS
M.	Perrier	Edmond	Haguenau	France	HDJ Les Marmousets	edmond.perrier@ch-epsan.fr	03.88.64.61.09	Pédopsychiatrie	10003944096
Mme	Royer	Jacqueline	Fontenay sous bois	France	CATTP La Marelle	d.rocherabreau@hopitaux-st-maurice.fr	01.43.28.77.78	Pédopsychiatrie	10000556661
Mme	Roos-Weil	Fabienne	Paris	France	HDJ La Pomme	fabrwl@wanadoo.fr	01.40.37.50.99	Pédopsychiatrie	10001640514
M.	Giulioni	Filippo	Brienne Le Chateau	France	EPSM de l'Aube	filippo.giulioni@ch-brienne.fr	03.25.92.36.36	Pédopsychiatrie	10001737401
Mme	Pelloux	Anne-Sylvie	Paris	France	CMP de l'Horloge	annesylviep@orange.fr	01.42.77.15.80	Pédopsychiatrie	10001269991
Mme	Virevialle	Catherine	Nemours	France	CH de Nemours	c.virevialle@ch-nemours.fr	01.64.45.19.00	Pédopsychiatrie	10000699420
Mme	Squillante	Maria	Brest	France	CHU de Brest	maria.squillante@chu-brest.fr	02.98.41.54.55	Pédopsychiatrie	10002614823
M.	Pourrat	Alain	Bron	France	CH du Vinatier	alain.pourrat@ch-le-vinatier.fr	06.61.74.14.04	Pédopsychiatrie	10003007563
Mme	Latinis-Héritier	Isabelle	Lagny	France	CH Lagny	i.latinis-heritier@ch-lagny77.fr	01.61.10.61.10	Pédopsychiatrie	10000705953
Mme	Guettier	Blandine	Gonesse	France	CH Gonesse	blandine.guettier@ch-gonnesse.fr	01.34.53.21.07	Pédopsychiatrie	10001323632
Mme	Marchal	Anne	Niort	France	CH Niort	anne.marchal@ch-niort.fr	05.49.78.38.10	Pédopsychiatrie	10002710357
M.	Moreau	Françis	Mons en Baroeul	France	CMP pour enfants et adolescents	moreaufrc@numericable.fr	03.20.84.22.84	Pédopsychiatrie	10002231925
Mme	Pernel	Anne-Sophie	Sainte-Gemmes-sur-Loire	France	Centre Santé Mentale Angevin	anne-sophie.pernel@ch-cesame-angers.fr	02.41.80.77.33	Pédopsychiatrie	10100075935
Mme	Carpentier	Laurence	Labege	France	ARSEAA-Centre de guidance infantile	carpentier@arseaa.org	05.61.62.60.35	Pédopsychiatrie	10002863206

Mme	Montreynaud	Valérie	Paris	France	Secteur I09 CMP	valerie.montreynaud@gmail.com	01.42.29.45.45	Pédopsychiatrie	10000362805
Mme	Deyon	Dominique	Paris	France	Unite de Soins Precoces HJ Rene Diatkine ASM13	dominique.deyon@asm13.org	01.40.77.43.65	Pédopsychiatrie	10000373778
Mme	Poullaouec	Joëlle	Bouguenais	France	CH de Daumezon	joelle.poullaouec@ch-daumezon.fr	02.51.82.93.70	Pédopsychiatrie	10002530466

ANNEXE 2 : CONSENTEMENT PARENT

Note d'information pour la participation à la recherche
« Evaluation clinique des pratiques intégratives en unités de soins infanto-juvéniles pour des enfants présentant un autisme typique ou atypique **»**

Médecin investigateur
Nom : …Dr N.GARRET-GLOANEC………………………………………………………………………………..
Service : …Pédopsychiatrie II *, Hôpital Saint Jacques – CHU de Nantes* ………………………..
Adresse : …85 rue Saint-Jacques 44093 Nantes Cedex 1
Téléphone : …02 28 08 84 90……………………………………………..

Responsable de la recherche
Nom : CHU de Nantes
Adresse : 5 allée de l'île Gloriette, 44 093 NANTES
Principaux contacts : Secrétariat Direction de la recherche
Téléphone : 02 53 48 28 35 (secrétariat Direction de la recherche)

Ce document est remis aux parents de l'enfant
Un exemplaire est conservé dans le dossier médical

Madame, Monsieur,

Le service de Pédopsychiatrie du Centre Hospitalier et Universitaire de Nantes effectue une recherche sur l'évaluation clinique des pratiques intégratives en unités de soins infanto-juvéniles pour des enfants présentant un autisme typique ou atypique. Cette recherche s'attache à préciser les modalités de la prise en charge des enfants de 3 à 6 ans présentant un autisme typique ou atypique et recevant des soins en psychiatrie infanto-juvénile sur une durée hebdomadaire entre deux et quatre demi-journées. Elle comporte un bilan au début de l'inclusion qui passe en revue toutes les différentes dimensions de l'enfant (sensori-moteur, communication, socialisation, autonomie…). Elle se déroule sur une année, des évaluations intermédiaires ont lieu à trois, six et neuf mois axée sur une échelle de comportement autistique. Au terme de l'année, un nouveau bilan complet sera effectué. Nous vous demanderons également de répondre par écrit à un questionnaire au début et à la fin de la recherche recueillant votre point de vue sur les différents aspects de votre enfant, la qualité de la prise en charge et des relations établies entre vous et les professionnels. L'objectif est de contribuer à l'évaluation de l'efficacité des soins tels qu'ils sont proposés au sein des services, de le rendre plus lisible, de les améliorer et de favoriser la formation et l'échange entre professionnels des différents centres investigateurs.

Cette recherche, sous la responsabilité du CHU de Nantes, concerne dix-sept autres centres investigateurs sur l'ensemble de la France, elle est réalisée en collaboration avec la Fédération Française de Psychiatrie. Elle répond aux recommandations de la Haute Autorité de Santé et du plan autisme 2013.

Cette recherche ne présente pas de risque pour la santé de votre enfant. Les résultats qui en seront issus ne permettront pas d'apporter des informations pertinentes pour sa santé en particulier. Il bénéficiera de bilans et d'évaluations plus approfondis. Le bénéfice collectif est de favoriser le développement des connaissances et des meilleures stratégies thérapeutiques.

Votre médecin pourra vous informer, sur votre demande, des résultats globaux de cette recherche.

Pour être menée à bien, cette recherche nécessite la mise en œuvre d'un traitement informatisé des données personnelles de votre enfant afin de permettre d'analyser les résultats. Un fichier informatique comportant les données de votre enfant va donc être constitué. Par mesure de confidentialité et pour respecter la vie privée de votre enfant, ses données seront systématiquement codées. Seuls les

professionnels de santé personnellement en charge du suivi de votre enfant auront connaissance de ses données nominatives.

Conformément à la loi, vous disposez d'un droit d'accès, d'opposition et de rectification des données enregistrées sur informatique, à tout moment, par l'intermédiaire du médecin de votre enfant. Vous disposez également d'un droit d'opposition à la transmission des données couvertes par le secret professionnel susceptibles d'être utilisées et d'être traitées dans le cadre de cette recherche. Vous pouvez exercer vos droits d'accès et de rectification auprès du Docteur mentionné au début de ce document.
Cette étude a reçu une autorisation de la Commission Nationale Informatique et Libertés (CNIL).
Ce projet ainsi que le présent document ont été présentés au Groupe Nantais d'éthique dans le domaine de la Santé GNEDS.

Vous êtes libre d'accepter ou de refuser la participation de votre enfant à la recherche qui vous est présentée. Si vous acceptez, vous êtes libre de changer d'avis à tout moment sans avoir à vous justifier et votre décision ne portera aucun préjudice à la qualité de la prise en charge de votre enfant. Si vous refusez la participation de votre enfant, les données ne seront pas utilisées pour cette recherche et resteront destinées à l'usage strict du soin.

Le médecin qui vous a proposé la recherche et vous a donné oralement toutes les informations nécessaires peut répondre à toutes vos questions.

**A compléter par les titulaires de l'autorité parentale**	
Je soussigné(e) :<br>Prénom/Nom : ……………………………………………… ……………………………………………………… ………<br>mère de l'enfant (ou représentant légal), accepte que les données de mon enfant soient utilisées pour cette recherche : □ **oui** □ **non**<br>Date : ………/………/…………<br>Signature :	Je soussigné(e) :<br>Prénom/Nom : ……………………………………………….. ……………………………………………………… …….<br>père de l'enfant (ou représentant légal), accepte que les données de mon enfant soient utilisées pour cette recherche : □ **oui** □ **non**<br>Date : ………/………/…………<br>Signature :

Merci de conserver cette notice d'information

ANNEXE 3 : TABLEAUX/QUESTIONNAIRES UTILISES POUR LES PRATIQUES INTEGRATIVES

TABLEAU 1 – Questionnaire famille (M0)

Enfants atteints d'autisme et difficultés de communication. Questionnaire préalable à la prise en charge

Votre enfant est au début de la prise en charge proposée dans le service …… …..
Nous voudrions recueillir vos opinions sur les questions les plus importantes qui vous inquiètent le concernant.
Pour toute interrogation sur ce questionnaire contactez le service, un professionnel vous repondra.

Quelles sont les initiales de votre enfant et quelle est la date du remplissage.	1ère lettre du prénom : /__/<br>1ère lettre du nom : /__/ Date : /__/__/____/

SECTION 1.1/vos principales inquiétudes concernant votre enfant

Merci de cocher ce qui correspond

Quelles sont vos principales inquiétudes par rapport à votre enfant ?

☐ difficultés d'alimentation	☐ problèmes de sommeil	☐ agressivité	☐ comportement d'opposition	☐ comportements étranges
☐ problèmes relationnels	☐ comportements ritualisés	☐ gestion des émotions	☐ difficultés dans le langage	☐ développement plutôt lent

Merci de commenter et de lister les autres difficultés que vous avez rencontrées :

SECTION 1.2/ autres interventions dont béneficie actuellement votre enfant

Votre enfant reçoit-il une autre intervention en dehors du service en relation à ses besoins (comme ABA, habilités sociales, orthophonie ou autre) ? ☐ oui ☐ non

Si oui, merci de lister et de commenter la façon dont ces interventions ont aidé votre enfant :

SECTION 2.1/ Le développement social de votre enfant

**DEVELOPPEMENT SOCIAL**		Pas du tout<br>0	Parfois<br>1	Souvent<br>2	Très souvent<br>3	Oui Normalement<br>4
**La relation aux autres**	Mon enfant est capable d'établir des relations avec d'autres personnes					
	Mon enfant répond à l'intérêt que d'autres personnes lui montrent					
	Mon enfant a tendance à faire ce qui lui est demandé par d'autres personnes					
	Mon enfant montre de l'intérêt envers les autres					
	Mon enfant semble percevoir la présence d'autres personnes					
**La relation envers moi**	Mon enfant me perçoit en tant que personne					
	Mon enfant comprend l'intérêt d'être ensemble					
	Je n'ai pas besoin de prendre trop de précautions pour rentrer en relation avec mon enfant					
	Mon enfant accepte de faire ce que je lui demande					
	Je me sens détendu en présence de mon enfant					

Merci de commenter le développement social de votre enfant. Qu'est-ce qui vous préoccupe le plus ? Qu'est-ce que vous souhaitez voir se développer ?

SECTION 2.2/ Le développement de la communication de votre enfant.

COMMUNICATION		Pas du tout	Parfois	Souvent	Très souvent	Oui Normalement
		0	1	2	3	4
La communication générale avec les autr	Mon enfant sait communiquer par gestes avec les autres					
	Mon enfant utilise le langage pour communiquer avec les autres					
	Mon enfant comprend ce qui lui est dit					
	Mon enfant est capable de se mettre à la place de quelqu'un d'autre et de comprendre son point de vue					
	Mon enfant communique spontanément ce qu'il ressent.					
	Mon enfant semble s'amuser lorsqu'il joue					
La communication moi	Je peux prévoir les intentions de mon enfant					
	Je me sens contrôlé par mon enfant					
	Mon enfant s'apaise à mes réassurances.					
	Mon enfant est capable de comprendre et exprimer des émotions					

Merci de commenter le développement de la communication de votre enfant. Qu'est-ce qui vous préoccupe le plus ? Qu'est-ce que vous voudriez voir se développer ?

SECTION 2.3/ Le développement imaginaire de votre enfant

	**IMAGINAIRE**	Pas du tout	Parfois	Souvent	Très souvent	Oui Normalement
		0	1	2	3	4
**L' imagination**	Mon enfant montre de la curiosité envers son environnement					
	Mon enfant a une bonne imagination					
	Mon enfant montre une sensibilité adéquate au monde					
	Mon enfant utilise des rituels pour se rassurer					
**Le jeu**	Mon enfant utilise différents jouets lorsqu'il joue					
	Mon enfant est capable de personnifier ses jouets lorsqu'il joue					
	Mon enfant joue beaucoup sur l'ordinateur					
	Mon enfant aime jouer avec ses frères et sœurs ou ses pairs.					

Merci de commenter sur le développement de l'imagination de votre enfant. Qu'est-ce qui vous préoccupe le plus ? Qu'est-ce que vous voudriez voir se développer ?

Merci pour avoir répondu à ce questionnaire, nous vous prions de l'expédier par la poste à l'adresse du service dans lequel votre enfant est pris en charge.

TABLEAU 2 – Questionnaire famille (M12)

Enfants atteints d'autisme et difficultés de communication. Questionnaire d'évaluation à remplir à la fin de l'année de la prise en charge relative au protocole de recherche

Nous vous demandons votre aide afin d'évaluer le service que vous avez reçu au cours de l'année pendant le quel votre enfant a été inclus dans le protocole de recherche. Votre évaluation et vos commentaires sont essentiels pour valider les pratiques intégratives que nous proposons. Ils seront traités dans la plus grande confidentialité.

SECTION 1.1/informations générales

Quelles sont les initiales de votre enfant et quelle est la date du remplissage	1ère lettre du prénom : /__/<br>1ère lettre du nom : /__/ Date:/__/__/____/

Combien de fois votre enfant a été pris en charge lors de son traitement

☐ deux fois par semaine ☐ trois fois par semaine ☐ quatre fois par semaine

Comment qualifieriez-vous le soutien que le service vous a apporté?

☐ Très aidant ☐ Assez aidant ☐ Peu aidant ☐ Inutile

Merci de commenter votre réponse

Les liens qu'il y a eu entre les professionnels du service et les autres intervenants (ex. école, PMI, services sociaux) ont-ils été efficaces ?

☐ oui ☐ non

Merci de commenter votre réponse

SECTION 1.2 /vos principaux questionnements concernant votre enfant

La première fois que vous êtes adressés au service, quelles étaient vos principales inquiétudes par rapport à votre enfant ?<br><br>(Merci de cocher ce qui correspond)	☐ difficultés d'alimentation	☐ problèmes de sommeil	☐ agressivité	☐ colère	☐ comportements étranges
	☐ problèmes relationnels	☐ comportements ritualisés	☐ gestion des émotions	☐ difficultés dans le langage	☐ développement plutôt lent

Merci de commenter et de lister les autres difficultés que vous avez rencontrées :

SECTION 1.3/ autres interventions dont votre enfant a pu bénéficier pendant la période du traitement

Durant son traitement, votre enfant a-t-il reçu une autre forme d'intervention en relation avec ses besoins (ex. ABA, habilités sociales, orthophonie) ?	☐ oui	☐ non

Si oui, merci de lister et de commenter la façon dont ces interventions ont aidé votre enfant :

SECTION 2/ Evaluation générale du service

**Merci de cocher la case qui correspond le mieux à votre expérience**	Au contraire	Pas du tout	En partie	En grande partie	Totalement	Ne correspond pas
	0	1	2	3	4	
Le traitement a eu des retombées bénéfiques sur						

mon enfant						
J'ai pu observer de réels progrès faits par mon enfant durant son traitement						
Le soutien reçu par le service a été bénéfique pour toute la famille						
Les personnes en dehors du cercle familial ont pu eux aussi détecter un changement dans le comportement de mon enfant						
Le soutien que j'ai reçu m'a aidé dans ma réflexion sur le comportement de mon enfant						
Les réunions de bilan tenues lors du traitement ont été bénéfiques						
Je recommanderai ce service à d'autres familles						

Dans l'ensemble, avez-vous été satisfaits du service dont vous avez bénéficié vous et votre famille ? (merci d'entourer)

Pas du tout satisfait très satisfait

1	2	3	4	5	6	7	8	9	10

Merci de commenter votre expérience sur le service dont vous avez bénéficié :

SECTION 3.1/ Le développement social de votre enfant pendant le traitement

**DEVELOPPEMENT SOCIAL**		Pas du tout	Parfois	Souvent	Très souvent	Oui normalement
		0	1	2	3	4
**La relation [illegible] autres**	Mon enfant est plus capable d'établir des relations avec d'autres personnes					
	Mon enfant sait					

	davantage répondre à l'intérêt montré par d'autres personnes					
	Mon enfant a tendance à faire ce qui lui est demandé par d'autres personnes					
	Je sens que mon enfant montre plus d'intérêt envers les autres					
	Mon enfant semble mieux percevoir la présence d'autres personnes					
**La relation envers moi**	Mon enfant semble plus conscient de ma présence en tant que personne					
	Mon enfant comprend mieux l'intérêt d'être ensemble					
	Je n'ai pas besoin de prendre autant de précaution afin de rentrer en relation avec mon enfant					
	Mon enfant accepte de faire ce que je lui demande					
	Je me suis senti beaucoup plus détendu en présence de mon enfant					

Merci de commenter le développement social de votre enfant durant cette période. Y-a t-il eu des améliorations significatives ou de nouvelles difficultés rencontrées ?

SECTION 3.2/ Le développement de la communication de votre enfant pendant la période de traitement

COMMUNICATION		Pas du tout	parfois	souvent	très souven	Oui normalement
		0	1	2	3	4
**La communication générale avec les autres**	Mon enfant sait davantage utiliser les gestes afin de communiquer avec les autres					
	Mon enfant utilise plus le langage afin de communiquer avec les autres					
	Mon enfant comprend mieux ce qui lui est dit					
	Mon enfant semble un peu plus capable de se mettre à la place de l'autre et de comprendre son point de vue					
	Mon enfant se montre plus volontaire afin de faire passer le message qu'il veut transmettre					
	Mon enfant semble s'amuser beaucoup plus lorsqu'il joue					
**La communication avec n**	Je suis plus capable de prévoir les intentions de mon enfant					
	Je me suis senti moins contrôlé par mon enfant					
	Mon enfant semble s'apaiser davantage lorsque je le rassure					
	Mon enfant est plus capable de comprendre et exprimer des émotions					

Merci de commenter le développement de la communication de votre enfant durant cette période. Y-a t-il eu des améliorations significatives ou de nouvelles difficultés rencontrées ?

SECTION 3.3/ Le développement de l'imaginaire de votre enfant pendant le traitement

IMAGINAIRE		Pas du tout	Parfois	Souvent	Très souvent	Oui normalement
		0	1	2	3	4
**L' imagination**	Mon enfant montre plus de curiosités envers son environnement					
	Mon enfant a une meilleure imagination					
	Mon enfant montre une sensibilité plus appropriée envers son environnement					
	Mon enfant utilise moins de rituels pour se rassurer					
**Le jeu**	Mon enfant utilise une plus grande variété de jouets lorsqu'il joue					
	Mon enfant est plus capable de personnifier ses jouets lorsqu'il joue					
	Mon enfant passe moins de temps sur l'ordinateur qu'il ne le faisait					
	Mon enfant aime davantage jouer avec ses frères et sœurs ou avec ses pairs.					

Merci de commenter sur le développement de l'imagination de votre enfant et la place prise par l'imaginaire dans la vie quotidienne durant cette période. Y a-t-il eu des améliorations ou d'autres problèmes sont-ils apparus ?

SECTION 4.1/ Appréciation de l'état de l'enfant

**Comment qualifiez-vous l'évolution de l'enfant au terme de cette année ?**		Aggravation<br>0	Aucune évolution<br>1	Peu d'évolution<br>2	Evolution satisfaisante<br>3	Evolution satisfaisante<br>4
**DOMAINES D' INQUIETUDE**	Difficultés d'alimentation	☐	☐	☐	☐	☐
	Problèmes de sommeil	☐	☐	☐	☐	☐
	Agressivité	☐	☐	☐	☐	☐
	Colère	☐	☐	☐	☐	☐
	Comportements étranges	☐	☐	☐	☐	☐
	Problèmes relationnels	☐	☐	☐	☐	☐
	Comportements ritualisés	☐	☐	☐	☐	☐
	Gestion des émotions	☐	☐	☐	☐	☐
	Difficultés dans le langage	☐	☐	☐	☐	☐
	Développement plutôt lent	☐	☐	☐	☐	☐

Merci. Le temps et les efforts que vous avez fournis pour compléter ce questionnaire sont sincèrement appréciés. Vous pouvez faire appel aux professionnels du service si vous avez besoin d'explications le concernant.
Vos réponses et commentaires sont des éléments essentiels de la recherche en cours pour évaluer et améliorer la prise en charge proposée aux enfants atteints d'autisme.
Nous vous remercions de nous faire parvenir le questionnaire rempli par la poste ou de le transmettre à un des professionnels référent de votre enfant.

Tableau 3 – Questionnaire professionnels (M0)

Enfants atteints d'autisme et difficultés de communication. Questionnaire préalable à la prise en charge

Cet enfant est au début de la prise en charge proposée dans le service …… …..
Nous voudrions savoir quels sont les symptômes qui vous semblent au premier plan.

Quel est le code d'enregistrement de cet enfant et quelle est la date du remplissage	Code patient : Date : /__/__/____/

SECTION 1.1/ quels sont les principaux symptômes :

Merci de cocher ce qui correspond

Quels sont les principaux symptômes que vous observez chez cet enfant ?					
	☐ difficultés d'alimentation	☐ problèmes de sommeil	☐ agressivité	☐ comportement d'opposition	☐ comportements étranges
	☐ problèmes relationnels	☐ comportements ritualisés	☐ gestion des émotions	☐ difficultés dans le langage	☐ développement plutôt lent

Merci de commenter et de lister les autres symptômes que vous avez remarqués :

SECTION 1.2/ autres interventions dont bénéficie actuellement cet enfant

L'enfant reçoit-il une autre intervention en dehors du service en relation à ses besoins (comme ABA, habilités sociales, orthophonie ou autre) ?	☐ oui	☐ non

Si oui, merci de lister et de commenter la façon dont ces interventions ont aidé cet enfant :

SECTION 2.1/ Le développement social de cet enfant

DEVELOPPEMENT SOCIAL		Pas du tout	Parfois	Souvent	Très souvent	Oui Normalement
		0	1	2	3	4
**La relation aux autres**	L'enfant est capable d'établir des relations avec d'autres personnes					
	L'enfant répond à l'intérêt que d'autres personnes lui montrent					
	L'enfant a tendance à faire ce qui lui est demandé par d'autres personnes					
	L'enfant montre de l'intérêt envers les autres					
	L'enfant semble percevoir la présence d'autres personnes					
**La relation envers moi**	L'enfant me perçoit en tant que personne					
	L'enfant comprend l'intérêt d'être ensemble					
	Je n'ai pas besoin de prendre trop de précautions pour rentrer en relation avec l'enfant					
	L'enfant accepte de faire ce que je lui demande					
	Je me sens détendu en présence de cet enfant					

Merci de commenter le développement social de cet enfant. Qu'est-ce qui vous préoccupe le plus ? Qu'est-ce que vous souhaitez voir se développer ?

SECTION 2.2/ Le développement de la communication de cet enfant.

COMMUNICATION		Pas du tout	Parfois	Souvent	Très souvent	Oui Normalement
		0	1	2	3	4
La communication générale avec les au	L'enfant sait communiquer par gestes avec les autres					
	L'enfant utilise le langage pour communiquer avec les autres					
	L'enfant comprend ce qui lui est dit					
	L'enfant est capable de se mettre à la place de quelqu'un d'autre et de comprendre son point de vue					
	L'enfant communique spontanément ce qu'il ressent.					
	L'enfant semble s'amuser lorsqu'il joue					
La communication moi	Je peux prévoir les intentions de l'enfant					
	Je me sens contrôlé par l'enfant					
	L'enfant s'apaise à mes réassurances.					
	L'enfant est capable de comprendre et exprimer des émotions					

Merci de commenter le développement de la communication de cet enfant. Qu'est-ce qui vous préoccupe le plus ? Qu'est-ce que vous voudriez voir se développer ?

SECTION 2.3/ Le développement imaginaire de cet enfant

	IMAGINAIRE	Pas du tout	Parfois	Souvent	Très souvent	Oui Normalement
		0	1	2	3	4
**L' imagination**	L'enfant montre de la curiosité envers son environnement					
	L'enfant a une bonne imagination					
	L'enfant montre une sensibilité adéquate au monde					
	L'enfant utilise des rituels pour se rassurer					
**Le jeu**	L'enfant utilise différents jouets lorsqu'il joue					
	L'enfant est capable de personnifier ses jouets lorsqu'il joue					
	L'enfant joue beaucoup sur l'ordinateur					
	L'enfant aime jouer avec ses frères et sœurs ou ses pairs.					

Merci de commenter sur le développement de l'imagination de cet enfant. Qu'est-ce qui vous préoccupe le plus ? Qu'est-ce que vous voudriez voir se développer ?

Tableau 4 – Questionnaire professionnels (M12)

Enfants atteints d’autisme et difficultés de communication. Questionnaire d’évaluation à remplir à la fin de l’année de la prise en charge relative au protocole de recherche

Nous vous demandons de remplir le même questionnaire qui est soumis aux parents afin d’évaluer le service que reçu au cours de l’année pendant lequel cet enfant a été inclus dans le protocole de recherche.
Votre évaluation et vos commentaires sont essentiels pour valider les pratiques intégratives que nous proposons. Ils seront traités dans la plus grande confidentialité.

SECTION 1.1/informations générales

Quel est le code d’enregistrement de cet enfant et quelle est la date du remplissage	Code patient : Date : /__/__/____/

Combien de fois l’enfant a été pris en charge lors de son traitement

☐ deux fois par semaine ☐ trois fois par semaine ☐ quatre fois par semaine

Comment qualifieriez-vous le soutien que le service a apporté?

☐ Très aidant ☐ Assez aidant ☐ Peu aidant ☐ Inutile

Merci de commenter votre réponse

Les liens qu’il y a eu entre les professionnels du service et les autres intervenants (ex. école, PMI, services sociaux) ont-ils été efficaces ?

☐ oui ☐ non

Merci de commenter votre réponse

SECTION 1.2 /<u>Les principaux symptômes que vous avez observé concernant cet enfant</u>

La première fois que l'enfant est arrivé dans le service, quels étaient les principaux symptômes que vous avez observés chez cet enfant ? (Merci de cocher ce qui correspond)	☐ difficultés d'alimentation	☐ problèmes de sommeil	☐ agressivité	☐ colère	☐ comportements étranges
	☐ problèmes relationnels	☐ comportements ritualisés	☐ gestion des émotions	☐ difficultés dans le langage	☐ développement plutôt lent

Merci de commenter et de lister les autres symptômes que vous avez remarqués:

SECTION 1.3/ <u>autres interventions dont cet enfant a pu bénéficier pendant la période du traitement</u>

Durant son traitement, cet enfant a-t-il reçu une autre forme d'intervention en relation avec ses besoins (ex. ABA, habilités sociales, orthophonie) ?

☐ oui ☐ non

Si oui, merci de lister et de commenter la façon dont ces interventions ont aidé cet enfant :

SECTION 2/ <u>Evaluation générale du service</u>

**Merci de cocher la case qui correspond le mieux à votre expérience**	Au contraire	Pas du tout	En partie	En grande partie	Totalement	Ne correspond pas
	0	1	2	3	4	
Le traitement a eu des retombées bénéfiques sur l'enfant						
J'ai pu observer de réels progrès faits par l'enfant durant son traitement						
Le soutien reçu par le service a été bénéfique pour toute la famille						
Les personnes en dehors des professionnels concernés ont pu eux aussi détecter un changement dans le comportement de l'enfant						
Le soutien donné aux parents les ont aidés dans leur réflexion sur le comportement de leur enfant						
Les réunions de bilan tenues lors du traitement ont été bénéfiques						
Ils recommanderaient ce service à d'autres familles						

Dans l'ensemble, vous pensez que les parents ont été satisfaits du service dont ils ont bénéficié eux et leur famille ? (merci d'entourer)

Pas du tout satisfait — très satisfait

1	2	3	4	5	6	7	8	9	10

Merci de commenter l'expérience que les parents ont partagé avec le service durant cette période :

SECTION 3.1/ Le développement social de cet enfant pendant le traitement

**DEVELOPPEMENT SOCIAL**		Pas du tout<br>0	Parfois<br>1	Souvent<br>2	Très souvent<br>3	Oui normalement<br>4
**La relation aux autres**	L'enfant est plus capable d'établir des relations avec d'autres personnes					
	L'enfant sait davantage répondre à l'intérêt montré par d'autres personnes					
	L'enfant a tendance à faire ce qui lui est demandé par d'autres personnes					
	Je sens que l'enfant montre plus d'intérêt envers les autres					
	L'enfant semble mieux percevoir la présence d'autres personnes					
**La relation envers moi**	L'enfant semble plus conscient de ma présence en tant que personne					
	L'enfant comprend mieux l'intérêt d'être ensemble					
	Je n'ai pas besoin de prendre autant de précaution afin de rentrer en relation avec l'enfant					
	L'enfant accepte de faire ce que je lui demande					
	Je me suis senti beaucoup plus détendu en présence de cet enfant					

Merci de commenter le développement social de cet enfant durant cette période. Y-a t-il eu des améliorations significatives ou de nouvelles difficultés rencontrées ?

SECTION 3.2/ Le développement de la communication de cet enfant pendant la période de traitement

COMMUNICATION		Pas du tout	parfois	souvent	très souven	Oui normalement
		0	1	2	3	4
**La communication générale avec les autres**	L'enfant sait davantage utiliser les gestes afin de communiquer avec les autres					
	L'enfant utilise plus le langage afin de communiquer avec les autres					
	L'enfant comprend mieux ce qui lui est dit					
	L'enfant semble un peu plus capable de se mettre à la place de l'autre et de comprendre son point de vue					
	L'enfant se montre plus volontaire afin de faire passer le message qu'il veut transmettre					
	L'enfant semble s'amuser beaucoup plus lorsqu'il joue					
**La communication avec m**	Je suis plus capable de prévoir les intentions de cet enfant					
	Je me suis senti moins contrôlé par cet enfant					
	L'enfant semble s'apaiser davantage lorsque je le rassure					
	L'enfant est plus capable de comprendre et exprimer des émotions					

Merci de commenter le développement de la communication de cet enfant durant cette période. Y-a t-il eu des améliorations significatives ou de nouvelles difficultés rencontrées ?

SECTION 3.3/ Le développement de l'imaginaire de cet enfant pendant le traitement

**IMAGINAIRE**		Pas du tout	Parfois	Souvent	Très souvent	Oui normalement
		0	1	2	3	4
**L' imagination**	L'enfant montre plus de curiosités envers son environnement					
	L'enfant a une meilleure imagination					
	L'enfant montre une sensibilité plus appropriée envers son environnement					
	L'enfant utilise moins de rituels pour se rassurer					
**Le jeu**	L'enfant utilise une plus grande variété de jouets lorsqu'il joue					
	L'enfant est plus capable de personnifier ses jouets lorsqu'il joue					
	L'enfant passe moins de temps sur l'ordinateur qu'il ne le faisait					
	L'enfant aime davantage jouer avec ses frères et sœurs ou avec ses pairs.					

Merci de commenter sur le développement de l'imagination de cet enfant et la place prise par l'imaginaire dans la vie quotidienne durant cette période. Y a-t-il eu des améliorations ou d'autres problèmes sont-ils apparus ?

SECTION 4.1/ Appréciation de l'état de l'enfant

**Comment qualifiez vous l'évolution de l'enfant au terme de cette année ?**		Aggravation	Aucune évolution	Peu d'évolution	Evolution satisfaisante	Evolution satisfaisante
		0	1	2	3	4
**DOMAINES D' INQUIETUDE**	Difficultés d'alimentation	☐	☐	☐	☐	☐
	Problèmes de sommeil	☐	☐	☐	☐	☐
	Agressivité	☐	☐	☐	☐	☐
	Colère	☐	☐	☐	☐	☐
	Comportements étranges	☐	☐	☐	☐	☐
	Problèmes relationnels	☐	☐	☐	☐	☐
	Comportements ritualisés	☐	☐	☐	☐	☐
	Gestion des émotions	☐	☐	☐	☐	☐
	Difficultés dans le langage	☐	☐	☐	☐	☐
	Développement plutôt lent	☐	☐	☐	☐	☐

Merci. Vos réponses et commentaires sont des éléments essentiels de la recherche en cours pour évaluer et améliorer la prise en charge proposée aux enfants atteints d'autisme.

**TABLEAU 5 – Inventaire des facteurs externes**				
Noter les évènements importants				Feuille N°
INVENTAIRES DES FACTEURS EXTERNES				
Enfant	Code :			
Evènements signifiants				
			Date	Nature
Concernant l'enfant				
	Somatiques			
		Hospitalisation		
		Maladie aîgue		
		Maladie chronique		
		Accidents		
	Accueil			
		Changement de PEC		
		Scolarisation		
Concernant la famille	Somatiques			
		Hospitalisation		
		Maladie		
		Décès		
		Accidents		
	Naissance			
	Séparation			
	Déménagement			
	Socio-économique			

**TABLEAU 6 – Harmonisation des pratiques**			
Cocher la case correspondant à votre choix			
Amélioration des pratiques			
Connaissance des échelles dans le but d'homogénéiser les pratiques			
NOM : :<br><br>PROFESSION :	CENTRE :	OUI	NON
ECAR - T			
L'ECAR-T est une échelle de comportement autistique		☐	☐
L'ECAR - T est une échelle d'évaluation de l'intensité de l'autisme infantile		☐	☐
L'ECAR-T donne le profil psycho-éducatif		☐	☐
Elle explore le fonctionnement de l'enfant par symptômes		☐	☐
Les symptômes suivant font partie des 29 items de la grille :			
	Regard inadéquat	☐	☐
	Difficulté à communiquer par le geste et la mimique	☐	☐
	Utilise les objets de manière irrésisitible et/ou ritualisée	☐	☐
	Bizarreiries de l'audition	☐	☐
	Utilisation du corps	☐	☐
Les items sont cotés de 0 à 4		☐	☐
Le score augmente avec le niveau d'autisme		☐	☐
CARS			
La CARS donne le profil psycho-éducatif		☐	☐
La CARS est une échelle d'évaluation de l'intensité de l'autisme infantile		☐	☐
La CARS est une échelle de comportement autistique		☐	☐

Les catégories suivantes font partie de la CARS :			
	Utilisation du corps	☐	☐
	Troubles des conduites alimentaires	☐	☐
	Adaptation au changement	☐	☐
	Réponses visuelles	☐	☐
	Peur Anxiété		
Les items sont cotés de 1 à 4			
Entre 30 et 40 :	l'enfant est non autistique	☐	☐
	l'enfant est légèrement ou moyennement autistique	☐	☐
	l'enfant est sévèrement autistique	☐	☐
PEP - 3			
Le PEP - 3 est une échelle de comportement autistique		☐	☐
Le PEP - 3 donne le profil psycho-éducatif		☐	☐
Le PEP - 3 est une échelle d'évaluation de l'intensité de l'autisme infantile		☐	☐
Il comporte 113 items de développement et 43 de comportement		☐	☐
Les tests de performance suivants font partie du PEP-3 :			
	Cognition verbale/préverbale	☐	☐
	Imitation oculo-motrice	☐	☐
	Agitation/turbulence	☐	☐
	Problèmes de comportement	☐	☐
	Autonomie personnelle	☐	☐
Les tests sont cotés de 0 à 2		☐	☐
Le score est global		☐	☐
Le score est par tests		☐	☐
Le score le plus élevé est le plus grave		☐	☐
Appréciation qualitative fin de recherche			

Votre pratique a été améliorée depuis le début de la recherche pour :			
	l'observation clinique	☐	☐
	l'évaluation de l'état de l'enfant	☐	☐
	l'ajustement des propositions thérapeutiques	☐	☐
	la collaboration avec la famille	☐	☐
	les connaissances théoriques	☐	☐

**TABLEAU 7 – Observation clinique strcucturée**		
TRAME DE L'OBSERVATION CLINIQUE STRUCTUREE		
Anamnèse		
	Personnelle	
		Somatique
		Développementale
		Environnementale
		Scolarisation
	Familiale	
Interactions sociales		
	Regard	
	Mimiques faciales	
	Attitude corporelle	
	Partage des émotions, intérêts, activités	
	Intégration des comportements, codes sociaux	
Communication		
	Pointage impératif pointage protodéclaratif<br>attention conjointe<br>retard ou absence de langage	
	Stéréotypies verbales	
	Jeu de faire-semblant, jeu d'imitation sociale, jeu symbolique	
Comportements		
	Intérêts restreints et répétés, anormaux par leur intensité ou caractère limité,<br>retrait, repli	
	Rituels spécifiques non fonctionnels	
	Stéréotypies gestuelles	
	Préoccupations limitées à certaines parties des objets (reniflement, odeur, sensation surface, bruits, vibrations)	

TABLEAU 8 – Projet de soin individualisé					
**PROJET DE SOIN INDIVIDUALISE**					
Feuille N° :	CODE				Révision N°
					Révisé le :
	DATE : /__/__/____/		Age d'entrée dans étude		
Médecin / Psychologue coordonateur Nom					
Référents infirmier/éducateur Noms					
**Ateliers et PEC (réf tableaux des activités thérapeutiques en annexe)**					
**Noms des ateliers**					
Numéro d'identification du groupe de l'intervention					
Individuel					
Groupe					
Fréquence					

Nombre d'enfants dans le groupe					
Durée de la séance					
**Psychomotricité**					
Fréquence					
Individuel					
Groupe					
Nombre d'enfants dans le groupe					
Durée de la séance					
**Orthophonie**					
Fréquence					
Individuel					
Groupe					
Nombre d'enfants dans le groupe					
Durée de la séance					
**Visites à domicile ou sur le lieu d'accueil**					
Fréquence					
**Entretiens parent-enfant**					
Psychologue ou pédopsy					
Coordonnateur/Infirmier ou éduc					
Fréquence					

**Psychothérapie enfant seul**					
Fréquence					
**Entretiens familles**					
Médecin coordonateur/psychologue					
Infirmier / éducateur					
Dates					
	PEC extérieures				
**Accueil en multi-accueil**					
Demi-journée					
Journée					
Fréquence					
**Scolarisation**					
Demi-journée					
Journée					
Fréquence					
**Interventions ext**					
Orthophoniste					
Ergothérapeute					
Autres…					

	Articulations intra et extra-institutionnelles				
**Articulations internes**					
Synthèse Fréquence					
Supervision atelier, nombre, fréquence					
**Articulations Externes**					
Dossier MDPH					
Articulation avec l'école					
Articulation avec une institution médico-sociale					
Articulation autre (professionnel ou institution)					
	Somatique - Prise en charge pharmacologique				
**Douleur exploration clinique**					
Examens somatiques					
Traitements médicamenteux psychotropes	Noms :		Prescriptions, durée :		

TABLEAU 9	
**TABLEAU DES INTERVENTIONS THERAPEUTIQUES ET EDUCATIVES RELATIVES AUX OBSERVATIONS CLINIQUES, AUX HYPOTHESES PSYCHOPATHOLOGIQUES ET FONCTIONNELLES QUI LEUR SONT ASSOCIEES**	
Domaines-Dimensions /Eléments sémiologiques	Interventions thérapeutiques et/ou éducatives
Sensori-moteur	
**Dans le domaine sensoriel** : anomalies sensorielles auditives, visuelles, tactiles, olfactives et gustatives	
Observations : Evitements, recherche répétitive de la stimulation, absence apparente ou réelle à l'environnement, captation dans une seule modalité perceptive, difficultés à intégrer deux modalités sensorielles	Eveil sensoriel, atelier chant, mélodie, percussion, sons, goût, odeur A1<br>Cuisine A2<br>Jeux d'eau, pataugeoire A3
**Dans le domaine moteur** :	
Troubles moteurs, du tonus, de la posture, de l'intégration de l'image du corps et de l'enveloppe corporelle.	Jeux moteurs, psychomotricité A4<br>Atelier poney, piscine A5
Difficultés à interagir avec l'environnement, troubles de l'apprentissage de la propreté	
Dans le domaine sensori-moteur, la différenciation de soi et de l'autre est altérée.	
Communication	Supports visuels permettant anticipation et prévisibilité (pictogramme…)
**Non verbale** : regard, pointage, attention conjointe, imitation, mimique, posture, geste symbolique, faire semblant	Orthophonie, moyens augmentatifs type PECS, Makaton B2
Reconnaissance des émotions, des situations sociales. Difficulté du partage des émotions, de l'inter modalité, de l'accordage affectif.	Ateliers mimes, comptines, marionnettes, jeux de personnages B1
**Verbale :** compréhension et expression	Orthophonie B2
Les altérations du langage verbal versant expressif (inversion pronominale, écholalie… et évocation) et versant réceptif : compréhension verbale et compréhension sociale	
Défaut d'attention conjointe, d'échange, de partage des émotions, de l'intermodalité et de la compréhension de l'implicite, de la continuité et des représentations	Contes (plutôt saynettes ou histoires simples), mise en récit B3

Les comportements secondaires : l'utilisation particulière du langage dans sa forme (stéréotypie, maniérisme…), dans son usage (dimension non pragmatique) et la distorsion de la compréhension	
Emotion, angoisse, comportement	Créer un contenant sécure : repères fixes et différentes formes d'enveloppement, (physique, sonore), la structuration du temps et de l'espace
Les angoisses identitaires (sensorielles, motrices), peurs massives, terreurs primitives	Atelier expression, discrimination des émotions C1
Défaut d'un contenant sécure qui s'exprime dans les stéréotypies, auto ou hétéro agressivité	
Défaut d'organisation des émotions et des sensations, conduites de décharges	
Défaut de compréhension des situations dans lesquelles il est plongé et difficulté d'expression adaptée. Incapacité à sortir de l'immuabilité par angoisse de changement	Activités de développement de la communication, de la diversification des investissements : Habilités sociales C2
Les comportements secondaires : stéréotypies, auto ou hétéro agressivité, agitation, crises de colère ; inhibition, repli, passivité ; troubles fonctionnels (sommeil, alimentation, transit)	Activités de mise en situation aménagée C2
Les angoisses secondaires (altération de la communication et de la compréhension de l'autre)	
Crises de tantrum. Désorganisation physique et psychique	Aménagement d'espaces, de temps d'apaisement C3
Troubles du comportement révélateurs d'une douleur. Difficulté pour identifier ou exprimer de façon adéquate la douleur	Recherche de l'origine et prescriptions médicamenteuses nécessaires C4
Socialisation	Le premier temps peut être en situation duelle
Contact visuel, ajustement tonico-moteur, manque de pointage et d'attention conjointe, défaillance de l'imitation sociale et de l'exploration de l'environnement, recherche de l'immuabilité et difficulté d'établir une relation interpersonnelle	Amélioration de la qualité des échanges, compréhension des codes sociaux, plaisir à la relation à travers un certain nombre de situations « séquencées », reliées par la mise en récit, généralisation des situations D1
Retrait autistique, isolement, résistance aux changements, altération des interactions sociales	Atelier jeux de rôle, habiletés sociales, jeux éducatifs, temps extérieurs (cour, sortie…) mise en situation, temps du repas D2
	Echanges avec les parents, le milieu scolaire D3
Educatif	Simplifier les tâches et les consignes verbales, structurer l'espace et le temps

Autonomie : alimentation, hygiène corporelle, habillage…<br>Tendance à l'immuabilité frein à l'autonomie	
Particularités sensorielles et difficultés d'intégration de l'image de soi compromettent les acquisitions nécessaires à l'autonomie	Activités quotidiennes, repas, mettre le couvert, jeu de dinette, cuisine, repas thérapeutique, acquisition de la propreté E1
Difficulté dans les praxies (coordination œil/main, identification des objets, améliorer la tenue de l'objet, affinement de la perception visuelle..)	Développer les compétences psychomotrices globales, les praxies E2
Relations avec la famille	Consultations
	Echanges informels
	Discussion du projet
	Prise en compte des évènements importants intercurrents
	VAD
	Groupe de parole parents ou fratrie
	Entretien avec assistant social
Cognitif	Structuration du temps, structuration de l'espace, utilisation de pictogrammes ou autres supports concrets
Capacité de penser, de faire des liens, difficulté à catégoriser, à comprendre l'implicite	Jeux d'imitation, encastrements, puzzles, tri d'objets, travail sur la catégorisation... F1
	Logiciel informatique F2
Défaut de théorie de l'esprit. Difficulté à généraliser, traitement séquentiel de l'information, focalisation sur les détails	Classe intégrée dans l'unité des soins et plus souvent, à cette tranche d'âge, inclusion en classe maternelle
Somatique et pharmacologique	Contribuer à améliorer l'accès aux soins des enfants autistes
Attention au suivi somatique habituel de l'enfant.<br>Habituer l'enfant aux soins (hygiène, visite chez le médecin, le dentiste)	Faire les prescriptions médicamenteuses nécessaires<br>Habituer l'enfant aux visites médicales de routine
Changement de comportement<br>Dépister les phénomènes d'hypersensibilité, dépister la douleur	Refaire à intervalles réguliers les bilans neuropédiatrique, ORL, OPH et orthoptique<br>Connaître les manifestations de la douleur chez l'enfant
Articulations intra et extra-institutionnelles	
**Articulations internes**	

Maintenir la cohérence d'un travail d'équipe à travers des temps de réflexion et d'élaboration.<br>Le projet de soin individualisé peut comporter des prises en charge individuelle ou groupale, en psychomotricité, en orthophonie, en psychothérapie ou sous forme d'intervention éducative.	Synthèse, reprise, supervision, formation
Les temps de réflexion et d'élaboration rythment l'ensemble de la prise en charge et assurent sa cohérence	
**Articulations externes**	
Articulations avec les autres institutions : école, médico-social	Réunions, échanges téléphoniques etc.,
Articulation entre unité de prise en charge et unité d'évaluation.<br>Définir des objectifs communs	Attention particulière pour les liens réciproques
Articulation avec la MDPH	Informer et accompagner les parents

ANNEXE 4 : DÉTAIL DE LA GRILLE BUDGÉTAIRE ET DEVIS

La durée totale du projet est de 40 mois (24 mois d'inclusion, 12 mois de suivi et 4 mois d'analyse et de publication).
80 enfants seront inclus dans ce projet.
Nombre de centres associés : 17 centres participants

- **TITRE I – Dépenses de personnels affectés à la réalisation de la recherche**

  - **Missions d'investigation**

<u>Médecin coordonnateur- PU-PH</u> (Nicole Garret-Gloanec) **<u>et co-coordonnateurs – PU-PH</u>** (Maria Squillante et Fabienne Roos Weil) :

- Coordination et suivi du projet (interlocuteurs principaux pour les centres, réunions de mise en place…) : 4 jours par mois soit 160 jours

- Echanges sur place auprès des équipes + temps de déplacement : 25 jours

- discussion et diffusion des résultats : 10 jours

→ 195 jours soit 11 mois.personne sur 40 mois → **55 000 euros**

<u>Médecins investigateurs</u>- PH:

- Inclusions des patients : 20 min/patient soit 4 jours pour 80 patients

- Remplissage des CRF papier et envoi des CRF : 5h/patient soit 53 jours

- Réunion de mise en place, liens avec les investigateurs coordonnateurs et réunions de formation : 5 jours soit 85 jours (17 investigateurs)

→ 142 jours au total sur les 40 mois de l'étude soit 8 mois.personne pour les 17 investigateurs **80 000 euros** →

<u>TEC coordinateur Nantes</u> :

- Relance des centres : 3 jours/mois sur 3 ans soit 108 jours

- Envoi et réception des CRF papier : 2 jours/mois sur 3 ans soit 72 jours

- Saisie des données, archivage des documents : 2 jours/mois sur 3 ans soit 72 jours

→ 252 jours sur 3 ans soit 15 mois.personne → **63 938 euros**

<u>Psychologue de l'étude</u>:

- Réunions dans centres pour présenter les outils des pratiques intégratives : 1 réunion d'une journée par centre soit 20 jours (en comptant le temps de transport)

- Analyse des vidéos à M0 (1 vidéo/enfant) et M12 (1 vidéo/enfant) : ½ journée/vidéo soit 90 jours (en comptant le temps de transport)

→110 jours sur 3 ans soit 7 mois.personne → **34 588 euros**

Il n'y a pas de temps TEC prévu dans les autres centres associés car ce sont les pédopsychiatres qui se chargeront, en même de temps que leur consultation, de remplir le CRF papier et de le retourner au CHU de Nantes.

Psychologues « indépendants » (au nombre de 2) :

- Analyse des vidéos à M0 et M12, soit 160 vidéos à analyser (2 vidéos par enfant : M0 et M12) en comptant ½ journée/vidéo soit 80 jours

→ 80 jours sur 3 ans soit 5 mois.personne → **24 706 euros**

- **Missions d'organisation et/ou de coordination de la recherche**

Chef de projet :

- mise en place réglementaire et administrative du projet (CCTIRS/CNIL/GNEDS/Conventions) : 12 jours
- Suivi du projet : 4 jours/an soit 12 jours
- Archivage : 2 jours

→ 26 jours au total soit 2 mois.personne→ **2 097euros**

- **Missions de gestion et d'analyse des données**

Méthodologiste (Jean-Benoit Hardouin) : 1 mois d'aide à la méthodologie → **3 234 euros**

Data-Manager : 44 jours pour le développement de la base Clinsight, la gestion des queries, la maintenance, l'extraction des données, le gel de base soit 3 mois.personne → **9 702 euros** (sur devis)

Biostatisticien (Jean-Benoit Hardouin) : 2 mois d'analyse soit 2 mois.personne → **6 468 euros**

Soit un total éligible pour le Titre I de 293 233 euros

- **TITRE III – Dépenses à caractère hôtelier et général**

Surcoûts liés aux frais d'affranchissement : Forfait de **200 euros**

Surcoûts liés aux frais de missions :

- Réunions de formation des centres : 1 réunion de formation en début d'étude par le psychologue ou le médecin coordonnateur, 1 réunion en milieu d'étude si besoin, 1 déplacement sur site pour visualiser les vidéos soit 51 déplacements à 450 euros par déplacement national (forfait selon grille GT5) → Soit un total de **22 950 euros**

- Frais de missions/congrès : Participation à 3 congrès (A titre d'exemple : Congrès Français de Psychiatrie, Congrès de la World Psychiatric Association, congrès nationaux de la SFPEADA-API, SIP, à l'université d'automne de l'Arapi au Croisic et l'ANCRA à Tours) pour les 3 coordonnateurs de l'étude avec un forfait de 500 euros par congrès (frais d'inscription et de déplacement) : soit un total de **4 500 euros**

Surcoûts liés aux frais d'impression, de publication :

- Frais de publication/Traduction : Forfait de **2 000 euros**
- Frais de reprographie : **523 euros** (sur devis du service de reprographie ANR)

Surcoûts liés aux frais d'archivage : Forfait de **500 euros**

Soit un total éligible pour le Titre III de 30 673 euros

TOTAL ELIGIBLE A LA SUBVENTION DGOS : 323 906 euros

DEVIS BASE CLINSIGHT

Cellule de Promotion de la Recherche Clinique - Data Management
5, Allée de l'Ile Gloriette - 44093 NANTES Cedex 01

CENTRE HOSPITALIER UNIVERSITAIRE DE NANTES

CREATION DE LA BASE DE DONNEES, SAISIE & DATA MANAGEMENT

Projet AUTISME - Dr. Garret-Gloanec - PREPS 2013

Paramètres

Projet	AUTISME
Type	PREPS
Début	01/01/2014
Fin	01/01/2016
Durée (Années)	2
Nb de patients	80
Nb de pages imprimées	55
Nb écrans	40
Nb Items	800

Ventilation des coûts

	Quantité	Unité	Durée (jours)	Taux horaire CPRC	Qui	Début de projet	Année n	Fin de projet	Total €
**Création eCRF et base de données :**									
Conception et création de la maquette du CRF	0	0,00	0,00	39,00 €	DM	-	-	-	**0,00 €**
Conception et création de la base de données	1	0,50	0,50	39,00 €	DM	137	-	-	**137,00 €**
Administration et gestion des comptes utilisateurs	1	0,25	0,25	39,00 €	DM	68	-	-	**68,00 €**
Création des masques de saisie	40	0,50	20,00	31,00 €	DM (*)	4340	-	-	**4 340,00 €**
Validation de l'application	0	0,25	0,00	29,00 €	ARC	0	-	-	**0,00 €**
**Plan de validation des données :**									
Rédaction du plan de validation des données	1	0,50	0,50	39,00 €	DM	137	-	-	**137,00 €**
Programmation des tests de cohérence et contrôles en ligne	40	0,25	10,00	31,00 €	DM (*)	2170	-	-	**2 170,00 €**
Approbation du plan de validation	0	0,25	0,00	29,00 €	ARC	0	-	-	**0,00 €**
**Stockage & hébergement des images DICOM**									
Paramétrage du serveur & du site Web	0	10,00	0,00	39,00 €	DM	0	-	-	**-**
Mise en place du process (validation et contrôle qualité)	0	1,00	0,00	39,00 €	DM	0	-	-	**-**
**Gel de la base et transfert des données :**									
Programmation des tables d'export	40	0,10	4,00	39,00 €	DM	1092	-	-	**1 092,00 €**
Gestion et transfert des données (1/an)	1	0,25	0,25	31,00 €	DM (*)	-	54	54	**108,00 €**
Gel de la base de données	1	1,00	1,00	31,00 €	DM (*)	-	-	217	**217,00 €**
**Rapport de Data Management :**									
Rédaction du rapport (spécifications techniques, CRF annoté...)	0	5,00	0,00	39,00 €	DM	-	-	-	**0,00 €**
**Saisie :**									
Rédaction de la convention de saisie	1	0,50	0,50	31,00 €	DM (*)	109	-	-	**109,00 €**
Simple saisie des données (par une CRO)	0	350,00	0,00	38,00 €	CRO	-	-	-	**0,00 €**
Contrôle qualité de la saisie (5% des données)	0	1500,00	0,00	31,00 €	DM (*)	-	-	-	**0,00 €**
**Gestion des Queries :**									
Gestion des Queries (1% de données)	640	1000,00	0,60	31,00 €	DM (*)	-	130	130	**260,00 €**
Pré-traitement des queries (par le Data Manager)	2200	350,00	6,30	31,00 €	DM (*)	-	1367	-	**1 367,00 €**
Contrôle qualité du traitement des Queries (10% des Queries)	0	350,00	0,00	31,00 €	DM (*)	-	0	-	**-**
**Total € TTC Data Management**			44						**10 005,00 €**
**Total € TTC Clinique**			0						**0,00 €**
**Total € TTC toutes prestations**			44						**10 005,00 €**

Estimation du 20/09/2013 Tanguy ROMAN ☎ 02.53.48.28.37 *tanguy.roman@chu-nantes.fr*

Grille Data Management version 5.0 du 27/09/2011

DM(*) : Coût différencié entre Data Manager Junior et Senior

DEVIS REPROGRAPHIE (ANR)

ANR SERVICES

Devis

Page : 1 de 4
Numéro : DV00000440-3
Date : 19/09/2013

ANRH EA Nantes
Nant'Est Entreprises
32, rue du Marché Commun
BP 33245

44332 NANTES CEDEX 3
FRA

NAF : 8810C
SIRET : 77566097000283

Téléphone : 02 40 50 00 99
Télécopie : 02 40 50 13 54

CHU DE NANTES
POLE TECHNIQUE ET LOGISTIQUE
Service Prestations Achats Hotelier
85 RUE SAINT JACQUES
44093 NANTES
FRA

Votre N° de TVA :
Votre compte client : C070000056
Commande client :
Bon de commande :
Votre réf. : PROJET AUTISME
Paiement : 30 jours date de facture

Devis
DEVIS ETABLI PAR CHRISTELLE KERREVEUR
TEL 02 40 52 19 39

Numéro d'article	Description	Date d'expédition	Quantité	Unité	Prix de vente	Remise %	Remise	Montant Vente
A070001109	DESIGNATION CHU<br>MARCHE N° 10 - 0119<br>REFERENCE ETUDE : NA<br>CODE UF : NA<br>*********************<br>COMMANDE DE MME<br>LEA FERRAND<br>TEL02 53 48 28 10<br>**********************<br>DATE DEMANDE :<br>18/09/13<br>INTITULE ETUDE : PROJET<br>AUTISME<br>**********************<br>N° COMMANDE :<br>DATE COMMANDE :<br>FOURNISSEUR N°006284<br>**********************	18/09/2013	1,00	pcs	0,00/1,00	0,00	0,00	0,00

Devise	Monant vente total	Remise totale	Frais	Montant HT	Montant TVA	Montant TTC
EUR	436,70	0,00	0,00	436,70	85,59	522,29

ANRH ■ SIEGE SOCIAL : 17, IMPASSE TRUILLOT ■ 75011 PARIS
Association Loi 1901 reconnue d'utilité publique par décret du 19 février 1968 - Code APE 8810 C

ANR
S E R V I C E S

Devis

Page : 2 de 4
Numéro : DV00000440-3
Date : 19/09/2013

Numéro d'article	Description	Date d'expédition	Quantité	Unité	Prix de vente	Remise %	Remise	Montant Vente
A070001048	FOURNITURES CLIENT ****************** * 1 Fichier word ou pdf pour édition consentement dupliqué * 1 Fichier word ou pdf pour questionnaire A 2 pages recto verso noir et blanc avec agrafage en haut à gauche * 1 Fichier word ou pdf pour questionnaire B 1 A4 recto noir et blanc * 1 Fichier word ou pdf pour questionnaire C 4 pages recto verso noir et blanc avec agrafage en haut à gauche * 1 Fichier word ou pdf pour questionnaire D 3 pages recto verso noir et blanc avec agrafage en haut à gauche * 1 Fichier word ou pdf pour questionnaire E 6 pages recto verso noir et blanc avec agrafage en haut à gauche * 1 Fichier word ou pdf pour questionnaire F 5 pages recto verso noir et blanc avec agrafage en haut à gauche	18/09/2013	1,00	pcs	0,00/1,00	0,00	0,00	0,00
A070001058	FOURNITURES ANR SERVICES ************************	18/09/2013	1,00	pcs	0,00/1,00	0,00	0,00	0,00
A070000024	CHU-PAPIER A4 80 GR BLANC	18/09/2013	5 000,00	pcs	0,08/10,00	0,00	0,00	40,00
A070001059	PRESTATIONS ************	18/09/2013	1,00	pcs	0,00/1,00	0,00	0,00	0,00
A070000692	CHU-DUPLICATA A4 DE 21 A 150 EX	18/09/2013	100,00	pcs	1,09/1,00	0,00	0,00	109,00
A070000686	CHU -EDITION A4 2 RECTO/VERSO N&BLx700 pour questionnaire A	18/09/2013	1 400,00	pcs	0,42/10,00	0,00	0,00	58,80

Devise	Monant vente total	Remise totale	Frais	Montant HT	Montant TVA	Montant TTC
EUR	436,70	0,00	0,00	436,70	85,59	522,29

ANRH ■ SIEGE SOCIAL : 17, IMPASSE TRUILLOT ■ 75011 PARIS
Association Loi 1901 reconnue d'utilité publique par décret du 19 février 1968 - Code APE 8810 C

ANR SERVICES

Devis

Page : 3 de 4
Numéro : DV00000440-3
Date : 19/09/2013

Numéro d'article	Description	Date d'expédition	Quantité	Unité	Prix de vente	Remise %	Remise	Montant Vente
A070000478	CHU-POSE 1 AGRAGE HAUT GAUCHE pour questionnaire A	18/09/2013	700,00	pcs	0,56/10,00	0,00	0,00	39,20
A070000685	CHU -EDITION A4 RECTO TEXTE NOIR x 300 pour questionnaire B	18/09/2013	300,00	pcs	0,21/10,00	0,00	0,00	6,30
A070000686	CHU -EDITION A4 4RECTO/VERSO N&BLx400 pour questionnaires C	18/09/2013	1 600,00	pcs	0,42/10,00	0,00	0,00	67,20
A070000478	CHU-POSE 1 AGRAGE HAUT GAUCHE pour questionnaire C	18/09/2013	400,00	pcs	0,56/10,00	0,00	0,00	22,40
A070000686	CHU -EDITION A4 3RECTO/VERSO N&BLx200 pour questionnaire D	18/09/2013	600,00	pcs	0,42/10,00	0,00	0,00	25,20
A070000478	CHU-POSE 1 AGRAGE HAUT GAUCHE pour questionnaire D	18/09/2013	200,00	pcs	0,56/10,00	0,00	0,00	11,20
A070000686	CHU -EDITION A4 6RECTO/VERSO N&BLx100 pour questionnaire E	18/09/2013	600,00	pcs	0,42/10,00	0,00	0,00	25,20
A070000478	CHU-POSE 1 AGRAGE HAUT GAUCHE pour questionnaire E	18/09/2013	100,00	pcs	0,56/10,00	0,00	0,00	5,60
A070000686	CHU -EDITION A4 5RECTO/VERSO N&BLx100 pour questionnaire F	18/09/2013	500,00	pcs	0,42/10,00	0,00	0,00	21,00

Devise	Monant vente total	Remise totale	Frais	Montant HT	Montant TVA	Montant TTC
EUR	436,70	0,00	0,00	436,70	85,59	522,29

ANRH ■ SIEGE SOCIAL : 17, IMPASSE TRUILLOT ■ 75011 PARIS
Association Loi 1901 reconnue d'utilité publique par décret du 19 février 1968 - Code APE 8810 C

ANR SERVICES

Devis

Page : 4 de 4
Numéro : DV00000440-3
Date : 19/09/2013

Numéro d'article	Description	Date d'expédition	Quantité	Unité	Prix de vente	Remise %	Remise	Montant Vente
A070000478	CHU-POSE 1 AGRAGE HAUT GAUCHE pour questionnaire F<br><br>********************<br>CONDITIONNEMENT CARTONS RECUP ANR<br>********************<br>LIVRAISON ANR :<br>MAISON DE LA RECHERCHE EN SANTE DE NANTES<br>MME FERRAND LEA<br>53 chaussée de la Madeleine<br>IMMEUBLE CAP OUEST<br>3EME ETAGE<br>NANTES<br>*******************	18/09/2013	100,00	pcs	0,56/10,00	0,00	0,00	5,60

Code TVA	% TVA	Base TVA	Montant TVA
FR_ENC_TN	19,60 %	436,70	85,59

Devise	Monant vente total	Remise totale	Frais	Montant HT	Montant TVA	Montant TTC
EUR	436,70	0,00	0,00	436,70	85,59	522,29

ANRH ■ SIEGE SOCIAL : 17, IMPASSE TRUILLOT ■ 75011 PARIS
Association Loi 1901 reconnue d'utilité publique par décret du 19 février 1968 - Code APE 8810 C
