# Supplementary material for: Migration is associated with baseline severity and progress over time in autism spectrum disorder: Evidence from a French prospective longitudinal study
Source: PLoS One. 2022 Oct 6;17(10):e0272693. doi: 10.1371/journal.pone.0272693 (PMC9536617; doi:10.1371/journal.pone.0272693)
Supplement: S3 File — (DOCX) [file pone.0272693.s003.docx]

| **SUPPUPPORTING INFORMATION**  **EPIGRAM STUDY** |
| --- |

**List of available supporting information**

S10 Table. Change over time for oral language examination

Sensitivity analysis regarding blind external experts’ ratings on PEP-3-R video recording

Sensitivity analysis taking into account possible confounding variables

S1 Table. Activities and Workshops Related to Educational (Proximal Autonomy) Approach

S2 Table. Activities and Workshops Related to Sensorimotor domain

S3 Table. Activities and Workshops Related to Socialization

S4 Table. Activities and Workshops Related to Communication

S5 Table. Activities and Workshops Related to Emotion, Anxiety, and Behaviour

S6 Table. Activities and Workshops Related to Pedagogic (cognition) Domain

S7 Table. Activities and Workshops Related to Family Resources

S8 Table. Activities Related to Somatic and Pharmacological Domain

S9 Table. Activities Related to Intra- and Extra-Institutional Joints

**Changes over time for oral language examination (ELO)**

We did not include the oral language examination data in the main manuscript because (1) many individuals without language showed a bottom effect and (2) language and communication were better captured by PEP-3-R corresponding subscores. Table S1 summarizes the ELO scores at baseline and 12 months. Linear mixed models were performed to assess the changes over time.

| **S10 Table. Oral language examination of the children included in the EPIGRAM (N=89): score at baseline and changes overtime (12 months)** | | | | | |
| --- | --- | --- | --- | --- | --- |
| Variable | Baseline | 12 months | Estimate | 95%CI | p-value |
| ELO reception lexicon:  mean (SD) [range] | 3.85 (5.01)  [0-15] | 6.89 (6.45)  [0-20] | 3.03 | [2.16; 3.91] | <0.001 |
| ELO production lexicon: mean (SD) [range] | 4.61 (7.91)  [0-31] | 10.29 (11.73)  [0-41] | 5.68 | [4.16; 7.21] | <0.001 |
| ELO comprehension:  mean (SD) [range] | 2.44 (4.31)  [0-15] | 6.36 (7.39)  [0-20] | 3.92 | [2.82;5.02] | <0.001 |
| ELO syntactic rule:  mean (SD) [range] | 0.90 (2.33)  [0-11] | 2.99 (4.38)  [0-18] | 2.09 | [1.41; 2.76] | <0.001 |

**Sensitivity analysis based on PEP-3-R cognitive verbal/preverbal and affective expression scores rated by blind external experts on PEP-3-R video recording**

***1. Descriptive statistics***

|  | | **M0 N=89** | **M12 N=89** |
| --- | --- | --- | --- |
| Percentile rank PEP-3-R cognitive verbal/preverbal score | N | 89 | 89 |
|  | Min-Max | [2.00;98.00] | [2.00;99.00] |
|  | Mean | 40.53 | 57.15 |
|  | Standard Deviation | 28.65 | 33.00 |
|  | Median | 36.00 | 57.00 |
|  | Q1-Q3 | [14.00;67.00] | [32.00;89.00] |
| Percentile rank PEP-3-R affective expression score | N | 89 | 89 |
|  | Min-Max | [2.00;41.00] | [2.00;63.00] |
|  | Mean | 17.47 | 20.07 |
|  | Standard Deviation | 12.65 | 13.41 |
|  | Median | 13.00 | 23.00 |
|  | Q1-Q3 | [6.00;26.00] | [9.00;26.00] |

| ***2. Linear mixed models***  The evolution of primary and secondary variables across time (from M0 to M12) was analysed using linear mixed models with a centre random effect. For each outcome variable, the estimated time effect, migration*time interaction effect, corresponding 95% CI and p-value were reported. |
| --- |
| PEP-3-R cognitive verbal/preverbal score |

**Figure 1. Boxplot of the PEP-3-R cognitive verbal/preverbal score expressed in percentile rank (N=89)**

Linear mixed model:

| **Effect** |  | **Estimate** | **IC 95%** | **p-value** |
| --- | --- | --- | --- | --- |
| PEP-3-R cognitive verbal/preverbal score | M12 vs M0 | 16.62 | [13.11; 20.13] | <.0001 |

The PEP-3-R cognitive verbal/preverbal score rated by blind external experts on the PEP-3-R video recording significantly improved over time.

| PEP-3-R affective expression score |
| --- |

**Figure 2. Boxplot of the PEP-3-R affective expression score expressed in percentile rank (N=89)**

Linear mixed model:

| **Effect** |  | **Estimate** | **IC 95%** | **p-value** |
| --- | --- | --- | --- | --- |
| Percentile rank affective expression score | M12 vs M0 | 2.60 | [0.83; 4.36] | 0.0045 |

The PEP-3-R affective expression score rated by blind external experts on the PEP-3-R video recording significantly improved over time

***3.* Conclusion**

Linear mixed models based on blind assessment evolved in the same way as those based on clinical assessment for the two primary outcome variables: PEP-3-R-CVP and PEP-3-R-AE scores.

**Sensitivity analysis regarding possible effect of socio-economic difficulties, age, autism severity, intensity of intervention, and center on migration**

***1. Context***

The recruitment centres of the EPIGRAM study were located all over France in both large and small cities (see figure 1). Due to the concentration of immigrant families in large cities in most Northern developed countries, there was an association between study cities and immigrant status. In addition, other variables might be influential such as socio-economic difficulties, age, autism severity at baseline and intensity of intervention. To ensure that the results presented on migration are not due to an interaction between migration and those other variables, we performed a second sensitivity analysis exploring the effect of migration, taking into account these variables in the models.

| ***2. Linear mixed models***  The evolution of the primary and secondary variables across time (from M0 to M12) was analysed using linear mixed models taking into account the following variables: socio-economic difficulties, age, autism severity at baseline, intensity of intervention and centres. For each outcome variable, the estimated time effect, migration*time interaction effect, corresponding 95% CI, and p-value were reported. The formulas are given below. |
| --- |

Linear mixed model without interaction:

Here, we explore the course of clinical characteristics over time (between M0 and M12) for the whole sample using linear mixed models without the (migration*time) interaction but taking into account possible confounding variables.

The model formula is

Score~ Time+Migration+Socio-economic difficulties+Age+ECAR-score-at-M0+Intervention-N +(1|Center/Subject)

|  | Estimate | 95% CI | Std. Error | Pr(>\|t\|) |
| --- | --- | --- | --- | --- |
| CARS | -0.55 | [-0.65;-0.45] | 0.05 | <0.001 |
| ECA-R global | -1.61 | [-1.8;-1.43] | 0.1 | <0.001 |
| ECA-R modulatory insufficiency | -0.15 | [-0.18;-0.12] | 0.02 | <0.001 |
| ECA-R relationship impairment | -0.91 | [-1.01;-0.81] | 0.05 | <0.001 |
| PEP-3-R verbal/preverbal cognition | 0.62 | [0.28;0.95] | 0.17 | 0.001 |
| PEP-3-R expressive language | 0.56 | [0.24;0.87] | 0.16 | 0.001 |
| PEP-3-R receptive language | 0.79 | [0.49;1.1] | 0.16 | <0.001 |
| PEP-3-R fine motor skills | 0.62 | [0.25;0.99] | 0.19 | 0.001 |
| PEP-3-R gross motor skills | 0.4 | [0;0.81] | 0.2 | 0.051 |
| PEP-3-R oculo-motor imitation | 0.61 | [0.24;0.99] | 0.19 | 0.002 |
| PEP-3 affective expression | 0.89 | [0.51;1.28] | 0.2 | <0.001 |
| PEP-3-R social reciprocity | 1.19 | [0.88;1.5] | 0.16 | <0.001 |
| PEP-3-R characteristic motor behaviours | 0.83 | [0.49;1.17] | 0.17 | <0.001 |
| PEP-3-R characteristic verbal behaviours | 1.28 | [0.83;1.73] | 0.23 | <0.001 |
| CAT communication | 0.61 | [0.38;0.83] | 0.11 | <0.001 |
| CAT motor skills | 0.58 | [0.29;0.88] | 0.15 | <0.001 |
| CAT inappropriate behaviours | 1.1 | [0.77;1.43] | 0.17 | <0.001 |

Linear mixed models show that all clinical characteristics, including the two primary variable outcomes (PEP3-R verbal/preverbal cognition and PEP3-R affective expression), improved during 12 months of the EPIGRAM study even after taking into account confounding variables (see Table 3 of the manuscript).

Linear mixed model with statistical interaction (migration*time):

Here, we analyse the impact of migration on the children’s progress over time (between M0 and M12) using linear mixed effect models with the (migration*time) interaction taking into account confounding variables.

Score~ Time+Migration+Socio-economic difficulties+Age+PEP-3R-CVP+ PEP-3R-CVB+ECAR-score-at-M0+Intervention-N+Migration*Time+Socio-economic difficulties*Time+Age*Time+PEP-3R-CVP*Time+ PEP-3R-CVB*Time+ECAR-score-at-M0*Time+Intervention-N*Time +(1|Center/Subject)

| Outcome | Migration*time Estimate | Std. Error | df | t value | Pr(>\|t\|) |
| --- | --- | --- | --- | --- | --- |
| **CARS** | **-0.222** | **0.099** | **81.000** | **-2.246** | **0.027** |
| ECA-R global | 0.101 | 0.195 | 348.000 | 0.516 | 0.606 |
| ECA-R modulatory insufficiency | 0.037 | 0.036 | 348.000 | 1.021 | 0.308 |
| ECA-R relationship impairment | -0.100 | 0.106 | 348.000 | -0.936 | 0.350 |
| PEP-3 verbal/preverbal cognition | -0.131 | 0.369 | 150.820 | -0.356 | 0.723 |
| PEP-3-R expressive language | -0.058 | 0.356 | 144.071 | -0.163 | 0.871 |
| PEP-3-R receptive language | 0.117 | 0.357 | 81.000 | 0.328 | 0.744 |
| PEP-3-R fine motor skills | 0.787 | 0.420 | 81.000 | 1.874 | 0.065 |
| PEP-3-R gross motor skills | 0.139 | 0.457 | 81.000 | 0.304 | 0.762 |
| PEP-3-R oculo-motor imitation | 0.354 | 0.436 | 81.002 | 0.812 | 0.419 |
| PEP-3 affective expression | -0.585 | 0.425 | 81.000 | -1.376 | 0.173 |
| PEP-3-R social reciprocity | -0.239 | 0.343 | 81.000 | -0.697 | 0.488 |
| PEP-3-R characteristic motor behaviours | 0.134 | 0.388 | 81.000 | 0.346 | 0.730 |
| **PEP-3-R characteristic verbal behaviours** | **-1.166** | **0.410** | **148.794** | **-2.845** | **0.005** |
| CAT communication | -0.170 | 0.251 | 81.000 | -0.678 | 0.499 |
| CAT motor skills | 0.386 | 0.340 | 81.000 | 1.135 | 0.260 |
| **CAT inappropriate behaviours** | **-0.657** | **0.333** | **81.000** | **-1.970** | **0.052** |

Linear mixed models showed that most clinical characteristics did not show any significant interaction, meaning that the range of improvement was similar in both groups over time. However, the models find the same significant interaction between migration and time (when the model also takes into account possible confounding variables and their interaction with time) for the two previous variables: characteristic verbal behaviours (PEP-3 R-CVB) and inappropriate behaviours category (PEP-3 R-CATCI)(see Table 4 of the manuscript). In addition, CARS was also significant. An explanation for the divergent CARS results is that some of the baseline variables mediate the effect of migration on CARS evolution. The migration effect from this model would represent the direct effect on CARS evolution. The migration effect from the model in table 4 of the manuscript would represent the total effect of on CARS evolution^[[1]](#footnote-1)^.

***3.* Conclusion**

We found that the linear mixed models produced the same results that are presented in the MS. This means that possible confounding variables did not introduce biases in the results presented for the migration status (see Table 4 of the manuscript).

| **S1 Table. Activities and Workshops Related to Educational (Proximal Autonomy) Approach** | | | |
| --- | --- | --- | --- |
| **General context** | | | |
| **Area – Dimension** | **Operating Assumptions – Target** | **Therapeutic Principles** | **Activities – Workshops** |
| Alterations in daily life actions such as feeding oneself, toilet cleanliness, dressing and undressing oneself, sphincter cleanliness. | Education promotes the autonomy of the child and brings him/her to a more social life.  Deficits of proximal and social autonomy.  Frequent dysfunction in imitations that remain very selective and fragmentary and can vanish as soon as the pattern changes.  Sensory peculiarities influence its autonomy. | To include the parents.  To simplify tasks and verbal instructions, structure, space and time.  To build a rich toolbox of tools based on different approaches and articulating them in interventions that are as adapted as possible to each child and carried out by practitioners who have a trusting relationship with him/her.  Depending on the age and the problem, the ABA, ESDM, or TEACCH approach can be chosen. | Activities to enable the child to establish a relationship of trust with the referring adult, building the reliability of the setting through basic activities.  Use of everyday life to facilitate generalization. |
| **Specific treatment proposals** | | | |
| **Nutrition** | Eating disorders can reflect the child's difficulty with relationships, acceptance of a newcomer, as well as his sensory particularities and specific motor/oral problems.  The establishment of a continued sense of existence remains problematic because of the difficulty in understanding what is specific and what comes from outside stimuli.  *Target*: orality | Sensorimotor approach of the oral apparatus  Working on symbolic representation during the educational dimension. | Daily activities, meals, setting the table, dinette set, cooking and therapeutic meal  Speech therapy with a specific approach to orality |
| **Dressing - undressing** | Refusal of certain clothes or on the contrary ask to tighten them against the body. Insensitivity to temperature changes.  Difficulty of coordination that hinders dressing. Lack of task organization or planification.  *Target*: wearing of clothes | Work on the body envelopment, on perceptions; rely on imitation.  Identification of the prerequisites necessary to allow progress (decomposition of the gestures), associated with an approach of the anguishes. | Daily activities on the child but also transposed on toys.  The paddling pool allows to approach with the child the conscience of his body, the dressing and undressing  The swimming pool and the learning of swimming complete this work on the body. |
| **Personal hygiene** | Disturbed awareness of one's body and well-being leading him to not take care of himself.  Tactile hypersensitivity, which reinforces his anxieties when he receives water on his head.  *Target*: hygiene | Pay attention to your body envelope.  Pay attention to hypersensitivity (vestibular, touch, proprioception) | Daily activities including at home |
| **Sphincter cleanliness** | Improvement in the acquisition of a better tone and body schema.  Sphincter control for opposition or stereotyped interests.  During toilet training, explore the possible anguish of losing a piece of oneself, the fear of falling into a hole.  *Target*: sphincter cleanliness | Identify developmental difficulties and anxieties.  Rely on rituals when they are not hindering.  Reassurance regarding anguish manifestations. | Daily activities including at home, toilet training.  ABA.  Occupation therapy or physiotherapy can help the child to become aware of his sensations (e.g., to retain and to evacuate). |
| **Proximal family autonomy** | First place of autonomy.  Sensory peculiarities, challenging behaviours, and difficulties in integrating the self compromise the acquisitions necessary for autonomy.  *Target*: daily initiatives. | Imitation is a step leading to the internalization of symbolic actions. Imitation allows adaptation to the human environment and constitutes a function of learning knowledge and know-how. | Daily activities, meals, setting the table, dinette game, cooking, therapeutic meals, toilet training. |
| **Social autonomy** | Social autonomy is linked to aspects of socialization and the decoding of the world around it.  Lack of initiative and knowledge of social rules and disorders of spatial orientation.  *Target*: *outward autonomy.* | Preparation indoors through activities and then outdoors. Setting in situation.  Development of social skills. | Workshops on social skills, educational games, outdoor activities (yard, outings, etc.), role-playing.  Therapeutic journey. |
| **Motor coordination and praxis** | Imitative deficits, motor coordination, motor planning and sequencing.  *Target*: Imitation and gross and fine motor skills. | Develop global and fine motor skills and coordination. | During motor workshop, develop tactile perceptions, refine visual perception, identify and locate objects, improve the holding of an object, improve motor coordination and skills (e.g., coordinate eye-hand).  Additionally, practice at home, at school.  Occupation therapy. |

| **S2 Table. Activities and Workshops Related to sensorimotor domain** | | | |
| --- | --- | --- | --- |
| **General context** | | | |
| **Area – Dimension** | **Operating Assumptions – Target** | **Therapeutic Principles** | **Activities – Workshops** |
| Sensorimotor domain and symptoms | Sensory integration dysfunctions can affect the pathways that lead from sensation to perception. Given action-perception coupling, they also contribute to achieve a clear representation of oneself and the world. Usually, this cannot occur without an adequate affective environment.  The deficits observed in motor planning, sensorimotor integration, and motor execution involve cortical and subcortical areas and cerebellar dysfunction. | Based on individual assessment, limit sensory stimulation that provokes irritability behaviours.  Establish a framework in time and space, allowing the child to find his or her bearings, not to be surprised.  Keep the framework accessible and understandable by the child.  Use the interest in using visual strategies, as children with ASD have better skills in this domain.  Promote motor installation (e.g., back trunk support) and a smooth caregiver relationship as they facilitate interaction (e.g., being aware of the child’s attention, availability, rhythm) | Sensorimotor workshops |
| **Specific treatment proposals** | | | |
| **Sensory domain** | Difficulties in integrating several sensory information. | Soothe/mitigate situations of over-stimulation. | Sensorimotor workshops |
| **Avoidance or repetitive search for stimulation** | Confusion in the perceptions of the perceptual channel (both intensity and quality).  *Target*: sensory modalities of reception or expression. | The sensory awakening workshops offer children different types of sensory experiences in order to help them perceive them, distinguish them, feel them, and move from one to the other. | Sensorimotor workshops  Occupational therapy |
| **Audio and vestibular stimulation** | Difficulty filtering and discriminating audio and vestibular perceptions affect response to audio/vestibular stimuli, orientation to stimuli, and being in balance in space.  *Target*: audio channel, equilibrium. | Variation of perceptions, progressive and modulated expression.  Use of visual investment, proprioception, and body pressure to compensate vestibular dysfunction.  Support on possible musical abilities. | Sensorimotor workshops focusing on sensory awakening, singing, melody, game with sounds, activities stimulating proprioception (e.g., percussion)  Occupational therapy |
| **Vision** | Interactive gazing and movement perception is avoided or a source of panic through the direct visual irritability-emotional link.  *Target*: vision. | Promote smooth representation from visual perception.  Distinguish focal vision and kinematic vision as investment and irritability may differ.  Work on oculo-motor and visual spatial coordination.  Work on joint attention. | Workshop assembly differentiation game  Book/album workshop  Orthoptics  Occupational therapy |
| **Haptic stimulation** | Need to soothe sensory irritation and hyperactivity.  Need for a safety envelope and a unified representation of the body.  *Target*: haptic stimulation. | The approaches with water, including the paddling pool, allow a more global work on the limits of the body, the tonus, the posture, the envelope (dressing/undressing, drying, wrapping), the awareness of the body representation. | Water games, paddling pool  Notification of pain expression  Occupational therapy |
| **Taste** | Immutability of taste, refusal to change, difficulty in biting or getting into the mouth.  Does not chew, swallows, fills up.  *Target*: oral stimulation. | The cooking workshop promotes experiences around touch, taste, smell and their global integration.  Promote accompaniment during therapeutic meals.  Work on the tactile sensitivity of the mouth. | Kitchen workshop  Propose stimulating game/activities to do at home with the parents  Speech therapy |
| **Smell** | Atypical preoccupations such as reconnaissance exploration or object identification through smelling.  *Target*: olfaction | Differentiation of smells, familiarization and association with the nature of objects. | Sensory workshop focusing on taste, smell |
| **Motor domain** | The non-integration of the body schema influences the representation of Body and Self.  Reducing multiaxial/multimodal sensory information enhances the acquisition of better coordination.  *Target*: posture, tone, coordination, locomotion. | Linking the different parts of the body (top/bottom, right/left).  Progressive and integrated body experiences. | Workshop focusing on exchanges with the proximal person (the referring caregiver), being physically together (other child...)  Motor games  Occupational therapy |
| **Coordination** | Eye-hand, right-left, top-bottom links have peculiarities that hinder coordination actions.  *Target*: coordination | Use of the body for progressively more complex activities following different postures according to the segments being worked on. | Motor skills workshop  Motor activities at school  Occupational therapy |
| **Tonus** | Global hypo- or hypertonia affect interaction and movement.  Hypo- or hypertonia can differ according to the segments and affect body representation.  *Target*: tone and posture | Implementation of the tonus in a supportive environment or in the relationship with interactive partners (e.g., pony). | Pony workshop, swimming pool, paddling pool |
| **Body and Self representation** | Disturbances in the integration of the body schema with sometimes an investment of certain body parts to the detriment of others, disturbances in the representation of space. Indifference between oneself and the other.  *Target*: gestures and motor interaction | More global work on the limits of the body, tonus, posture, wrapping (dressing/undressing, drying, wrapping), awareness of body representation, distinguishing oneself and others. | Workshop on differentiating self and others  Occupational therapy |
| **Toilet training difficulties** | In connection with motor coordination, the maturation of the sphincter but also their investment and the associated anxieties.  *Target*: cleanliness | Relationship with parents, knowledge of situations that alleviate or reinforce the dysfunction. | Activity or workshop on inputs/outputs.  Toilet training (see also educational approach) |

| **S3 Table. Activities and Workshops Related to Socialization** | | | |
| --- | --- | --- | --- |
| **General context** | | | |
| **Area – Dimension** | **Operating Assumptions – Target** | **Therapeutic Principles** | **Activities – Workshops** |
| Socialization, lack of initiation and reciprocity, inadequate responses, search for immutability.  Autistic withdrawal, resistance to change. | The child with autism rarely makes contact. He may be worried when approached. He lacks social interaction. He shows no empathy, does not express his affects (e.g., through prosody or facial expression) and has little ability to "read" the other person's emotional state. | Ensure that the child is part of a temporal and emotional story.  Role-playing and role-plays help the child to acquire social skills.  Improve the understanding of emotions and intentions through different supports and mediations. | Social skill workshop.  Focusing on the quality of exchanges, the understanding of social codes, the pleasure in the relationship through a certain number of "sequenced" situations, linked by the narrative, to the generalization of situations. |
| **Specific treatment proposals** | | | |
| **Interaction initiation, reception time** | The first moments of exchange aim to make the child aware of the existence of the other, which often involves a privileged relationship with a caregiver.  *Target*: identification of family and friends | Stable, possibly visual, relational cues and organization of time in sequences. | In all group activities and encounters. |
| **Interaction maintenance**  **e.g., the time of the meal** | The different supports and mediations improve the understanding of one's emotions, one's intentions and those of others.  *Target*: social relationship. | Develop expressive skills (modelling clay workshops, painting and other plastic arts...), cognitive skills (educational games, board games, reading workshop, storytelling...), role-playing.  Enables the acquisition of social skills and gives the child confidence in everyday life: individually, in groups, in themed activities with specific supports (social scenarios, social skills), inside the structure or outside (swimming pool, horseback riding, visits to museums, exhibitions, shops, etc.). | Role-playing workshop, social skills, educational games, outdoor activities (classes, outings, etc.), role-playing, mealtime.  Therapeutic journeys during which the different aspects of socialization are addressed. |
| **Meeting time with parents**  **Time to meet with other partners** | These times of exchanges must also be understood as serving to generalize and broaden the child's socialization.  *Target*: generalization. | Discuss the child in order to develop together the most appropriate adaptations or accommodations for the child according to his difficulties, attitudes and progresses, which may or may not be different depending on the place. | Exchanges with parents, with the school environment |

| **S4 Table. Activities and Workshops Related to Communication** | | | |
| --- | --- | --- | --- |
| **General context** | | | |
| **Area – Dimension** | **Operating Assumptions – Target** | **Therapeutic Principles** | **Activities – Workshops** |
| Communication | Communicative language is related to linguistic acquisition but also the development and differentiation between the self and the other.  The precursor mechanisms of language are hindered (e.g., intentionality detector, the direction of gaze, shared attention). | The child needs anticipation and predictability, with a stable support to which he can refer to himself.  Promote a developmental approach of communication.  Build the basis for triadic representations. | Favour visual supports.  Speech therapy  Activities to enable the child to establish a relationship of trust with the referring adult. |
| **Specific treatment proposals** | | | |
| **Nonverbal social signs: gazing, pointing, imitation, joint attention, iconic and arbitrary gestures, make-believe.** | Non-verbal modes of communication are impeded. These alterations contribute to a global disorder concerning communication and symbolic functions.  *Target*: Nonverbal communication. | Implementation of means adapted according to the child's developmental level, emotional and attentional capacities. | Through all activities  Everyday Game workshop |
| **Shared attention**  Level 1 | Achievement of eye contact, shared interest, synchrony.  *Target*: shared attention. | Observe to share his interests, imitate him, balance the initiations of communication of the child and those of the adult. | Everyday Game workshop  Social routines, songs |
| Level 2 | Lack of joint attention.  Difficulty in sharing emotions, intermodality, affective tuning, understanding the implicit, continuity and representations.  *Target*: Tuning. | Take into account the non-verbal aspects of language, the conditions of the emergence of language in its functions of dialogue, sharing of emotions, in a permanent intermodality between visual, sound, tonic and tactile exchanges. | Mime workshops, nursery rhymes, puppets, character games  Tales (sketches or simple stories), storytelling |
| **Intention to communicate**  Level 3 | Intention to communicate present but without the ability to express oneself orally in an efficient manner.  Target: alternative communication | Alternative or augmentative communication modes for answering a question, describing an event, naming, making a request. | Incremental means such as PECS, Makaton outside of speech therapy sessions B3  Use of multimedia supports. |
| Level 4 | Emerging intention with present expressive capacity.  Fragility or weak perception of communication intention.  *Target*: intention and expression | Observe, wait to perceive communication intentions and to seize opportunities. | Everyday Game workshop |
| **Pragmatic** | The child makes little use of pointing, gestures, posture, prosody or other social signals for the purpose of communication.  *Target*: pragmatic | Support the child by using non-verbal language when addressing. | Everyday Game workshop  Mime workshops, nursery rhymes, puppets, character games |
| **Verbal communication – linguistics** | Children with autism have a pervasive deficit in the development of language and show many difficulties common to specific language disorders.  *Target*: language | Adapt to the child's language level, have relevant language.  Stimulate verbal communication through other pathways (e.g., written language).  Use alternatives to oral communication | Speech therapy  Everyday Game workshop |
| **Receptive language** | Apparent indifference to questions asked and difficulty understanding figurative language.  Target: comprehension | Adapt to the child's language level, have relevant language. | Speech therapy  Everyday Game workshop |
| **Expressive language**  Level 1: basic | Environmental features are only integrated secondarily. Hypothesis of discrimination defect in speech sounds, pitch, hearing hypersensitivity. Procedural deficit  *Target*: basic verbal language | Support oral expression: repeat what he says, rephrase, reconstruct. | Speech therapy  Workshops focusing on oral language |
| **Expressive language**  Level 2: elaborate | The linguistic dimensions of language are difficult to improve in non-verbal children (e.g., phonology, grammatical rules, semantics).  *Target*: elaborate verbal language | Reconstruct the language by syntactic and/or semantic extension. | Workshops focusing on language expression and comprehension |
| **Conversation, speech turns** | The difficulty in initiating, maintaining and ending conversation reflects the difficulty in using language in socialization.  *Target*: Turn taking in communication | Follow the rhythm, alternate turns of speech, make pauses, give back the initiative, elaborate, until reaching conversational reciprocity. | Mime workshops, nursery rhymes, puppets, character games  Tales (sketches or simple stories), storytelling |
| **Verbalization and representation of emotions and anguish** | The child has a certain level of expression and representation and exhibits anxiety.  *Target*: defensive mechanisms related to anxieties. | The psychotherapy sessions contribute to the reciprocity of exchanges and the expression of internal feelings. Putting into words the emotional movements and attenuate defence mechanisms. | Psychotherapy |

| **S5 Table. Activities and Workshops Related to Emotion, Anxiety, and Behaviour** | | | |
| --- | --- | --- | --- |
| **General context** | | | |
| **Areas – Dimension** | **Operating Assumptions – Target** | **Therapeutic Principles** | **Activities – Workshops** |
| Emotion, Anxiety, Oppositional behaviours, Disorganizations,  Functional manifestations, Depression, Stereotypes | Anxiety and emotional experiences are internal states within the child. Behaviours are external manifestations of it. Behaviours can be a consequence of multiple factors, which must be explored to understand the function and meaning for the child. | Create a secure environment | Fixed markers and different forms of wrapping (physical, sound), structuring of time and space. |
| **Specific treatment proposals** | | | |
| **Emotions within relationships** | In autism, difficulties in giving shape to feelings, in identifying emotions, both one's own and those of others, may influence social interaction.  *Target*: emotions within social interaction. | Allow the child to identify, distinguish between his emotions and those of others, learn to modulate and adapt his responses. | Emotion recognition workshops  Psychotherapy |
| **Affect, Anxiety, Emotion regulation, Depression** | Anxiety often results from sensory or motor peculiarities as well as disturbances in cognition and emotional regulation.  Depressive affects have a self-deprecating effect.  *Target*: affect, anxiety. | Taking into account the child’s anxiety, emotional manifestations and behaviour in all aspects of management by articulating the therapeutic and educational components. | Create a secure containing environment  Expression workshop, discrimination and emotion regulation workshop  Occupational therapy  Psychotherapy |
| **Stereotypical behaviours** | Defect of a secure containing environment that expresses itself in stereotypical behaviours, self or hetero-aggressiveness.  *Target*: stereotypical behaviours. | All stereotyping is not a behavioural disorder. It becomes one when it is persisting and pervasive, preventing interest in new activities and learning. Stereotypes can also be a source of pleasure and release and may have a recuperative function. | Activities of setting in arranged situation, with body dimension |
| **Challenging behaviours** | Relationship between the behaviour and its environment or context.  *Target*: recognition of triggering contexts. | Observation and analysis of the sequence of challenging behaviours.  Search and identify the cause or trigger.  Identify both positive and negative reinforcements. | Internal and external articulation between professionals and parents.  Calming response in language, posture and environment. Restriction of negative reinforcement. Introduction of alternative behaviour with positive reinforcement. |
| **Disorganizations** | Difficulty giving shape to feelings, identifying emotions, one's own or those of others.  *Target*: disorganization, potential mechanism. | To enter into communication with the child, it is essential to alleviate these disorganizations and crises by creating a secure containing environment made of trusting relationships, fixed reference points and sometimes different forms of wrapping. | Fitted scenario activities with body dimension  Fitting out spaces, calming time  Workshops or activities focusing on capacity  Occupational therapy, wrapping  Workshop on emotion identification  Psychotherapy  Drug prescription after medical advice |
| **Functional events**  **Sleep, diet, intestinal transit.** | Anxiety and depression can manifest themselves in functional impairment.  *Target*: symptoms, origin. | Observe whether the anxiety is confined to the conditions of the function or more general aspects. | Fitted scenario activities with body dimension |
| **Pain** | The child with ASD does not express pain directly, can do it in a paradoxical way.  Target: pain, origin. | Identify behavioural problems that are indicative of pain.  Help the child express pain and painful areas. | Investigation of the origin  Drug prescription after medical advice |

| **S6 Table. Activities and Workshops Related to Pedagogic (cognition) Domain** | | | |
| --- | --- | --- | --- |
| **General context** | | | |
| **Area – Dimension** | **Operating Hypotheses – Targets** | **Therapeutic Principles** | **Activities – Workshops** |
| Pedagogic (cognition) domain. | The development of categorization, cognitive skills, associative learning, and metaphorization is affected.  Executive functions are dysfunctional (attention, working memory, mental flexibility, planning, control and regulation of action). | Implementation of a pedagogy adapted according to cognitive, neuropsychological, sensory, psycho-affective and personal specificities.  Close collaboration between the care service and the school in different modalities. | Structuring of time, structuring of space, use of pictograms and other concrete supports, specific software and computer programs. |
| **Specific treatment proposals** | | | |
| **Cognitive dimension** | Difficulty in generalizing.  Sequential processing of information.  Difficulty in understanding the implicit.  Lack of flexibility.  Impairments in attention and working memory.  *Target*: cognitive functioning | Respect Vygotsky’s zone of proximal development theory  Adaptation of supports and lightening of tasks  Adaptation of school materials  Adaptation of the language to the level of comprehension, indications given with a slow fluency.  Simplifying learning and targeting success.  Respecting the child's rhythm of functioning  Use positive reinforcements | Cognitive workshop.  Classroom integrated in the care unit  Computer software  Individual cognitive remediation |
| **Schooling** | The schooling is adapted according to the child’s curriculum. Both difficulties and potentialities need to be assessed  *Target*: learning acquisition | Inclusion (in regular school) or integration (time-sharing), associated partial schooling (at the day hospital site)  The close collaboration between the health care service and the school is carried out in different ways. | Educational agenda based on French Ministry for National Education’s objectives and following principles of the Handiscol plan  School Inclusion  Specialized class |
| **Psycho-affective dimension** | Certain psychic processes are necessary for the learning situation (e.g., recognizing oneself as different from the other; accepting what comes from the other; recognizing one's own shortcomings without feeling disorganized).  *Target*: inclusion in the learning situation. | Implementation of means adapted to the child's developmental level, emotional and attention capacities.  Moments of withdrawal, breaks, help in the recognition of physiological needs to prevent states of disorganization. | Cognitive activities adapted to allow the child to establish a relationship of trust with the teacher, building the reliability of the framework through basic activities.  Favour learning by success rather than trial and error.  Sequence of adaptive pedagogy in day hospital (cognitive workshop) |
| **Sensory dimension** | Access to learning presupposes good sensory and sensory-motor integration.  *Target*: sensory integration | Adapt the learning context to the sensorimotor skills and sensory sensitivities.  Coherent and reassuring environment. | Adaptation of the environment: posture, space, working place, limitation of noises.  Imitation games, embedding, puzzles, sorting objects, works on categorization, etc. |

| **S7 Table. Activities and Workshops Related to Family Resources** | | | |
| --- | --- | --- | --- |
| **General context** | | | |
| **Area – Dimension** | **Operating Hypotheses – Targets** | **Therapeutic Principles** | **Activities – Workshops** |
| Family Resources  The care of autistic children can only evolve in optimal conditions if it is carried out with the participation of and a therapeutic alliance with the parents. | Parents have real knowledge about their child, which the caregiver needs to build on. | Our role is to inform, train, support, substantiate, and promote the understanding of the autistic processes and behaviours presented by the child in order to find, with the parents, strategies to improve them. | Regular meetings with parents |
| **Specific treatment proposals** | | | |
| **Alliance** | Its quality is based on the sharing of common concerns.  *Target*: partnership with parents. | Consultations, visits to living areas, meetings with partners. Place of the referring caregiver. | Consultations  Home visits |
| **Diagnostic path** | Synthesis of functional explorations.  Diagnostic announcement includes the elements of the main diagnosis, associated disorders, developmental profile, and recommendations.  *Target*: clinical analysis and restitution. | Explained routes with planned consultations.  Place of the attending physician (general practitioner or paediatrician).  Follow-up by the referring caregiver who ensures consistency.  Functional explorations, articulation between professionals. | Assessments, consultations, home visits  Partner exchanges  Telephone exchanges, mailings  Clinical meetings  Interview with social worker |
| **Individualized therapeutic project** | The modalities and objectives are developed based on the child's curriculum and the parents' expectations.  *Target*: individualized care project. | Provide appropriate information.  Allow to meet other families.  To accompany in the strategies of communication, socialization and behavioural adjustment of the child on a daily basis.  To guide in the steps.  Propose a space to express questions as parents. | Consultations  Use parental subjective assessment scale to enhanced feeling of participation  Discussion of the therapeutic plan |
| **Integrative Practices** | ICP is a multidisciplinary system with a set of coordinated interventions. The child is a subject in relation to his parents and his usual living environment. ICP is based on complementary perspectives: developmental, physical, psychopathological and physiological, and associates a plurality of interventions (therapeutic, educational, pedagogical)  *Target*: individualized care project. | Exchanges on the deployment of the interventions, their objectives, their articulations, and their evolutions.  Supervision  Regular summary update | Discussion of the project, regular items  Informal exchanges  Taking into account important inter-current events |
| **Training and information** | The parental group’s objectives are to break the feeling of isolation, to propose a reciprocal support, to exchange experiences, to project into the future.  *Target*: to improve skills. | Provide information and/or concrete elements improving communication and behavioural disorders.  Accompany in the strategies of communication, socialization and behavioural adjustment of the child in daily life.  Training to improve communication (e.g., PACT) | Parents’ or siblings’ speaking group  Training in Specific Techniques (PACT)  ESDM training and supervision  Training and information groups |

ICP= integrative care practice; PACT=paediatric autism communication therapy; ESDM=early start Denver model.

| **S8 Table. Activities Related to Somatic and Pharmacological Domain** | | | |
| --- | --- | --- | --- |
| **General context** | | | |
| **Area – Dimension** | **Operating Assumptions – Target** | **Therapeutic Principles** | **Activities** |
| Somatic and pharmacological needs  Difficulty for the person with autism to access medical care. | Medical comorbidities are frequent.  Frequency of sleep and eating disorders.  Inability to express one's feelings. Clinical expression may be idiosyncratic and difficult to understand.  *Target*: Medical conditions | Contribute to improve access to care for children with autism.  Raising awareness among health care professionals and networking for recognition of possible medical comorbidities and access to care. | Recognize and treat medical conditions if needed  Also prevention with regular follow-up (e.g., dental, paediatric, ophthalmologic consultations) |
| **Specific treatment proposals** | | | |
| **Somatic monitoring** | In front of any unexplained behavioural disorder or any sudden change in behaviour, pain or somatic condition must be considered.  *Target*: vigilance on medical condition | Creation of a "checklist" for behavioural changes.  Search for sources of discomfort and pain.  Getting the child used to care (hygiene, visits to the doctor, the dentist).  Detect hypersensitivity phenomena, pain.  Detect epileptic disorders. | Organize rapid access for medical care  Getting the child used to routine medical visits  Recognizing manifestations of pain |
| **Pharmaceutical follow-up** | For behavioural disorders, treatment is proposed after a detailed analysis of the nature of the disorders and their function.  They can be disabling and can compromise social inclusion and family life.  Sleep disorders are frequent.  Comorbidities are examined.  Target: adapted prescription (benefit/risk) | Benefit/risk discussion with professionals and parents. Take into account the child's suffering, his or her unavailability for care, the exhaustion of the entourage, the risk of social eviction.  Associated psychological disorders are evaluated.  Associated somatic disorders are treated in conjunction with the specialist and the attending physician.  Regular monitoring of the treatment at all levels. Its interest must be regularly re-evaluated in order to avoid maintaining a treatment that is not very effective. | Make necessary drug prescriptions |

| **S9 Table. Activities Related to Intra- and Extra-Institutional Joints** | | | |
| --- | --- | --- | --- |
| **General context** | | | |
| **Area – Dimension** | **Operating Assumptions – Target** | **Therapeutic Principles** | **Activities** |
| Intra- and extra-institutional joints  The care mobilizes multiple stakeholders, of different functions, practical and theoretical references, and several institutional groups, leading to complex articulations. | Articulations are the cement of integrative practices. They ensure the coherence and cohesion of care, interventions, pedagogy and family support.  *Target*: building and gathering around the child. | The interventions of each professional are part of a therapeutic system. The place of each is defined in terms of complementarity. Attention is given to the child in its development and fulfilment, to the parents in their ability to support their child, and in avoiding dispersion and confusion. |  |
| **Specific treatment proposals** | | | |
| **Internal articulations** | The institution has the value of a psychological container with organizational value.  Institutional work is a connective tissue that connects the individual to his or her own history and that of others.  *Target*: Coordination of the intervening parties. | Maintain the coherence of the team through times of reflection and elaboration (e.g., recording, supervision, synthesis).  Use predictable, regular framework.  Try when producing familiar daily activities to bring bearable deviations that allow the child to tame the novelty and its reality. | Synthesis  Recovery, supervision  Clinical meetings (between nurses/educators with or without another professional)  Staff training |
| **External articulations** | The care of autistic children, regardless of internal articulations, is evolving towards an open system where there is not one institution with total control of the care but a partnership, constantly to be renewed, between care, educational and pedagogical action.  *Target*: harmonization between partners and family. | The meetings are preceded by a request for agreement with the parents; they are also an opportunity for the family and the team to refine each one’s respective places.  A chronology and hierarchy in the objectives of school integration is progressively clarified.  Child psychiatry focuses on functions and development whereas the socio-educative team focuses on social relationship and adaptation to the environment.  Complementary assessment may be required to Third Level Expert team.  Prepare, with the family, all the requirements to obtain National Support for Handicapped Children.  Support by user associations and transmission of knowledge and practices.  Care, preventive actions, complementary assessments by PMIs, treating physicians, paediatric services. | Telephone exchanges, mailings  Meetings  Special attention for reciprocal links  Inform and accompany parents |

1. Westreich, D., & Greenland, S. (2013). The table 2 fallacy: presenting and interpreting confounder and modifier coefficients. *American journal of epidemiology*, *177*(4), 292-298. [↑](#footnote-ref-1)
